# The burden of cirrhosis and other chronic liver diseases due to hepatitis B in children and adolescents: results from global burden of disease study 2019

**DOI:** 10.3389/fpubh.2023.1315392

**Published:** 2023-12-22

**Authors:** Chenyang Huang, Yaxin Wu, Chao Zhang, Dong Ji, Fu-Sheng Wang

**Affiliations:** ^1^Medical School of Chinese PLA, Beijing, China; ^2^Department of Infectious Diseases, Fifth Medical Center of Chinese PLA General Hospital, National Clinical Research Center for Infectious Diseases, Beijing, China; ^3^Senior Department of Hepatology, Fifth Medical Center of Chinese PLA General Hospital, Beijing, China

**Keywords:** hepatitis B, cirrhosis, children, adolescents, incidence, prevalence

## Abstract

**Background:**

The global burden of cirrhosis and other chronic liver diseases due to hepatitis B (collectively referred to as hepatitis B-associated cirrhosis in this paper) in children and adolescents must be understood and investigated.

**Methods:**

Data were extracted from the GBD database, and calculations were performed at global, regional, and national level. We calculate the incidence, prevalence, and disability-adjusted life years (DALYs) and annual average percentage changes (AAPCs).

**Findings:**

Globally, the prevalent cases of children and adolescents with hepatitis B-associated cirrhosis decreased from 125,053.98 × 10^3 in 1990 to 46,400.33 × 10^3 in 2019. Compared with 1990, the incidence rate of cirrhosis increased in low (95.51%) and low-middle SDI areas (26.47%), whereas it decreased in other SDI areas. The AAPC of incidence has increased in low-middle SDI areas (AAPC 0.12 [95% CI: 0.04–0.20]). At the regional level, the East Asia region has experienced the largest reduction. Conversely, Western Sub-Saharan Africa was the most serious region. Notably, South Asia was the only region where the AAPC of cirrhosis incidence (AAPC 0.77 [95% CI, 0.68–0.86]) increased.

**Conclusion:**

Globally, the overall burden of hepatitis B-associated cirrhosis in children and adolescents has declined significantly, but the number of cirrhosis incidence cases in low-middle and low-SDI areas has increased. The incidence in South Asia is rising, and the burden on Africa remains serious. Prevention and treatment of hepatitis B-associated cirrhosis in children and adolescents should not be ignored.

## Introduction

Liver cirrhosis and other chronic liver diseases is a serious public health problem, and its incidence, morbidity, and mortality have increased in recent years (1). In 2019, 1691.0 million cases and 1.5 million deaths from liver cirrhosis were reported worldwide, accounting for 2.4% of global deaths (2). Moreover, liver cirrhosis is one of the top 20 causes of reduced disability-adjusted life years (DALYs) and quality-adjusted life years (QALYs), accounting for 1.6 and 2.1% of the global burden (3), respectively. Hepatitis B-associated cirrhosis is the most common cause of liver cirrhosis currently (4), accounting for 42% of cases worldwide. Although the global prevalence rate of hepatitis B has shown a downward trend with multiple measures such as hepatitis B vaccination, maternal and infant blocking, and the improvement of hepatitis B diagnosis and treatment ability, the ASIR (age-standardized incidence rate) in the middle SDI (sociodemographic index), low SDI, and middle-low SDI regions has increased by 0.13% annually on average, and the model predicts that under the current strategy, the mortality caused by hepatitis B will increase by 39% from 2015 to 2030 (5). The health of children and adolescents is a core goal of the Sustainable Development Goals of the United Nations and is expected to reach the highest health standards by 2030 (6).

James et al. (7) analyzed the global burden of nonalcoholic fatty liver disease and chronic liver disease among adolescents and young adults between 1990 and 2019. Moreover, Global Burden of Disease (GBD) 2019 Hepatitis B Collaborators (8) systematically reported the global, national, and regional burdens of hepatitis B from 1990 to 2019 and found that the prevalence of hepatitis B surface antigens in infants and children younger than 5 years declined by 77% between 1990 and 2019. Recently, Zhang et al. (9) also reports the global, regional and national burdens of cirrhosis in children and adolescents. However, the above research did not depth analyze specifically for burden of hepatitis B-associated cirrhosis in children and adolescents at regional, country, SDI, sex and age group level and the trends changes.

Therefore, we conducted a comprehensive and detailed analysis of the incidence, prevalence, and DALYs of hepatitis B-associated liver cirrhosis in 204 countries from 2009 to 2019 in children and adolescents. We stratified the trends by age, sex, region, and SDI group. We aimed to analyze the disease burden of children and adolescents with hepatitis B-associated cirrhosis based on the GBD database to provide a scientific reference for health policy optimization, clinical diagnosis and treatment guideline development, and medical resource allocation.

## Methods

### Data sources

Data were sourced from the Institute for Health Metrics and Evaluation at the University of Washington, United States.^1^ The website currently includes data on the global burden of diseases and health diseases for 29 years, from 1990 to 2019. This GBD database covers the most comprehensive information on diseases, risks, deaths, and disabilities related to diseases and is constantly updating the data. Through the Global Health Data Exchange query tool,^2^ we extracted the incidence, prevalence, and DALYs of 204 countries, five areas divided by the SDI, and 21 regions divided by different geographical regions. The population and disease burden estimation methodology has been previously described (10). The diagnosis of cirrhosis and other liver diseases due to hepatitis B in GBD 2019 is based GBD cause code (B.4.1), hereafter collectively referred to as hepatitis B-associated cirrhosis, this general term (hepatitis B-associated cirrhosis) refers to a study by GBD 2017 Cirrhosis Collaborators (11). All rates were defined per 10,000 population. The complete list of predictive covariates used in the model can be found in Supplementary Table S1. The 95% uncertainty intervals (UI) were defined using the 25th and 97th values of ordered 1,000 estimates based on the GBD algorithm (12).

### Definition of indicators

DALYs refer to the total number of years of lost healthy life from onset to death, including years of lost life (YLLs) due to premature mortality and years of lost health life due to disability (YLDs) (DALYs = YLLs + YLDs). SDI is a comprehensive indicator of the development status of a country or region, evaluated based on data such as the overall fertility rate of women <25 years old, the average education level of women aged >15, and *per capita* income. Each country (or region) has a corresponding SDI value ranging from 0 to 1. The larger the value, the better the development status of the country (Supplementary Table S2). According to the level of development, the SDI is divided into low (<0.20), low-middle (0.20–0.39), middle (0.40–0.59), high-middle (0.60–0.79), and high (>0.80) (13).

### Statistics

The Global Burden of Disease (GBD) is an approach to global descriptive epidemiology. It is a systematic, scientific effort to quantify the comparative magnitude of health loss due to diseases, injuries, and risk factors by age, sex, and geography for specific points in time. In this study: (i) We used DisMod-MR 2.1 and the Bayesian meta-regression tool to pool incidence, prevalence, and DALYs to describe epidemiological characteristics, and the specific calculation method has been described and reported before (14). We calculated the incidence, prevalence, and DALYs of cirrhosis for 1990 and 2019, respectively. (ii) The joinpoint regression model has been widely applied in analyzing disease trend changes (15). The joinpoint regression model consists of linear and logarithmic linear models. If the dependent variable followed a normal distribution, a linear model was selected. If the dependent variable followed an exponential or Poisson distribution, a logarithmic linear model was suitable. In this study, we selected a logarithmic, linear model to depict the trends in disease changes over the past 29 years. Using the grid search method and Monte Carlo Permutation test steps, we calculated the annual percent change (APC) and average annual percent change (AAPC), the maximum number of joinpoints was 5. The specific calculation method has been explained in detail in a previous study (16). (iii) We also calculated the percentage of relative changes in incidence and prevalence from 1990 to 2019. (iv) The burden of hepatitis B-associated cirrhosis in different age groups, SDI, regions, and countries was also assessed. All statistical analyses were performed using R software (R 4.2.3, Institute for Statistics and Mathematics) and Joinpoint trend analysis software (Version 5.0.2). A *p*-value of <0.05 indicated statistical significance. This study followed the Guidelines for Accurate and Transparent Health Estimates Reporting for cross-sectional studies (17) (Supplementary Table S3).

## Results

### Global trends of hepatitis B-associated cirrhosis from 1990 to 2019

**Figure 1:**
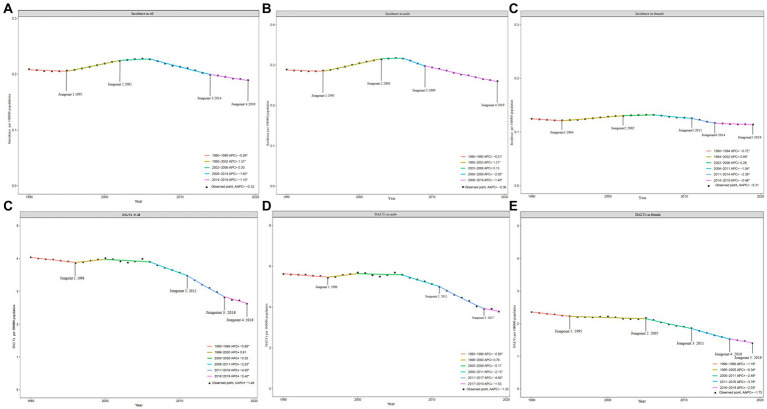
Joinpoint regression of hepatitis B-associated cirrhosis incidence and DALYs in children and adolescents during 1990–2019. **(A)** Incidence in all; **(B)** Incidence in male; **(C)** Incidence in female; **(D)** DALYs in all; **(E)** DALYs in male; and **(F)** DALYs in female.

Compared with 1990, the 2019 incidence rate slightly decreased, and incident cases slightly increased, prevalence rate and cases of hepatitis B-associated cirrhosis among children and adolescents worldwide significantly declined. Specifically, the prevalent cases of children and adolescents with hepatitis B cirrhosis decreased from 125,053.98 × 10^3 (95% UI: 107,448.42 × 10^3–144,188.35 × 10^3) in 1990 to 46,400.33 × 10^3 (95% UI: 39,395.9 × 10^3–54,065.23 × 10^3) in 2019, and the prevalence rate decreased from 5,500.33 (95% UI: 4,725.98–6,341.93) in 1990 to 1,798.97 (95% UI: 1527.40–2096.14) in 2019 (Table 1). Globally, the hepatitis B-associated AAPC incidence decreased from 1990 to 2019 (AAPC:-0.33 [95% confidence interval (CI): −0.42 to −0.25]). The joinpoint regression model identified significant changes in the incidence of cirrhosis in 1995, 2002, 2014, and 2019 (Figure 1A; Supplementary Table S4). The prevalence of hepatitis B-associated cirrhosis decreased between 1990 and 2019 (AAPC: −3.80 [95% CI:-3.95 to −3.65]). Hepatitis B-associated cirrhosis DALYs also significantly decreased (AAPC: −1.46 [95% CI: −1.75 to −1.17]) between 1990 and 2019. The joinpoint regression model identified significant changes in the DALYs of patients with cirrhosis in 1996, 2011, 2016, and 2019 (Figure 1D; Supplementary Table S5).

**Table 1:** Incidence and prevalence of hepatitis B-associated cirrhosis in children and adolescents by sex, age, SDI, and region in 1990 and 2019.

Characteristics	1990		2019			1990		2019		
	Incident cases No (95% UI)	Incidence Rate per 100,000 No. (95% UI)	Incident cases No. (95% UI)	Incidence Rate per 100,000 No. (95% UI)	No. change (%)	Prevalent cases No×10^3^ (95% UI)	Prevalence Rate per 100,000 No. (95% UI)	Prevalent cases No×10^3^ (95% UI)	Prevalence Rate per 100,000 No. (95% UI)	No. change (%)
Global	4751.02 (2186.40–9172.97)	0.21 (0.10–0.40)	4878.57 (2198.43–9463.74)	0.19 (0.09–0.37)	2.68	125053.98 (107448.42–144188.35)	5500.33 (4725.98–6341.93)	46400.33 (39395.9–54065.23)	1798.97 (1527.40–2096.14)	−62.90
Sex										
Male	3367.57 (1563.75–6425.86)	0.29 (0.13–0.55)	3456.65 (1546.50–6696.32)	0.26 (0.12–0.50)	2.64	72150.62 (62112.77–83041.48)	6194.44 (5332.64–7129.46)	26932.33 (22875.37–31273.46)	2025.80 (1720.65–2352.34)	−62.67
Female	1383.44 (641.99–2713.60)	0.12 (0.06–0.24)	1421.92 (650.39–2776.40)	0.11 (0.05–0.22)	2.78	52903.36 (45262.12–61026.16)	4771.20 (4082.06–5503.77)	19468.01 (16462.17–22795.83)	1557.68 (1317.17–1823.94)	−97.06
Age group										
<5 years	658.25 (282.84–1238.71)	0.10 (0.04–0.20)	531.75 (228.31–1000.47)	0.08 (0.03–0.15)	−19.21	27535.00 (22700.81–32636.10)	4356.07 (3591.24–5161.41)	6699.12 (5451.80–8035.25)	1010.66 (822.49–1212.24)	−75.67
5–9 years	926.11 (346.14–1800.79)	0.15 (0.06–0.31)	900.74 (364.08–1747.24)	0.14 (0.06–0.27)	−2.73	31839.96 (27000.78–37438.87)	5441.22 (4615.44–6398.03)	9479.62 (7890.15–11259.92)	1447.93 (1205.149–1719.85)	−70.23
10–14 years	1151.81 (356.35–2390.07)	0.21 (0.06–0.44)	1297.95 (428.44–2647.72)	0.20 (0.07–0.41)	12.68	31953.52 (27537.59–36918.85)	5953.84 (5131.01–6878.99)	12656.90 (10679.24–14885.22)	1970.91 (1662.95–2317.90)	−60.40
15–19 years	2014.85 (565.76–4655.80)	0.39 (0.11–0.90)	2148.14 (623.43–5018.30)	0.35 (0.10–0.81)	6.61	33724.96 (29391.54–38555.99)	6490.52 (5656.53–7420.27)	17564.69 (14996.30–20419.42)	2835.11 (2420.55–3295.90)	−47.92
SDI										
Low SDI	914.83 (419.46–1713.62)	0.31 (0.14–0.58)	1787.22 (822.76–3395.64)	0.30 (0.14–0.57)	95.36	21502.49 (18000.98–25061.61)	7279.83 (6094.37–8484.80)	21360.61 (17858.88–24879.54)	3578.21 (2991.62–4167.69)	−0.66
Low-middle SDI	1304.08 (610.31–2476.12)	0.23 (0.11–0.43)	1649.78 (739.87–3194.71)	0.24 (0.11–0.46)	26.51	27285.30 (23151.33–31861.38)	4781.94 (4057.43–5583.92)	13513.84 (11489.88–15840.49)	1943.43 (1652.33–2278.03)	−50.47
Middle SDI	1521.61 (704.51–2917.28)	0.20 (0.09–0.38)	1022.45 (462.94–1946.96)	0.14 (0.06–0.26)	−32.80	52627.90 (45493.59–60188.41)	6862.20 (5931.95–7848.02)	9005.70 (76431.88–10532.82)	1222.69 (1037.70–1430.02)	−82.89
High-middle SDI	680.94 (291.85–1341.04)	0.17 (0.07–0.33)	306.17 (128.90–614.51)	0.09 (0.04–0.19)	−55.04	20102.25 (17390.39–22910.64)	4960.46 (4291.27–5653.46)	1652.56 (14020.88–1937.894)	503.88 (427.51–590.88)	−91.78
High SDI	328.08 (135.86–634.15)	0.14 (0.06–0.27)	111.62 (43.87–237.26)	0.05 (0.02–0.11)	−65.98	3487.78 (3027.54–4006.46)	1489.46 (1292.91–1710.97)	844.55 (70455.88–1015.353)	382.45 (319.05–459.79)	−75.79
Region										
Southeast Asia	434.54 (172.25–900.58)	0.20 (0.08–0.41)	224.26 (82.83–482.01)	0.10 (0.04–0.21)	−48.39	13710.47 (11579.51–16030.22)	6195.81 (5232.82–7244.11)	3820.73 (32354.9–4505.208)	1694.77 (1435.17–1998.38)	−72.13
Eastern Europe	71.42 (29.85–142.41)	0.11 (0.04–0.21)	41.60 (16.76–86.95)	0.09 (0.04–0.18)	−41.75	774.58 (647.45–912.33)	1150.67 (961.82–1355.30)	66.78 (56423.9–78.32085)	140.82 (118.98–165.15)	−91.39
Central Asia	78.34 (28.28–166.16)	0.25 (0.09–0.53)	61.12 (18.32–140.94)	0.18 (0.05–0.41)	−21.98	1414.18 (1058.52–1715.07)	4483.51 (3355.94–5437.45)	241.68 (19475.9–283.4313)	711.19 (573.11–834.05)	−82.91
Central Europe	68.86 (25.34–143.95)	0.18 (0.07–0.37)	25.16 (9.27–53.75)	0.11 (0.04–0.23)	−63.46	390.37 (325.65–454.95)	1012.36 (844.52–1179.85)	29.75 (24652.9–35.08288)	127.18 (105.37–149.95)	−92.38
Oceania	5.60 (2.02–11.93)	0.17 (0.06–0.36)	5.65 (1.88–12.79)	0.09 (0.03–0.21)	0.009	251.56 (184.72–298.68)	7599.42 (5580.22–9023.10)	171.91 (14207.9–194.9693)	2814.39 (2325.88–3191.84)	−31.66
East Asia	1015.40 (436.35–1976.46)	0.22 (0.09–0.42)	191.21 (79.25–394.89)	0.06 (0.03–0.13)	−81.17	50936.00 (43918.28–58193.87)	10943.03 (9435.36–12502.31)	2859.31 (24162.9–3356.563)	919.72 (777.21–1079.67)	−94.39
High-income Asia Pacific	241.79 (102.42–461.84)	0.48 (0.20–0.92)	66.75 (27.38–132.28)	0.21 (0.08–0.41)	−72.39	1347.25 (1148.60–1578.05)	2673.96 (2279.68–3132.04)	436.30 (36260.9–532.5449)	1350.92 (1122.74–1648.91)	−67.62
Southern Latin America	8.25 (2.37–20.65)	0.04 (0.01–0.11)	5.72 (1.49–14.62)	0.03 (0.01–0.07)	−30.67	58.02 (41.29–74.82)	299.46 (213.11–386.20)	14.91 (11189.9–18.57403)	74.69 (56.03–93.01)	−74.30
Caribbean	5.07 (1.50–12.45)	0.03 (0.01–0.08)	4.22 (1.14–10.54)	0.03 (0.01–0.07)	−16.77	124.51 (97.91–150.81)	825.22 (648.91–999.54)	80.77 (64188.9–97.63316)	520.04 (413.26–628.59)	−35.13
Western Europe	49.08 (16.98–113.28)	0.05 (0.02–0.12)	27.00 (8.91–62.62)	0.03 (0.01–0.07)	−44.99	721.97 (595.97–869.20)	733.67 (605.63–883.29)	233.93 (19293.9–281.1326)	253.58 (209.14–304.74)	−67.60
Australasia	3.20 (1.01–7.78)	0.05 (0.02–0.12)	1.94 (0.62–4.76)	0.03 (0.01–0.07)	−39.38	97.15 (82.18–115.50)	1548.31 (1309.74–1840.70)	10.43 (8973.9–12.18193)	144.79 (124.54–169.06)	−89.26
North Africa and Middle East	279.76 (109.45–580.44)	0.16 (0.06–0.32)	252.37 (86.81–563.83)	0.11 (0.04–0.25)	−9.79	7706.71 (6668.37–8755.49)	4274.51 (3698.59–4856.21)	2512.60 (20604.9–2982.561)	1098.19 (900.57–1303.59)	−67.40
Tropical Latin America	88.00 (37.18–172.86)	0.13 (0.05–0.25)	21.80 (8.01–46.93)	0.03 (0.01–0.07)	−75.23	2656.02 (2203.24–3233.13)	3814.29 (3164.05–4643.07)	163.28 (13241.9–200.2437)	244.38 (198.18–299.70)	−93.85
South Asia	1312.86 (634.36–2400.85)	0.24 (0.12–0.44)	2061.13 (937.14–3884.47)	0.30 (0.13–0.56)	57.00	16753.03 (14021.24–20045.86)	3056.79 (2558.34–3657.60)	11372.41 (96114.9–13488.11)	1637.43 (1383.89–1942.06)	−32.12
Central Latin America	19.57 (7.93–39.92)	0.02 (0.01–0.05)	9.63 (3.21–22.41)	0.01 (0.00–0.03)	−50.79	822.61 (618.39–1072.96)	999.62 (751.46–1303.84)	177.41 (13544.9–222.7077)	202.88 (154.88–254.67)	−78.43
Andean Latin America	7.30 (2.29–16.81)	0.04 (0.01–0.09)	5.84 (1.42–14.09)	0.02 (0.01–0.06)	−2.00	69.28 (55.69–82.79)	362.03 (291.01–432.67)	22.18 (18934.9–26.14722)	93.66 (79.96–110.42)	−268.37
High-income North America	10.06 (3.57–22.34)	0.01 (0.00–0.03)	5.15 (1.74–11.67)	0.01 (0.00–0.01)	−48.81	374.50 (302.68–454.16)	459.78 (371.61–557.58)	115.43 (95092.9–139.7235)	128.34 (105.73–155.35)	−72.09
Southern Sub-Saharan Africa	64.30 (29.81–120.99)	0.25 (0.11–0.46)	35.64 (15.13–68.48)	0.12 (0.05–0.22)	−44.57	2312.26 (2053.67–2567.72)	8822.75 (7836.08–9797.49)	748.06 (66752.9–827.7094)	2443.55 (2180.49–2703.73)	−67.65
Central Sub-Saharan Africa	118.53 (45.46–241.23)	0.38 (0.14–0.77)	218.46 (81.10–455.12)	0.31 (0.11–0.64)	84.31	2546.77 (2066.53–3046.90)	8080.53 (6556.79–9667.36)	3182.90 (26296.9–3777.434)	4472.81 (3695.31–5308.29)	24.98
Eastern Sub-Saharan Africa	222.67 (96.91–433.68)	0.20 (0.09–0.39)	426.09 (184.87–831.48)	0.19 (0.08–0.37)	91.35	7755.93 (6478.14–9177.46)	7010.56 (5855.58–8295.48)	5308.95 (44664.9–6233.752)	2375.13 (1998.22–2788.87)	−31.55
Western Sub-Saharan Africa	646.40 (310.18–1195.53)	0.60 (0.29–1.11)	1187.83 (559.84–2225.61)	0.48 (0.23–0.90)	83.76	14230.79 (12125.99–16328.89)	13257.55 (11296.69–15212.16)	14830.58 (12517.9–17235.24)	5973.48 (5041.98–6942.03)	4.21

UI uncertainty intervals.

### Hepatitis B-associated cirrhosis trends by sex

The incidence rate and cases of males were significantly higher than those of females by more than two-fold in 1990 and 2019 (Table 1). In 2019, the prevalent cases of liver cirrhosis in males was 26,932.33× 10^3 (95% UI: 22,875.37–31,273.46), while in females, it was 19468.01 × 10^3 (95% UI: 16,462.17 – 22,795.83). Although male (AAPC = −0.36, *p* < 0.01) and female (AAPC = −0.31, *p* < 0.01) incidence decreased from 1990 to 2019, the male rates were slightly lower than the female rates (Table 2). The results of the joinpoint model showed that the changing trend of the incidence of males and females was similar to that of females, indicating a “rise-down-rise” trend (Figures 1B,C). Conversely, from 1990 to 2019, the decrease in prevalence among females (AAPC = −0.382, *p* < 0.01) was higher than that among males (AAPC = −0.378, *p* < 0.01). Both males and females showed a significant decrease in DALYs (Figures 1E,F); however, this decrease was greater in males than in females (AAPC: −1.75 [95% CI: −1.96 to −1.53] vs. −1.35 [95% CI: −1.69 to −1.00], Table 2).

**Table 2:** The AAPC of Incidence, Prevalence, and DALYs of hepatitis B-associated cirrhosis in children and adolescents from 1990 to 2019 by sex, SDI, and region.

Characteristics	Incidence		Prevalence		DALYs	
	AAPC (95% CI)	*p* value	AAPC (95% CI)	*p* value	AAPC (95% CI)	*p* value
Global	−0.33 (−0.42, −0.25)	< 0.01	−3.80 (−3.95 to −3.65)	< 0.01	−1.46 (−1.75, −1.17)	< 0.01
Sex						< 0.01
Male	−0.36 (−0.49 to −0.23)	< 0.01	−3.78 (−3.93 to −3.64)	< 0.01	−1.75 (−1.96 to −1.53)	< 0.01
Female	−0.31 (−0.41 to −0.22)	< 0.01	−3.82 (−3.98 to −3.66)	< 0.01	−1.35 (−1.69 to −1.00)	< 0.01
SDI						< 0.01
Low SDI	−0.1 (−0.16, −0.04)	< 0.01	−2.48 (−2.64 to −2.31)	< 0.01	−1.65 (−1.79, −1.52)	< 0.01
Low-middle SDI	0.12 (0.04, 0.20)	< 0.01	−3.03 (−3.16 to −2.91)	< 0.01	−1.73 (−2.11, −1.34)	< 0.01
Middle SDI	−1.21 (−1.32, −1.11)	< 0.01	−5.84 (−6.11 to −5.58)	< 0.01	−2.28 (−2.63, −1.92)	< 0.01
High-middle SDI	−1.95 (−2.16, −1.74)	< 0.01	−7.76 (−8.46 to −7.06)	< 0.01	−3.11 (−3.38, −2.85)	< 0.01
High SDI	−3.45 (−3.52, −3.37)	< 0.01	−4.62 (−4.81 to −4.43)	< 0.01	−8.13 (−8.53, −7.72)	< 0.01
Region						
Southeast Asia	−2.36 (−2.54, −2.18)	< 0.01	−4.4 (−4.62 to −4.19)	< 0.01	−2.81 (−2.94, −2.69)	< 0.01
Eastern Europe	−0.63 (−0.86, −0.4)	< 0.01	−7.18 (−7.74 to −6.62)	< 0.01	−2.72 (−4.01, −1.4)	< 0.01
Central Asia	−1.18 (−1.47, −0.9)	< 0.01	−6.21 (−6.41 to −6.01)	< 0.01	−2.11 (−2.51, −1.71)	< 0.01
Central Europe	−1.65 (−1.97, −1.33)	< 0.01	−6.98 (−7.5 to −6.45)	< 0.01	−4.62 (−5.15, −4.09)	< 0.01
Oceania	−2.07 (−2.27, −1.88)	< 0.01	−3.35 (−3.46 to −3.24)	< 0.01	−2.42 (−2.56, −2.27)	< 0.01
East Asia	−4.27 (−4.55, −3.99)	< 0.01	−8.32 (−8.98 to −7.65)	< 0.01	−6.71 (−6.91, −6.51)	< 0.01
High-income Asia Pacific	−2.84 (−2.96 to −2.72)	< 0.01	−2.35 (−2.55 to −2.16)	< 0.01	−8.29 (−9.1 to −7.48)	< 0.01
Southern Latin America	−1.44 (−1.69, −1.18)	< 0.01	−4.81 (−5.24 to −4.38)	< 0.01	−3.25 (−3.57, −2.92)	< 0.01
Caribbean	−0.71 (−0.83, −0.58)	< 0.01	−1.73 (−2.33 to −1.13)	< 0.01	−2.14 (−2.34, −1.94)	< 0.01
Western Europe	−1.82 (−1.95, −1.69)	< 0.01	−3.6 (−3.73 to −3.47)	< 0.01	−4.43 (−4.92, −3.95)	< 0.01
Australasia	−2.16 (−2.3 to −2.03)	< 0.01	−7.88 (−8.45 to −7.31)	< 0.01	−3.75 (−4.49 to −3.01)	< 0.01
North Africa and Middle East	−1.18 (−1.29, −1.07)	< 0.01	−4.64 (−4.84 to −4.45)	< 0.01	−3.48 (−3.64, −3.33)	< 0.01
Tropical Latin America	−4.56 (−4.64, −4.49)	< 0.01	−9.22 (−9.91 to −8.53)	< 0.01	−5.1 (−5.58, −4.61)	< 0.01
South Asia	0.77 (0.68, 0.86)	< 0.01	−2.12 (−2.2 to −2.05)	< 0.01	−1.55 (−1.91, −1.19)	< 0.01
Central Latin America	−2.64 (−2.81, −2.47)	< 0.01	−5.48 (−6.04 to −4.91)	< 0.01	−3.36 (−3.67, −3.04)	< 0.01
Andean Latin America	−1.53 (−1.63, −1.42)	< 0.01	−4.58 (−4.81 to −4.35)	< 0.01	−4.76 (−5.04, −4.47)	< 0.01
High-income North America	−2.61 (−2.79 to −2.44)	< 0.01	−4.25 (−4.5 to −4)	< 0.01	−3.33 (−3.74 to −2.91)	< 0.01
Southern Sub-Saharan Africa	−2.52 (−2.63, −2.42)	< 0.01	−4.35 (−4.78 to −3.91)	< 0.01	−3.24 (−3.82, −2.66)	< 0.01
Central Sub-Saharan Africa	−0.81 (−1.11, −0.5)	< 0.01	−2.03 (−2.22 to −1.85)	< 0.01	−2.71 (−2.93, −2.49)	< 0.01
Eastern Sub-Saharan Africa	−0.19 (−0.25, −0.14)	< 0.01	−3.7 (−3.98 to −3.43)	< 0.01	−2.02 (−2.27, −1.78)	< 0.01
Western Sub-Saharan Africa	−0.73 (−0.89, −0.57)	< 0.01	−2.69 (−2.83 to −2.56)	< 0.01	−1.97 (−2.19, −1.75)	< 0.01

AAPC average annual percentage changes, DALYs disability-adjusted life-years.

### Hepatitis B-associated cirrhosis trends by age group

Compared with 1990, the incidence rate in children and adolescents in all age groups (< 5 years, 5–9 years, 10–14 years, 15–19 years) decreased in 2019, with the most apparent decrease (20%) in the age group of <5 years. However, the incident cases in the 10–14 age group increased. Whether at the global level or in different regions and SDI regions, the incidence rate in the 15–19 age group was always the highest (Supplementary Figures S1–S4). Patients aged 10–14 and 15–19 years accounted for 70.6% incident cases of children and adolescents in 2019. Especially in high-income North America, the incident cases for the 15–19 years group was as high as 76.2%, followed by tropical Latin America (67.2%) and East Asia (64.2%). Regarding prevalent cases, patients aged 15–19 years and 10–14 years accounted for 65.2% of the cases. The prevalence in the 15–19 age group was also the highest, whether at the global level or in different regions and SDI regions (Supplementary Figures S5–S8). DALYs in different age groups all showed a decreasing trend (Supplementary Figures S9–S11).

### Hepatitis B-associated cirrhosis in different countries and territories

**Figure 2:**
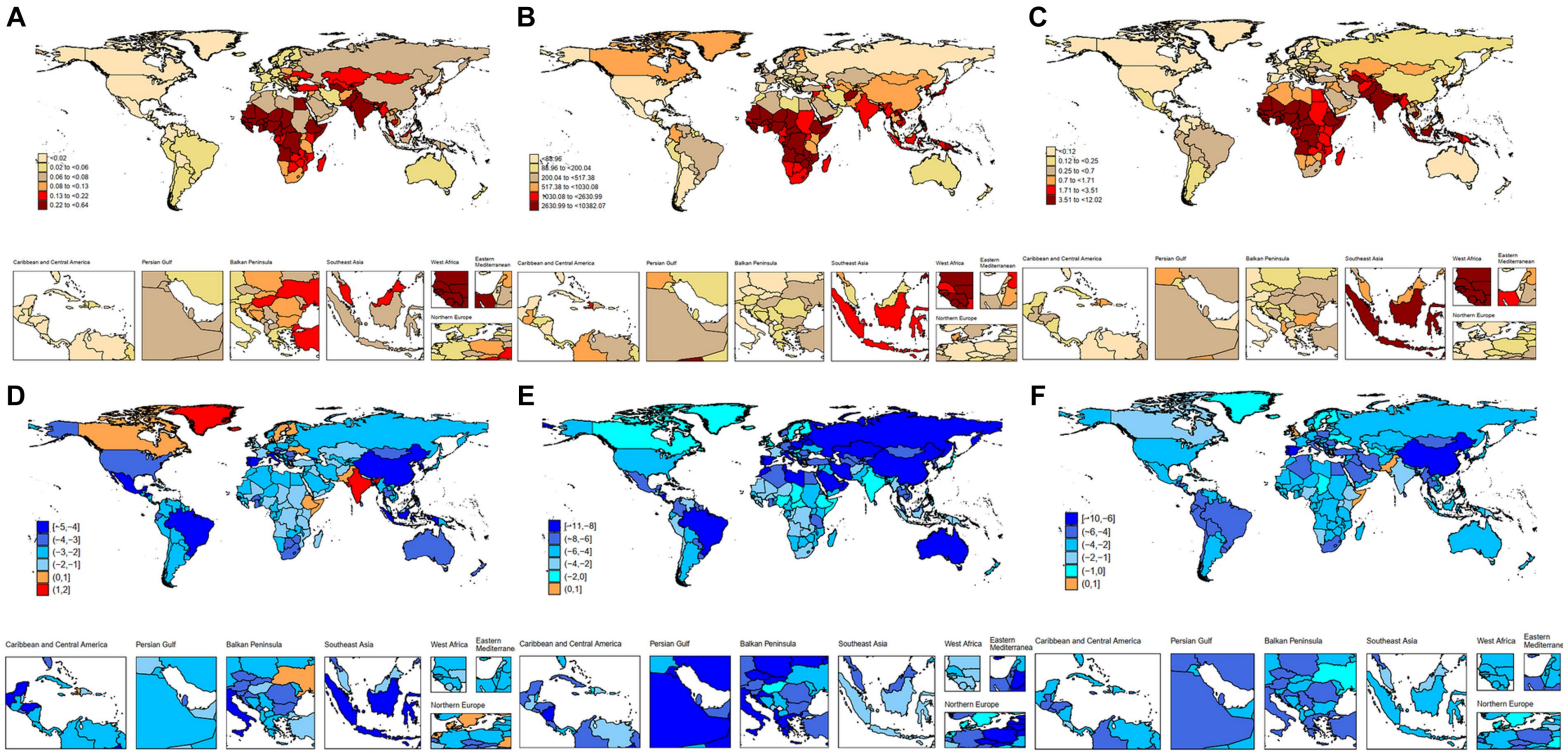
Incidence, prevalence and DALYs of hepatitis B-associated cirrhosis among children and adolescents in 204 countries and territories. **(A)** Incidence of hepatitis B-associated cirrhosis in 2019; **(B)** Prevalence of hepatitis B-associated cirrhosis in 2019; **(C)** DALYs of hepatitis B-associated cirrhosis in 2019; **(D)** AAPC in incidence of hepatitis B-associated cirrhosis between 1990 and 2019; **(E)** AAPC in prevalence of hepatitis B-associated cirrhosis between 1990 and 2019; and **(F)** AAPC in DALYs of hepatitis B-associated cirrhosis between 1990 and 2019.

In 2019, the relatively high incidence rate was mainly concentrated in Africa and parts of Asia. With the highest incidence, Sao Tome and Principe (0.64 [95% CI: 0.23–1.32] per 100,000 population) was 100 times higher than the United States (0.005 [95% CI: 0.002–0.012] per 100,000 population), which had the lowest incidence rate (Figure 2A; Supplementary Table S6). From 1990 to 2019, except for 18 countries, including India (AAPC = 1.12, *p* < 0.01), South Sudan (AAPC = 1.06, *p* < 0.01), and Finland (AAPC = 0.92, *p* < 0.01), the incidence of other countries showed a downward trend. The countries with the most significant decline were Brazil (AAPC = −4.68, *p* < 0.01), China (AAPC = −4.57, *p* < 0.01), and Indonesia (AAPC = −4.16, *p* < 0.01) (Figure 2D; Supplementary Table S7).

In 2019, Chad (10,382.06 [95% CI: 7,762.72–12,612.92] per 100,000 population), which had the highest prevalence, was 300 times higher than Uruguay (31.32 [95% CI: 23.09–40.83] per 100,000 population), which had the lowest prevalence (Figure 2B; Supplementary Table S8). From 1990 to 2019, except for the Northern Mariana Islands, all other countries showed an down trend, with Bhutan (AAPC = −10.47, *p* < 0.01), Oman (AAPC = −10.15, *p* < 0.01), and Saudi Arabia (AAPC = −10.11, *p* < 0.01) showing the most significant decline (Figure 2E; Supplementary Table S7). In 2019, countries with relatively high DALYs were concentrated in Africa and some Asian regions, with Chad (12.02 [95% CI: 4.91–21.76] per 100,000 population) having the highest DALYs and the United States having the lowest (0.02 [95% CI: 0.01–0.03] per 100,000 population) (Figure 2C; Supplementary Table S9). The countries with the most significant decline were South Korea (AAPC = −9.08, *p* < 0.01), Portugal (AAPC = −8.60, *p* < 0.01), and China (AAPC = −6.87, *p* < 0.01) (Figure 2F; Supplementary Table S7).

### Hepatitis B-associated cirrhosis trends by SDI and region

**Figure 3:**
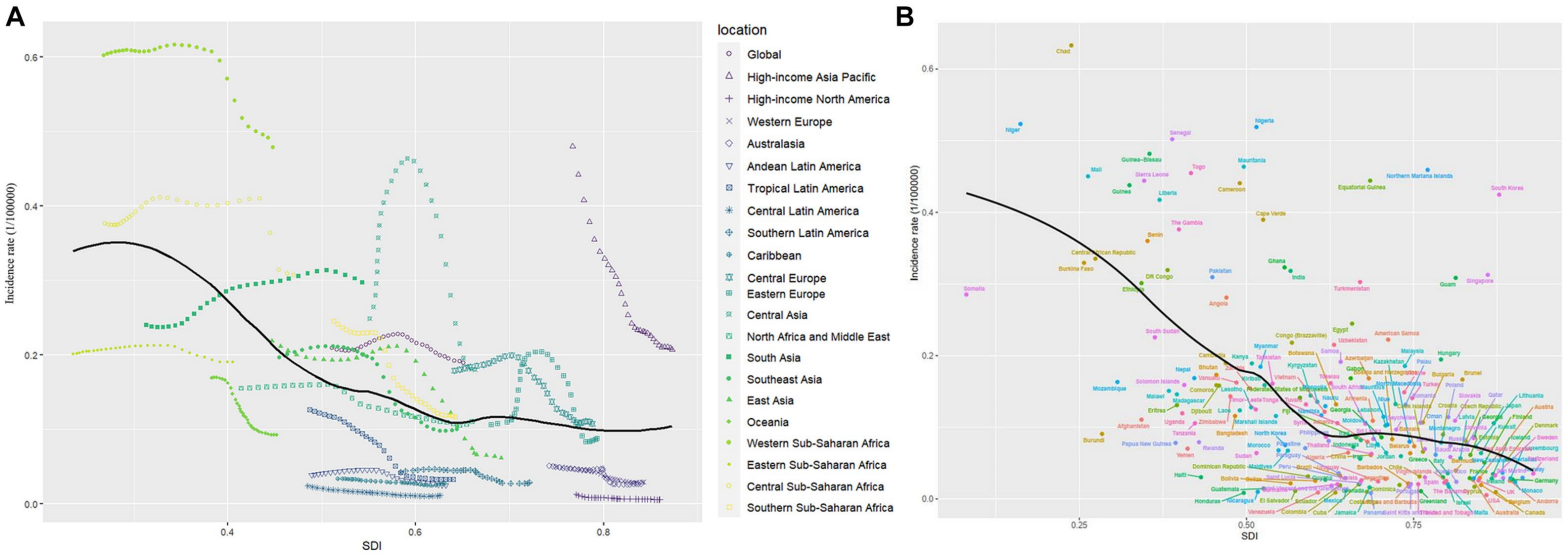
Global, regional, country and territory level burden of hepatitis B-associated cirrhosis in children and adolescents by SDI, from 1990 to 2019. **(A)** Incidence per 100,000 population in global and 21 regions; **(B)** Incidence per 100,000 population in global and 204 countries and territories.

In 2019, except for the increased incidence rate trend in low-middle SDI areas (0.24 [95% CI: 0.11–0.46] per 100,000 population), other SDI areas showed a downward trend (Table 1). Notably, the AAPC incidence increased in the low-middle SDI areas (AAPC = 0.12, *p* < 0.01) and decreased in other SDI areas. The number of incident cases and prevalent cases decreased in all SDI areas. DALYs decreased in all SDI regions, and the decrease in DALYs was more pronounced as the SDI increased. The AAPC of DALYs for low SDI areas and high SDI areas were − 1.65 (95% CI: −1.79 to – 1.52) and − 8.13 (95% CI: −8.53 to −7.72), respectively (Table 2). The Figures 3A,B shows the relationship between regions with different SDI levels and changes in incidence. In addition to showing an increasing and then decreasing trend in the curved Central Asian region, the incidence and SDI showed a negative correlation, and a significantly higher incidence was observed in areas with a low SDI, especially in the Western Sahara region.

**Figure 4:**
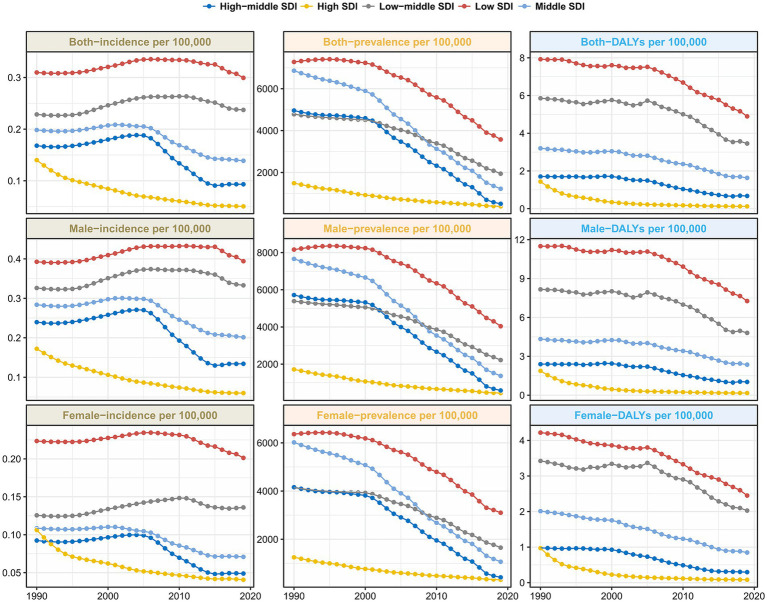
Trends of incidence rate, prevalence rate and DALY rate of hepatitis B-associated cirrhosis in SDI area from 1990 to 2019.

**Figure 5:**
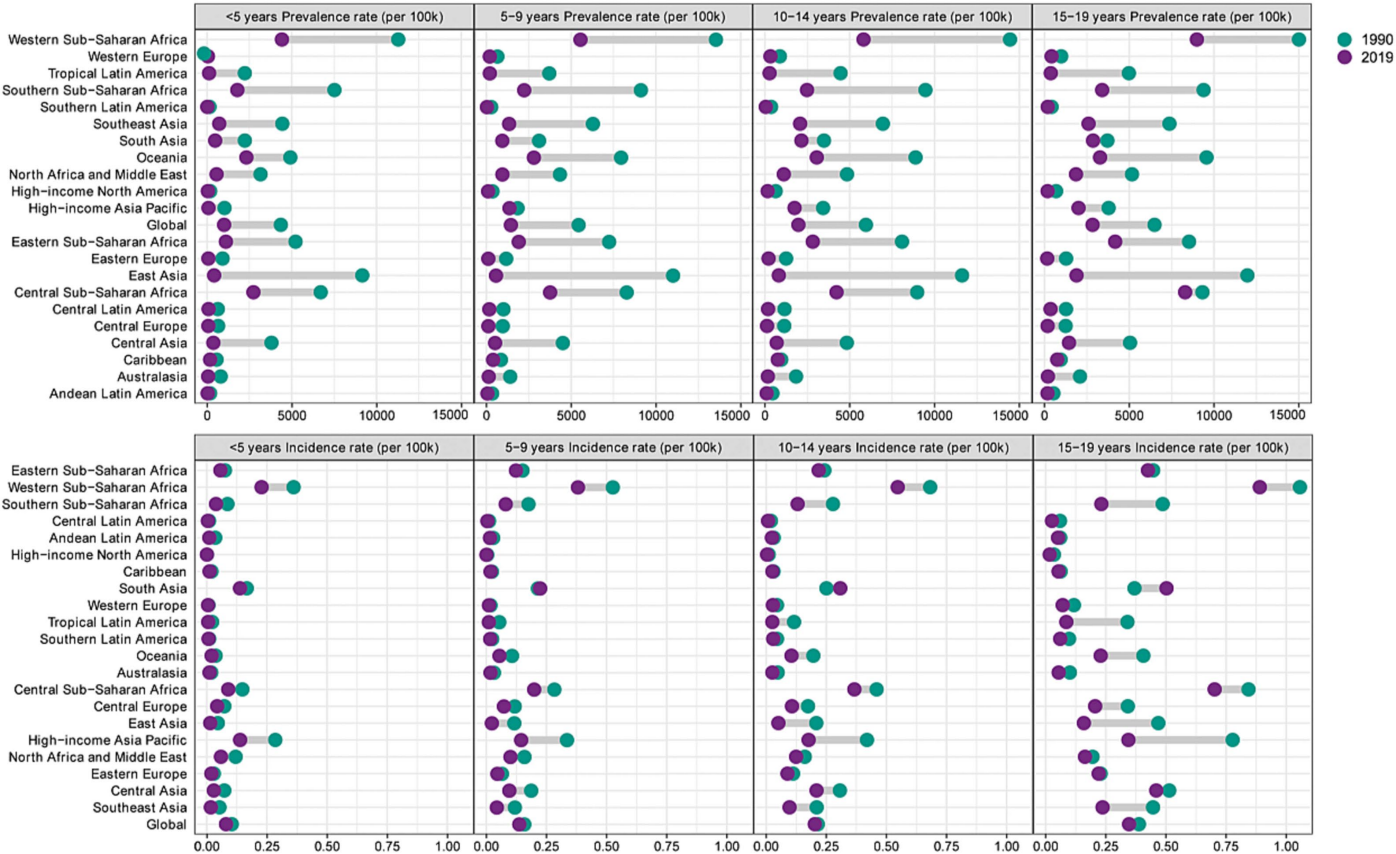
Incidence rate of hepatitis B-associated in different age groups at the regional level in 1990 and 2019.

The incidence rate in the different SDI regions was significantly different. The low-SDI and low-middle SDI regions fluctuated; the others showed a downward trend annually (Figure 4). In terms of prevalence rateand DALY levels, all regions showed a decreasing trend annually, with the highest incidence rate and prevalence rate in 2019 being Western Sub-Saharan Africa, which had 0.48 per 100,000 population and 5,973.48 per 100,000 population, respectively (Table 1). The incidence declined significantly in the high-income Asian Pacific and East Asia regions (Figure 5). Especially in the age group of 15–19 years old, the high-income Asian Pacific region showed the most apparent decline in the global regional incidence. East Asia experienced the largest reduction number of prevalent cases (from 50,936.00 × 10^3 [95% UI: 43,918.28–58,193.87] to 2,859.31 × 10^3 [95% UI: 24,162.9–3,356.563]). In 2019, the Western Sahara region had both the highest incident cases (1,187.83× 10^3 [95% UI: 559.84–2,225.61]) and prevalent cases (14,830.58 [95% UI: 12,517.9-17,235.24]) at the regional level. Compared to 1990, the number of incident cases in Western Sahara increased by 83.76% in 2019 and the incidence rate rate is up to 0.48 per 100,000. Moreover, compared with 1990, the Western Sahara region is also the only region with an increase in the number of prevalent cases (from 14,230.79 × 10^3 [95% UI: 12,125.99–16,328.89] to 14,830.58 × 10^3 [95% UI: 12,517.9–17,235.24]). The area with the lowest incidence rate was high-income North America (0.01 per 100,000 population), and the lowest prevalence rate was in Andean Latin America (93.66 per 100,000 population). Compared to 1990, the number of incident cases in South Asia increased by 57.00% in 2019. In terms of AAPC incidence, the region with the most significant decline was Latin America (AAPC = −4.56, *p* < 0.01). Notably, the South Asia showed an upward trend (AAPC = 0.77 [95% CI: 0.68–0.86]). However, regarding AAPC prevalence and DALYs, all regions worldwide showed a decline (Table 2).

## Discussion

To our knowledge, this is the first study to comprehensively analyze the incidence, prevalence, and DALYs of hepatitis B-associated cirrhosis in children and adolescents in different countries, regions, SDI, sex, and age groups worldwide. The change in disease burden and trends over 30 years can help us evaluate the effect of current prevention, epidemiological characteristics and further optimizing guidelines and facilitating the rational allocation of health resources. With increased global accessibility of the hepatitis B vaccine, the implementation of mother-infant blocking of hepatitis B, and the approval of hepatitis B antiviral drugs in adolescents and children, the occurrence of cirrhosis caused by hepatitis B has been reduced to a large extent objectively. This is consistent with our results, namely that the disease burden of hepatitis B-associated cirrhosis in children and adolescents has decreased globally.

Our study further demonstrated that the incidence and prevalence of HBV-associated cirrhosis in children and adolescents increased with age. GBD 2017 Cirrhosis Collaborators (11) reported that the number and incidence of hepatitis B-associated cirrhosis in males had always been higher than in females. Our research further found that in children and adolescents, males had a significantly higher incidence and prevalence of hepatitis B-associated cirrhosis than females; however, the sex differences in the incidence and prevalence of hepatitis B-associated cirrhosis in children and adolescents do not seem very apparent. The specific reasons must be addressed with large-scale prospective clinical cohort studies in children and adolescents.

The reduction of hepatitis B-associated cirrhosis in children and adolescents in China ranks among the top in the world, which is related to the significant increase in the coverage of the hepatitis B vaccine for infants in China. One survey also showed that the prevalence of hepatitis B surface antigens among children declined from approximately 10% in 1980s to less than 0.5% among children born after 2011 (18). Countries with increased AAPC incidence are mainly concentrated in Africa, and our results showed that the incidence of AAPC in Somalia increased significantly. Coincidentally, a study on hepatitis B prevention and control policies reported that Somalia had the lowest score for hepatitis B prevention and control policies (19). Notably, the incidence of AAPC in India (AAPC = 1.12, *p* < 0.01) has been increasing. India, a populous country, has had a low coverage rate of hepatitis B vaccination for social (20), religious, educational, and other reasons. By 2016, up to 45% of children aged 1–6 years in India had not received the hepatitis B vaccine (20). Hepatitis B vaccination was included in India’s national immunization plan for the first time in 2011. Improving the coverage of hepatitis B vaccination and active antiviral treatment for children and adolescents, monitoring the level of hepatitis B antibodies, and striving to reduce the occurrence of horizontal transmission is necessary. In addition, the incidence of AAPC in hepatitis B-associated cirrhosis has also increased slightly in Sweden, Finland, and Iceland. Although the World Health Organization recommended universal vaccination with the hepatitis B vaccine for children in 1995, Denmark, Finland, Iceland, Ireland, the Netherlands, Norway, Sweden, and the UK adopted a selective vaccination strategy for high-risk groups, which was controversial at that time (21, 22). These countries originally had a low prevalence of hepatitis B infection, which may be related to the influx of immigrants from other countries and regions. Our results further illustrate the necessity for timely hepatitis B vaccination in all children (23).

The age group of <5 years was not only the age group with the most significant decline in incidence but also the age group with the least number of cases. The World Health Organization set an important goal, requiring all countries to include the hepatitis B vaccine in their national routine infant immunization plans by 1997 (24), which may have played a key role in reducing hepatitis B-associated cirrhosis in children under 5 years. Previous epidemiological data show that the prevalence rate of hepatitis B-associated cirrhosis in children and adolescents gradually increases with age, and the <5 years age group has the lowest prevalence rate of hepatitis B (8). The current international guidelines on antiviral treatment of children with hepatitis B still maintain a relatively conservative attitude, especially the antiviral treatment of children in immune tolerance period is controversial, but research showed that a certain proportion of children in immune tolerance period have liver tissue damage (25). Our results show that at present, children with hepatitis B-associated cirrhosis are mainly concentrated in patients over 10 years old. In order to minimize the occurrence of hepatitis B-associated cirrhosis and other adverse liver outcomes at all ages, active antiviral treatment measures should be taken for children infected with HBV.

There is a correlation between SDI scores and disease burden (26). Our results show a decrease in hepatitis B-associated cirrhosis prevalence in all SDI regions, which is consistent with the findings of Zobair et al. (27). However, the incidence showed the different trend. Previous studies (28) have shown that reducing the mother-to-child transmission of hepatitis B and standardizing screening and diagnosis is difficult in low-income and middle-income countries, increasing the burden of prevention and treatment for children and adolescents in these areas. Our research showed that the incidence in low-SDI and low-middle SDI areas has increased, especially in low-SDI areas. In addition, it is interesting to note that the level of SDI in Central Asia is similar, but the incidence is significantly different, which may be caused by the difference in the medical level and prevalence of hepatitis B-associated cirrhosis in countries located in Central Asia. The patients with liver cirrhosis in Sub-Saharan Africa mainly have hepatitis B-associated cirrhosis, but for many reasons, it is often difficult to obtain timely and standardized diagnosis, treatment, and screening. Our results further showed that the Western Sahara region has the heaviest hepatitis B-associated cirrhosis burden. Previous studies have also demonstrated that hepatitis B-related mortality and incidence is very high in the Western Sahara region (8). In the future, it will be necessary to further expand the scope of hepatitis B screening in this area and improve the vaccination coverage and accessibility of hepatitis B antiviral drugs. From a regional perspective, the number of cases in Africa was relatively high in 1990 and 2019, which may be because the main transmission route of hepatitis B in Africa is horizontal transmission, poor health environment, low development, extremely low vaccination rate, and inadequate health education level (29).

It is noteworthy that the incidence in South Asia has increased significantly. We speculate that this trend is related to the late start of hepatitis B vaccination in South Asia, the low screening rate of hepatitis B, and the low public awareness. In addition, this trend could also be related to the local religion, culture, and poor health habits. As a densely populated region, the obstacles to eliminating hepatitis B are still widespread, which will continue to increase the disease burden of children and adolescents with hepatitis B-associated cirrhosis, and needs special attention. East Asia is the first region in the world to set control targets for hepatitis B prevention and focus on the control of hepatitis B prevalence among children and the blocking of maternal-to-infant transmission. Our results showed that the largest reduction in hepatitis B-related cirrhosis occurred in East Asia, which further illustrates their remarkable achievements in hepatitis B prevention and control strategies, and shows that maternal and infant blocking of hepatitis B plays a vital role in reducing the prevalence of hepatitis B-associated cirrhosis (30).

Our study has some limitations. First, owing to insufficient screening for liver cirrhosis in some countries and regions, the burden of liver cirrhosis may be underestimated. Second, although the GBD database is authoritative, some developing countries may not be able to fully report information, and there may be deviations between the actual situation and the calculated results. Third, because of data limitations, cirrhosis could not be divided into compensated and decompensated; therefore, it was impossible to further compare the differences. Finally, although we adopted various appropriate statistical methods to assess the disease burden and change trends, the quality of data reported by different countries and regions may be different and may not fully reflect the burden of cirrhosis.

## Conclusion

The global disease burden of hepatitis B-associated cirrhosis in children and adolescents has declined from 1990 to 2019. The number of patients in East Asia has decreased the most significantly; however, in low-income areas, especially in the Western Sahara region, the burden of hepatitis B-associated cirrhosis remains severe. In addition, the incidence of hepatitis B-associated cirrhosis in South Asia requires special attention. In the future, it will be necessary to further strengthen maternal and infant blocking of hepatitis B, antiviral treatment, and liver cirrhosis treatment in children with hepatitis B, especially in some African regions and low-income areas. At the same time, it is also necessary to regularly monitor and evaluate the incidence in South Asia and other countries and to take appropriate intervention measures.

## Data availability statement

The original contributions presented in the study are included in the article/Supplementary material, further inquiries can be directed to the corresponding author.

## Author contributions

CH: Formal analysis, Methodology, Software, Writing – original draft. YW: Data curation, Validation, Writing – review & editing. CZ: Writing – review & editing. DJ: Writing – review & editing. F-SW: Conceptualization, Resources, Writing – review & editing.

## Footnotes

^1^http://ghdx.healthdata.org

^2^http://ghdx.healthdata.org/gbd-results-tool
