# Supplementary material for: The burden of cirrhosis and other chronic liver diseases due to hepatitis B in children and adolescents: results from global burden of disease study 2019
Source: Front Public Health. 2023 Dec 22;11:1315392. doi: 10.3389/fpubh.2023.1315392 (PMC10766842; doi:10.3389/fpubh.2023.1315392)

Supplementary appendix to "Global, regional, and national burdens of hepatitis B-associated cirrhosis in children and adolescents from 1990 to 2019 and joinpoint regression"

1. Supplementary tables

1.1 Table S1: CoDEM and DisMod-MR covariates used for liver cirrhosis, GBD 2019……………2

1.2 Table S2**:** SDI values at the global, regional and national level………………………………….3

1.3 Table S3: Checklist of information that should be included in new reports of global health estimates……………………………………………………………………………………………9

1.4 Table S4: The joinpoint regression results of incidence in children and adolescents from 1990-2019 at regional level..10

1.5 Table S5: The joinpoint regression results of DALYs in children and adolescents from 1990-2019 at regional level..25

1.6 Table S6: The incidence of hepatitis B-associated cirrhosis in children and adolescents from 1990 to 2019 at the national and territorial levels..39

1.7 Table S7: The AAPC of Incidence, Prevalence and DALYs of hepatitis B-associated cirrhosis in children and adolescents from 1990 to 2019 at the national and territorial levels.............................47

1.8 Table S8: The prevalence of hepatitis B-associated cirrhosis in children and adolescents from 1990 to 2019 at the national and territorial levels..55

1.9 Table S9: The DALYs of hepatitis B-associated cirrhosis in children and adolescents from 1990 to 2019 at the national and territorial levels...64

2. Supplementary figures

2.1 Figure S1: Composition ratio of incidence of liver cirrhosis in different age groups at global and regional levels 72

2.2 Figure S2: Trends of incidence rate of liver cirrhosis in different age groups from 1990 to 2019……………………………………………………………………………………………73

2.3 Figure S3: Trends of incidence rate of liver cirrhosis in males of different age groups from 1990 to 2019..74

2.4 Figure S4: Trends of incidence rate of liver cirrhosis in females of different age groups from 1990 to 2019..75

2.5 Figure S5: Composition ratio of prevalence of liver cirrhosis in different age groups at global and regional levels…………………………………………………………………………......…76

2.6 Figure S6: Trends of prevalence rate of liver cirrhosis in different age groups from 1990 to 2019……………………………………………………………………………………………......77

2.7 Figure S7: Trends of prevalence rate of liver cirrhosis in males of different age groups from 1990 to 2019……………………………………………………………………………………………..78

2.8 Figure S8: Trends of prevalence rate of liver cirrhosis in females of different age groups from 1990 to 2019…………………………………………………………………………...………..79

Table S1. CoDEM and DisMod-MR covariates used for liver cancer and cirrhosis, GBD 2019

Cirrhosis	Cirrhosis due - HBV	Cirrhosis due - HCV	Cirrhosis due - ALD	Cirrhosis due -NAFLD/NASH
Alcohol (liters per capita)<br>Schistosomiasis prevalence<br>Hepatitis B prevalence<br>Hepatitis C prevalence<br>Log-transformed SEV scalar: Liver C<br>Hepatitis B3 vaccine (proportion covered)<br>Diabetes prevalence<br>Mean BMI<br>Healthcare access and quality index<br>Education (years per capita)<br>National income per capita ($ per capita)<br>Socio-demographic Index	Hepatitis B (HBsAg) seroprevalence<br>Proportion of liver cancer due - hepatitis B (age standardized)<br>Hepatitis B 3-dose coverage (proportion), lagged 10 years	Hepatitis C (IgG) seroprevalence<br>Proportion of liver cancer due - hepatitis C (age standardized)	Alcohol (liters per capita)<br>Proportion of liver cancer due - alcohol (age-standardized)<br>Alcohol abstainer proportion, age standardized	Mean BMI<br>Prevalence of obesity<br>Proportion of liver cancer due - NAFLD/NASH (age standardized)<br>NAFLD/NASH prevalence

Table S2. SDI values at the global, regional and national level

Location	SDI value	Classification
Global	0.65	/
**Australasia**	0.87	High SDI
Australia	0.87	High SDI
New Zealand	0.84	High SDI
**High-income North America**	0.87	High SDI
Canada	0.88	High SDI
United States	0.87	High SDI
Greenland	0.76	High-middle SDI
**High-income Asia Pacific**	0.87	High SDI
Brunei	0.86	High SDI
Japan	0.87	High SDI
South Korea	0.87	High SDI
Singapore	0.87	High SDI
**Southern Latin America**	0.72	High-middle SDI
Argentina	0.71	High-middle SDI
Chile	0.75	High-middle SDI
Uruguay	0.71	High-middle SDI
**Western Europe**	0.86	High SDI
Andorra	0.9	High SDI
Austria	0.87	High SDI
Belgium	0.89	High SDI
Cyprus	0.86	High SDI
Denmark	0.92	High SDI
Finland	0.89	High SDI
France	0.86	High SDI
Germany	0.87	High SDI
Greece	0.82	High SDI
Iceland	0.91	High SDI
Ireland	0.88	High SDI
Israel	0.82	High SDI
Italy	0.84	High SDI
Luxembourg	0.92	High SDI
Malta	0.84	High SDI
Netherlands	0.91	High SDI
Norway	0.91	High SDI
Portugal	0.78	High-middle SDI
Spain	0.82	High SDI
Sweden	0.88	High SDI
Switzerland	0.89	High SDI

United Kingdom	0.84	High SDI
**Central Europe**	0.81	High SDI
Albania	0.68	Middle SDI
Bosnia and Herzegovina	0.71	High-middle SDI
Bulgaria	0.79	High-middle SDI
Croatia	0.82	High SDI
Czech Republic	0.85	High SDI
Hungary	0.82	High SDI
Macedonia	0.75	High-middle SDI
Montenegro	0.79	High-middle SDI
Poland	0.84	High SDI
Romania	0.78	High-middle SDI
Serbia	0.75	High-middle SDI
Slovakia	0.84	High SDI
Slovenia	0.86	High SDI
**Eastern Europe**	0.79	High-middle SDI
Belarus	0.77	High-middle SDI
Estonia	0.86	High SDI
Latvia	0.83	High SDI
Lithuania	0.84	High SDI
Moldova	0.68	Middle SDI
Russian Federation	0.79	High-middle SDI
Ukraine	0.74	High-middle SDI
**Central Asia**	0.67	Middle SDI
Armenia	0.7	High-middle SDI
Azerbaijan	0.7	High-middle SDI
Georgia	0.7	High-middle SDI
Kazakhstan	0.74	High-middle SDI
Kyrgyzstan	0.61	Middle SDI
Mongolia	0.66	Middle SDI
Tajikistan	0.52	Low-middle SDI
Turkmenistan	0.7	High-middle SDI
Uzbekistan	0.63	Middle SDI
**Southeast Asia**	0.64	Middle SDI
Cambodia	0.48	Low-middle SDI
Indonesia	0.65	Middle SDI
Laos	0.52	Low-middle SDI
Malaysia	0.76	High-middle SDI
Maldives	0.66	Middle SDI
Myanmar	0.56	Low-middle SDI
Philippines	0.62	Middle SDI

Sri Lanka	0.68	Middle SDI
Thailand	0.68	Middle SDI
Timor-Leste	0.5	Low-middle SDI
Vietnam	0.61	Middle SDI
Mauritius	0.72	High-middle SDI
Seychelles	0.69	High-middle SDI
**East Asia**	0.71	High-middle SDI
China	0.71	High-middle SDI
North Korea	0.54	Low-middle SDI
Taiwan	0.86	High SDI
**Oceania**	0.47	Low-middle SDI
Fiji	0.64	Middle SDI
Kiribati	0.43	Low SDI
Marshall Islands	0.55	Low-middle SDI
Federated States of Micronesia	0.58	Low-middle SDI
Papua New Guinea	0.42	Low SDI
Samoa	0.58	Low-middle SDI
Solomon Islands	0.43	Low SDI
Tonga	0.62	Middle SDI
Vanuatu	0.48	Low-middle SDI
American Samoa	0.7	High-middle SDI
Guam	0.79	High-middle SDI
Northern Mariana Islands	0.76	High-middle SDI
**South Asia**	0.53	Low-middle SDI
Bangladesh	0.46	Low-middle SDI
Bhutan	0.57	Low-middle SDI
India	0.55	Low-middle SDI
Nepal	0.43	Low SDI
Pakistan	0.49	Low-middle SDI
**Andean Latin America**	0.63	Middle SDI
Bolivia	0.59	Low-middle SDI
Ecuador	0.64	Middle SDI
Peru	0.64	Middle SDI
**Caribbean**	0.64	Middle SDI
Antigua and Barbuda	0.72	High-middle SDI
The Bahamas	0.76	High-middle SDI
Barbados	0.74	High-middle SDI
Belize	0.6	Low-middle SDI
Cuba	0.69	High-middle SDI
Dominica	0.69	High-middle SDI
Dominican Republic	0.59	Low-middle SDI

Grenada	0.64	Middle SDI
Guyana	0.58	Low-middle SDI
Haiti	0.44	Low SDI
Jamaica	0.68	Middle SDI
Saint Lucia	0.65	Middle SDI
Saint Vincent and the Grenadines	0.61	Middle SDI
Suriname	0.64	Middle SDI
Trinidad and Tobago	0.7	High-middle SDI
Bermuda	0.81	High SDI
Puerto Rico	0.81	High SDI
Virgin Islands, U.S.	0.81	High SDI
**Central Latin America**	0.62	Middle SDI
Colombia	0.63	Middle SDI
Costa Rica	0.66	Middle SDI
El Salvador	0.59	Low-middle SDI
Guatemala	0.52	Low-middle SDI
Honduras	0.51	Low-middle SDI
Mexico	0.63	Middle SDI
Nicaragua	0.53	Low-middle SDI
Panama	0.68	Middle SDI
Venezuela	0.66	Middle SDI
**Tropical Latin America**	0.66	Middle SDI
Brazil	0.66	Middle SDI
Paraguay	0.62	Middle SDI
**North Africa and Middle East**	0.64	Middle SDI
Algeria	0.7	High-middle SDI
Bahrain	0.71	High-middle SDI
Egypt	0.6	Low-middle SDI
Iran	0.7	High-middle SDI
Iraq	0.58	Low-middle SDI
Jordan	0.7	High-middle SDI
Kuwait	0.79	High-middle SDI
Lebanon	0.73	High-middle SDI
Libya	0.76	High-middle SDI
Morocco	0.58	Low-middle SDI
Palestine	0.54	Low-middle SDI
Oman	0.74	High-middle SDI
Qatar	0.77	High-middle SDI
Saudi Arabia	0.78	High-middle SDI
Syria	0.61	Middle SDI
Tunisia	0.68	Middle SDI

Turkey	0.73	High-middle SDI
United Arab Emirates	0.79	High-middle SDI
Yemen	0.43	Low SDI
Afghanistan	0.29	Low SDI
Sudan	0.48	Low-middle SDI
**Central Sub-Saharan Africa**	0.46	Low-middle SDI
Angola	0.46	Low-middle SDI
Central African Republic	0.33	Low SDI
Congo	0.57	Low-middle SDI
Democratic Republic of the Congo	0.36	Low SDI
Equatorial Guinea	0.63	Middle SDI
Gabon	0.65	Middle SDI
**Eastern Sub-Saharan Africa**	0.39	Low SDI
Burundi	0.31	Low SDI
Comoros	0.43	Low SDI
Djibouti	0.48	Low-middle SDI
Eritrea	0.41	Low SDI
Ethiopia	0.33	Low SDI
Kenya	0.5	Low-middle SDI
Madagascar	0.33	Low SDI
Malawi	0.35	Low SDI
Mozambique	0.34	Low SDI
Rwanda	0.41	Low SDI
Somalia	0.23	Low SDI
Tanzania	0.41	Low SDI
Uganda	0.39	Low SDI
Zambia	0.47	Low-middle SDI
South Sudan	0.27	Low SDI
**Southern Sub-Saharan Africa**	0.64	Middle SDI
Botswana	0.66	Middle SDI
Lesotho	0.49	Low-middle SDI
Namibia	0.62	Middle SDI
South Africa	0.68	Middle SDI
Swaziland	0.58	Low-middle SDI
Zimbabwe	0.46	Low-middle SDI
**Western Sub-Saharan Africa**	0.44	Low SDI
Benin	0.37	Low SDI
Burkina Faso	0.28	Low SDI
Cameroon	0.48	Low-middle SDI
Cape Verde	0.55	Low-middle SDI
Chad	0.25	Low SDI

Cote dIvoire	0.41	Low SDI
The Gambia	0.4	Low SDI
Ghana	0.54	Low-middle SDI
Guinea	0.32	Low SDI
Guinea-Bissau	0.35	Low SDI
Liberia	0.33	Low SDI
Mali	0.27	Low SDI
Mauritania	0.47	Low-middle SDI
Niger	0.19	Low SDI
Nigeria	0.49	Low-middle SDI
Sao Tome and Principe	0.49	Low-middle SDI
Senegal	0.37	Low SDI
Sierra Leone	0.36	Low SDI
Togo	0.41	Low SDI

SDI, socio-demographic index.

Table S3: Checklist of information that should be included in new reports of global health estimates

Item #	Checklist item	Reported on page #
**Objectives and funding**		
**1**	Define the indicator(s), populations (including age, sex, and geographic entities), and time period(s) for which estimates were made.	Page 5
**2**	List the funding sources for the work.	Page 3
**Data Inputs**		
For all data inputs from multiple sources that are synthesized as part of the study:		
**3**	Describe how the data were identified and how the data were accessed.	Page 4-5
**4**	Specify the inclusion and exclusion criteria. Identify all ad-hoc exclusions.	Page 4-5
**5**	Provide information on all included data sources and their main characteristics. For each data source used, report reference information or contact name/institution, population represented, data collection method, year(s) of data collection, sex and age range, diagnostic criteria or measurement method, and	http://ghdx.healthdata.org/gbd-2019/data-input-sources
**6**	Identify and describe any categories of input data that have potentially important biases (e.g., based on characteristics listed in item 5).	Page 13
For data inputs that contribute to the analysis but were not synthesized as part of the study:		
**7**	Describe and give sources for any other data inputs.	Page 4-5
For all data inputs:		
**8**	Provide all data inputs in a file format from which data can be efficiently extracted (e.g., a spreadsheet rather than a PDF), including all relevant meta-data listed in item 5. For any data inputs that cannot be shared because of ethical or legal reasons, such as third-party ownership, provide a contact name or the name of the institution that retains the right to the data.	Online data tools http://ghdx.healthdata.org/; http://ghdx.healthdata.org/gbd-data-tool
**Data analysis**		
**9**	Provide a conceptual overview of the data analysis method. A diagram may be helpful.	Page 4-5
**10**	Provide a detailed description of all steps of the analysis, including mathematical formulae. This description should cover, as relevant, data cleaning, data pre-processing, data adjustments and weighting of data sources, and mathematical or statistical model(s).	Page 4-5; tableS1
**11**	Describe how candidate models were evaluated and how the final model(s) were selected.	Page 4-5; tableS1
**12**	Provide the results of an evaluation of model performance, if done, as well as the results of any relevant sensitivity analysis.	no
**13**	Describe methods for calculating uncertainty of the estimates. State which sources of uncertainty were, and were not, accounted for in the uncertainty analysis.	Page 5
**14**	State how analytic or statistical source code used to generate estimates can be accessed.	This will be available in an online repository that will be released
**Results and Discussion**		
**15**	Provide published estimates in a file format from which data can be efficiently extracted.	http://ghdx.healthdata.org/;
**16**	Report a quantitative measure of the uncertainty of the estimates (e.g. uncertainty intervals).	http://ghdx.healthdata.org/;
**17**	Interpret results in light of existing evidence. If updating a previous set of estimates, describe the reasons for changes in estimates.	Research in Context
**18**	Discuss limitations of the estimates. Include a discussion of any modelling assumptions or data limitations that affect interpretation of the estimates.	Page 13

Supplementary Table 4: The joinpoint regression results of incidence in children and adolescents from 1990-2019 at regional level

Location	Sex	Joinpoint	Segment	Segment.Start	Segment.End	Significant_indicator	Test.Statistic	P value	APC_95CI
Andean Latin America	Both	4	0	1990	1998	1	43.5597	0	1.79(1.71, 1.88)
Andean Latin America	Both	4	1	1998	2003	1	6.8363	0	0.81(0.56, 1.06)
Andean Latin America	Both	4	2	2003	2006	1	-3.6571	0.002	-1.4(-2.2, -0.59)
Andean Latin America	Both	4	3	2006	2010	1	-41.9293	0	-8.12(-8.52, -7.73)
Andean Latin America	Both	4	4	2010	2019	1	-72.8981	0	-2.71(-2.79, -2.63)
Andean Latin America	Female	4	0	1990	1996	1	20.5034	0	1.48(1.33, 1.64)
Andean Latin America	Female	4	1	1996	2003	0	1.0646	0.303	0.08(-0.08, 0.23)
Andean Latin America	Female	4	2	2003	2006	1	-4.4161	0	-1.98(-2.91, -1.03)
Andean Latin America	Female	4	3	2006	2010	1	-39.8649	0	-8.61(-9.04, -8.17)
Andean Latin America	Female	4	4	2010	2019	1	-55.5766	0	-2.33(-2.42, -2.25)
Andean Latin America	Male	4	0	1990	1999	1	52.2398	0	1.88(1.8, 1.96)
Andean Latin America	Male	4	1	1999	2003	1	4.4089	0	0.85(0.44, 1.26)
Andean Latin America	Male	4	2	2003	2006	1	-3.1983	0.006	-1.28(-2.12, -0.43)
Andean Latin America	Male	4	3	2006	2010	1	-40.4509	0	-8.17(-8.58, -7.76)
Andean Latin America	Male	4	4	2010	2019	1	-73.4228	0	-2.84(-2.92, -2.76)
Caribbean	Both	4	0	1990	1996	1	-6.1653	0	-0.51(-0.68, -0.33)
Caribbean	Both	4	1	1996	1999	1	-4.3936	0	-2.09(-3.08, -1.08)
Caribbean	Both	4	2	1999	2010	1	-28.8042	0	-1.11(-1.19, -1.03)
Caribbean	Both	4	3	2010	2015	1	-13.3713	0	-2.11(-2.44, -1.78)
Caribbean	Both	4	4	2015	2019	1	18.1646	0	2.97 (2.62, 3.33)
Caribbean	Female	5	0	1990	1996	0	-2.0081	0.066	-0.08(-0.17, 0.01)
Caribbean	Female	5	1	1996	1999	1	-12.2571	0	-2.9(-3.4, -2.39)
Caribbean	Female	5	2	1999	2005	1	-32.9675	0	-1.78(-1.9, -1.66)

Caribbean	Female	5	3	2005	2010	1	-7.3241	0	-0.57(-0.74, -0.4)
Caribbean	Female	5	4	2010	2015	1	-32.3237	0	-2.45(-2.61, -2.29)
Caribbean	Female	5	5	2015	2019	1	21.3887	0	1.69(1.52, 1.87)
Caribbean	Male	4	0	1990	1996	1	-8.4359	0	-0.67(-0.84, -0.5)
Caribbean	Male	4	1	1996	1999	1	-3.9116	0.001	-1.8(-2.75, -0.83)
Caribbean	Male	4	2	1999	2009	1	-23.8917	0	-1.05(-1.14, -0.96)
Caribbean	Male	4	3	2009	2015	1	-18.2249	0	-1.97(-2.2, -1.75)
Caribbean	Male	4	4	2015	2019	1	20.602	0	3.31(2.96, 3.66)
Central Asia	Both	5	0	1990	1998	1	51.8831	0	5.19(4.96, 5.41)
Central Asia	Both	5	1	1998	2004	1	16.7377	0	3.47(3.01, 3.92)
Central Asia	Both	5	2	2004	2007	0	-0.7838	0.447	-0.72(-2.68, 1.28)
Central Asia	Both	5	3	2007	2011	1	-12.0058	0	-5.42(-6.36, -4.47)
Central Asia	Both	5	4	2011	2016	1	-41.7186	0	-12.07(-12.65, -11.48)
Central Asia	Both	5	5	2016	2019	1	-4.2985	0.001	-2.19(-3.27, -1.09)
Central Asia	Female	4	0	1990	2000	1	121.3309	0	4.34(4.26, 4.42)
Central Asia	Female	4	1	2000	2006	1	9.1292	0	0.95(0.73, 1.18)
Central Asia	Female	4	2	2006	2011	1	-41.2682	0	-5.81(-6.1, -5.52)
Central Asia	Female	4	3	2011	2016	1	-71.6037	0	-10.17(-10.45, -9.88)
Central Asia	Female	4	4	2016	2019	1	-15.1793	0	-3.79(-4.31, -3.27)
Central Asia	Male	5	0	1990	1995	1	35.434	0	6.09(5.71, 6.48)
Central Asia	Male	5	1	1995	2004	1	49.84	0	4.16(3.98, 4.34)
Central Asia	Male	5	2	2004	2007	0	-0.8986	0.385	-0.69(-2.34, 0.98)
Central Asia	Male	5	3	2007	2011	1	-13.9572	0	-5.24(-6.03, -4.45)
Central Asia	Male	5	4	2011	2016	1	-52.3855	0	-12.71(-13.2, -12.22)
Central Asia	Male	5	5	2016	2019	1	-4.0182	0.001	-1.7(-2.6, -0.79)
Central Europe	Both	4	0	1990	2000	1	13.243	0	1.24(1.04, 1.44)

Central Europe	Both	4	1	2000	2006	1	-14.5126	0	-3.81(-4.35, -3.26)
Central Europe	Both	4	2	2006	2009	1	-5.9832	0	-7.1(-9.49, -4.64)
Central Europe	Both	4	3	2009	2014	1	-7.7158	0	-2.99(-3.8, -2.18)
Central Europe	Both	4	4	2014	2019	0	-0.0669	0.948	-0.02(-0.6, 0.57)
Central Europe	Female	5	0	1990	1995	0	-1.1614	0.266	-0.32(-0.91, 0.28)
Central Europe	Female	5	1	1995	2000	1	3.8364	0.002	1.51(0.66, 2.38)
Central Europe	Female	5	2	2000	2006	1	-15.1067	0	-4.06(-4.63, -3.49)
Central Europe	Female	5	3	2006	2009	1	-5.7216	0	-6.88(-9.36, -4.34)
Central Europe	Female	5	4	2009	2014	1	-6.1348	0	-2.48(-3.33, -1.61)
Central Europe	Female	5	5	2014	2019	0	0.6931	0.5	0.2(-0.41, 0.81)
Central Europe	Male	4	0	1990	2000	1	16.4039	0	1.43(1.24, 1.62)
Central Europe	Male	4	1	2000	2006	1	-15.8213	0	-3.87(-4.38, -3.36)
Central Europe	Male	4	2	2006	2009	1	-6.432	0	-7.14(-9.37, -4.84)
Central Europe	Male	4	3	2009	2014	1	-8.9514	0	-3.2(-3.94, -2.45)
Central Europe	Male	4	4	2014	2019	0	-0.4062	0.69	-0.11(-0.65, 0.45)
Central Latin America	Both	5	0	1990	1994	1	-31.4029	0	-5.06(-5.4, -4.72)
Central Latin America	Both	5	1	1994	1999	1	-18.8042	0	-3.17(-3.52, -2.81)
Central Latin America	Both	5	2	1999	2006	1	-23.6793	0	-2.26(-2.47, -2.06)
Central Latin America	Both	5	3	2006	2009	1	-10.3271	0	-6.05(-7.27, -4.82)
Central Latin America	Both	5	4	2009	2016	1	-20.3136	0	-2.15(-2.37, -1.92)
Central Latin America	Both	5	5	2016	2019	1	9.525	0	3.13(2.42, 3.86)
Central Latin America	Female	5	0	1990	1994	1	-89.9051	0	-7.96(-8.14, -7.77)
Central Latin America	Female	5	1	1994	1999	1	-40.8804	0	-3.95(-4.16, -3.75)
Central Latin America	Female	5	2	1999	2006	1	-23.1831	0	-1.27(-1.39, -1.16)
Central Latin America	Female	5	3	2006	2009	1	-18.6887	0	-6.19(-6.88, -5.5)
Central Latin America	Female	5	4	2009	2017	1	-52.3994	0	-2.57(-2.67, -2.46)

Central Latin America	Female	5	5	2017	2019	1	13.184	0	5.01(4.17, 5.85)
Central Latin America	Male	5	0	1990	1994	1	-27.5544	0	-4.62(-4.98, -4.27)
Central Latin America	Male	5	1	1994	1998	1	-11.8366	0	-3.21(-3.78, -2.63)
Central Latin America	Male	5	2	1998	2006	1	-32.8788	0	-2.49(-2.65, -2.32)
Central Latin America	Male	5	3	2006	2009	1	-10.1712	0	-6.08(-7.32, -4.82)
Central Latin America	Male	5	4	2009	2016	1	-19.2244	0	-2.09(-2.32, -1.85)
Central Latin America	Male	5	5	2016	2019	1	9.8111	0	3.25(2.53, 3.98)
Central Sub-Saharan Africa	Both	1	0	1990	2014	1	6.1048	0	0.46(0.3, 0.61)
Central Sub-Saharan Africa	Both	1	1	2014	2019	1	-8.34	0	-6.67(-8.25, -5.07)
Central Sub-Saharan Africa	Female	2	0	1990	2005	1	3.9939	0.001	0.47(0.23, 0.71)
Central Sub-Saharan Africa	Female	2	1	2005	2015	1	-4.9453	0	-1.25(-1.76, -0.73)
Central Sub-Saharan Africa	Female	2	2	2015	2019	1	-9.5748	0	-8.18(-9.86, -6.46)
Central Sub-Saharan Africa	Male	1	0	1990	2014	1	9.0726	0	0.63(0.49, 0.78)
Central Sub-Saharan Africa	Male	1	1	2014	2019	1	-8.402	0	-6.33(-7.82, -4.82)
East Asia	Both	4	0	1990	1996	1	-12.4257	0	-2.13(-2.49, -1.77)
East Asia	Both	4	1	1996	2006	1	11.9268	0	1.13(0.93, 1.33)
East Asia	Both	4	2	2006	2011	1	-31.0138	0	-10.22(-10.87, -9.55)
East Asia	Both	4	3	2011	2014	1	-15.7504	0	-16.49(-18.49, -14.44)
East Asia	Both	4	4	2014	2019	1	-13.1743	0	-3.34(-3.86, -2.81)
East Asia	Female	3	0	1990	1995	1	-6.2857	0	-2.13(-2.82, -1.42)
East Asia	Female	3	1	1995	2006	1	2.5376	0.02	0.31(0.05, 0.56)
East Asia	Female	3	2	2006	2015	1	-71.55	0	-12.97(-13.32, -12.61)
East Asia	Female	3	3	2015	2019	1	-3.5294	0.002	-2.01(-3.19, -0.82)
East Asia	Male	4	0	1990	1996	1	-12.4347	0	-2.37(-2.77, -1.97)
East Asia	Male	4	1	1996	2006	1	12.0298	0	1.3(1.07, 1.53)
East Asia	Male	4	2	2006	2010	1	-16.8761	0	-9.49(-10.62, -8.35)

East Asia	Male	4	3	2010	2015	1	-38.1504	0	-13.89(-14.6, -13.17)
East Asia	Male	4	4	2015	2019	1	-3.977	0.001	-1.58(-2.41, -0.74)
Eastern Europe	Both	5	0	1990	1995	1	11.8268	0	2.39(1.95, 2.84)
Eastern Europe	Both	5	1	1995	2000	1	28.9502	0	8.77(8.09, 9.45)
Eastern Europe	Both	5	2	2000	2005	1	10.0323	0	3.08(2.41, 3.76)
Eastern Europe	Both	5	3	2005	2010	1	-15.8305	0	-4.71(-5.34, -4.08)
Eastern Europe	Both	5	4	2010	2015	1	-47.1056	0	-13.1(-13.66, -12.54)
Eastern Europe	Both	5	5	2015	2019	1	5.9074	0	1.77(1.12, 2.42)
Eastern Europe	Female	5	0	1990	1995	1	9.4994	0	1.69(1.31, 2.08)
Eastern Europe	Female	5	1	1995	2000	1	32.2824	0	8.87(8.25, 9.49)
Eastern Europe	Female	5	2	2000	2005	1	8.9357	0	2.47(1.87, 3.08)
Eastern Europe	Female	5	3	2005	2010	1	-13.8127	0	-3.66(-4.22, -3.09)
Eastern Europe	Female	5	4	2010	2015	1	-48.6821	0	-12.23(-12.73, -11.72)
Eastern Europe	Female	5	5	2015	2019	1	8.0292	0	2.19(1.59, 2.78)
Eastern Europe	Male	5	0	1990	1995	1	12.478	0	2.62(2.16, 3.08)
Eastern Europe	Male	5	1	1995	2000	1	27.5229	0	8.71(8,23, 9.42)
Eastern Europe	Male	5	2	2000	2005	1	10.0854	0	3.25(2.54, 3.96)
Eastern Europe	Male	5	3	2005	2010	1	-16.427	0	-5.08(-5.73, -4.43)
Eastern Europe	Male	5	4	2010	2015	1	-46.9434	0	-13.42(-13.99, -12.84)
Eastern Europe	Male	5	5	2015	2019	1	5.0869	0	1.6(0.92, 2.29)
Eastern Sub-Saharan Africa	Both	4	0	1990	2007	1	60.1595	0	0.33(0.32, 0.34)
Eastern Sub-Saharan Africa	Both	4	1	2007	2011	1	-2.4751	0.025	-0.2(-0.36, -0.03)
Eastern Sub-Saharan Africa	Both	4	2	2011	2014	1	-12.9798	0	-2(-2.32, -1.68)
Eastern Sub-Saharan Africa	Both	4	3	2014	2017	1	-9.0218	0	-1.39(-1.71, -1.06)
Eastern Sub-Saharan Africa	Both	4	4	2017	2019	0	-0.4566	0.654	-0.07(-0.41, 0.27)
Eastern Sub-Saharan Africa	Female	5	0	1990	1993	0	0.7499	0.467	0.16(-0.3, 0.61)

Eastern Sub-Saharan Africa	Female	5	1	1993	2006	1	-21.7146	0	-0.52(-0.57, -0.47)
Eastern Sub-Saharan Africa	Female	5	2	2006	2011	1	-11.3741	0	-1.47(-1.74, -1.19)
Eastern Sub-Saharan Africa	Female	5	3	2011	2014	1	-7.2793	0	-2.91(-3.76, -2.06)
Eastern Sub-Saharan Africa	Female	5	4	2014	2017	1	-3.0209	0.01	-1.24(-2.12, -0.36)
Eastern Sub-Saharan Africa	Female	5	5	2017	2019	0	-0.6565	0.523	-0.28(-1.19, 0.64)
Eastern Sub-Saharan Africa	Male	5	0	1990	1996	1	12.9261	0	0.52(0.44, 0.61)
Eastern Sub-Saharan Africa	Male	5	1	1996	1999	1	3.6399	0.003	0.86(0.35, 1.37)
Eastern Sub-Saharan Africa	Male	5	2	1999	2008	1	23.4772	0	0.61(0.55, 0.66)
Eastern Sub-Saharan Africa	Male	5	3	2008	2011	0	0.6312	0.539	0.15(-0.36, 0.66)
Eastern Sub-Saharan Africa	Male	5	4	2011	2017	1	-30.7957	0	-1.59(-1.7, -1.48)
Eastern Sub-Saharan Africa	Male	5	5	2017	2019	0	0.3369	0.742	0.08(-0.44, 0.6)
Global	Both	4	0	1990	1995	1	-2.7502	0.014	-0.26(-0.46, -0.06)
Global	Both	4	1	1995	2002	1	18.0776	0	1.31(1.15, 1.46)
Global	Both	4	2	2002	2006	0	1.4036	0.18	0.3(-0.15, 0.75)
Global	Both	4	3	2006	2014	1	-29.1978	0	-1.63(-1.74, -1.51)
Global	Both	4	4	2014	2019	1	-11.5519	0	-1.1(-1.3, -0.9)
Global	Female	5	0	1990	1994	1	-7.2033	0	-0.72(-0.93, -0.5)
Global	Female	5	1	1994	2002	1	22.7368	0	0.95(0.86, 1.04)
Global	Female	5	2	2002	2006	0	1.817	0.092	0.28(-0.05, 0.61)
Global	Female	5	3	2006	2011	1	-10.8413	0	-1.04(-1.25, -0.84)
Global	Female	5	4	2011	2014	1	-7.9458	0	-2.39(-3.03, -1.75)
Global	Female	5	5	2014	2019	1	-6.9258	0	-0.48(-0.63, -0.33)
Global	Male	4	0	1990	1995	1	-2.2489	0.039	-0.21(-0.4, -0.01)
Global	Male	4	1	1995	2003	1	23.7194	0	1.31(1.19, 1.42)
Global	Male	4	2	2003	2006	0	0.3218	0.752	0.13(-0.75, 1.02)
Global	Male	4	3	2006	2009	1	-5.0321	0	-2.05(-2.91, -1.19)

Global	Male	4	4	2009	2019	1	-43.2501	0	-1.4(-1.46, -1.33)
High SDI	Both	5	0	1990	1994	1	-85.4675	0	-6.85(-7.02, -6.68)
High SDI	Both	5	1	1994	2000	1	-59.2311	0	-3.5(-3.63, -3.38)
High SDI	Both	5	2	2000	2004	1	-31.4704	0	-4.14(-4.42, -3.86)
High SDI	Both	5	3	2004	2011	1	-58.2205	0	-2.71(-2.81, -2.61)
High SDI	Both	5	4	2011	2014	1	-13.1086	0	-3.63(-4.22, -3.04)
High SDI	Both	5	5	2014	2019	1	-14.3648	0	-0.92(-1.06, -0.78)
High SDI	Female	4	0	1990	1994	1	-42.8507	0	-8.68(-9.09, -8.27)
High SDI	Female	4	1	1994	2001	1	-25.0338	0	-2.86(-3.1, -2.62)
High SDI	Female	4	2	2001	2004	1	-5.9699	0	-4.01(-5.39, -2.6)
High SDI	Female	4	3	2004	2014	1	-34.6443	0	-2.21(-2.34, -2.08)
High SDI	Female	4	4	2014	2019	1	-4.4784	0	-0.71(-1.04, -0.37)
High SDI	Male	4	0	1990	1993	1	-22.7091	0	-6.26(-6.83, -5.7)
High SDI	Male	4	1	1993	2004	1	-93.0833	0	-4.11(-4.2, -4.02)
High SDI	Male	4	2	2004	2010	1	-24.2047	0	-3.1(-3.36, -2.83)
High SDI	Male	4	3	2010	2015	1	-19.704	0	-3.68(-4.06, -3.29)
High SDI	Male	4	4	2015	2019	1	-3.4619	0.003	-0.69(-1.11, -0.27)
High-middle SDI	Both	4	0	1990	1994	0	-1.4283	0.172	-0.37(-0.92, 0.18)
High-middle SDI	Both	4	1	1994	2003	1	16.0663	0	1.47(1.28, 1.67)
High-middle SDI	Both	4	2	2003	2006	0	-0.8159	0.427	-0.69(-2.45, 1.11)
High-middle SDI	Both	4	3	2006	2015	1	-86.8036	0	-7.72(-7.9, -7.54)
High-middle SDI	Both	4	4	2015	2019	1	5.0973	0	1.39(0.81, 1.97)
High-middle SDI	Female	4	0	1990	1994	1	-2.4754	0.025	-0.64(-1.19, -0.09)
High-middle SDI	Female	4	1	1994	2003	1	13.1711	0	1.22(1.02, 1.42)
High-middle SDI	Female	4	2	2003	2006	0	-1.1516	0.266	-0.98(-2.75, 0.83)
High-middle SDI	Female	4	3	2006	2014	1	-73.7494	0	-8.02(-8.24, -7.8)

High-middle SDI	Female	4	4	2014	2019	1	-2.3816	0.03	-0.47(-0.88, -0.05)
High-middle SDI	Male	4	0	1990	1994	0	-1.0053	0.33	-0.27(-0.85, 0.3)
High-middle SDI	Male	4	1	1994	2003	1	15.7067	0	1.51(1.31, 1.72)
High-middle SDI	Male	4	2	2003	2006	0	-0.6107	0.55	-0.53(-2.36, 1.33)
High-middle SDI	Male	4	3	2006	2015	1	-84.0054	0	-7.81(-8, -7.62)
High-middle SDI	Male	4	4	2015	2019	1	5.0956	0	1.46(0.85, 2.08)
Low SDI	Both	4	0	1990	1996	0	0.6453	0.528	0.03(-0.07, 0.13)
Low SDI	Both	4	1	1996	2005	1	30.1271	0	0.92(0.85, 0.98)
Low SDI	Both	4	2	2005	2011	0	-2.0257	0.06	-0.13(-0.26, 0.01)
Low SDI	Both	4	3	2011	2015	1	-4.8754	0	-0.68(-0.98, -0.39)
Low SDI	Both	4	4	2015	2019	1	-21.2308	0	-1.94(-2.13, -1.75)
Low SDI	Female	3	0	1990	1996	0	-1.0236	0.319	-0.06(-0.19, 0.06)
Low SDI	Female	3	1	1996	2005	1	15.8013	0	0.6(0.52, 0.69)
Low SDI	Female	3	2	2005	2010	0	-1.8412	0.081	-0.2(-0.43, 0.03)
Low SDI	Female	3	3	2010	2019	1	-47.0629	0	-1.53(-1.6, -1.46)
Low SDI	Male	3	0	1990	1996	0	1.205	0.243	0.07(-0.05, 0.18)
Low SDI	Male	3	1	1996	2005	1	30.8751	0	1.08(1.01, 1.16)
Low SDI	Male	3	2	2005	2015	0	-1.6362	0.118	-0.05(-0.11, 0.01)
Low SDI	Male	3	3	2015	2019	1	-20.846	0	-2.18(-2.4, -1.97)
Low-middle SDI	Both	3	0	1990	1995	0	-0.8877	0.386	-0.11(-0.36, 0.15)
Low-middle SDI	Both	3	1	1995	2004	1	26.846	0	1.59(1.47, 1.72)
Low-middle SDI	Both	3	2	2004	2011	0	1.8201	0.085	0.17(-0.03, 0.37)
Low-middle SDI	Both	3	3	2011	2019	1	-23.2556	0	-1.42(-1.55, -1.3)
Low-middle SDI	Female	5	0	1990	1995	0	-1.5599	0.143	-0.1(-0.24, 0.04)
Low-middle SDI	Female	5	1	1995	2005	1	51.5753	0	1.39(1.33, 1.45)
Low-middle SDI	Female	5	2	2005	2011	1	10.2508	0	0.68(0.54, 0.83)

Low-middle SDI	Female	5	3	2011	2014	1	-8.9512	0	-2.63(-3.25, -2)
Low-middle SDI	Female	5	4	2014	2017	1	-2.4792	0.028	-0.75(-1.39, -0.1)
Low-middle SDI	Female	5	5	2017	2019	0	1.9106	0.078	0.58(-0.08, 1.25)
Low-middle SDI	Male	3	0	1990	1995	0	-1.4074	0.175	-0.19(-0.48, 0.09)
Low-middle SDI	Male	3	1	1995	2004	1	24.3678	0	1.65(1.51, 1.79)
Low-middle SDI	Male	3	2	2004	2013	0	-1.4687	0.158	-0.1(-0.24, 0.04)
Low-middle SDI	Male	3	3	2013	2019	1	-17.4283	0	-1.85(-2.07, -1.63)
Middle SDI	Both	5	0	1990	1995	0	-2.1254	0.053	-0.17(-0.35, 0)
Middle SDI	Both	5	1	1995	2001	1	13.5207	0	1.1(0.93, 1.28)
Middle SDI	Both	5	2	2001	2006	1	-5.1736	0	-0.61(-0.86, -0.35)
Middle SDI	Both	5	3	2006	2009	1	-12.9643	0	-4.7(-5.46, -3.93)
Middle SDI	Both	5	4	2009	2014	1	-33.0297	0	-3.77(-4.01, -3.53)
Middle SDI	Both	5	5	2014	2019	1	-10.8332	0	-0.89(-1.07, -0.72)
Middle SDI	Female	5	0	1990	1994	1	-2.5437	0.024	-0.41(-0.75, -0.06)
Middle SDI	Female	5	1	1994	2001	1	5.5526	0	0.48(0.29, 0.66)
Middle SDI	Female	5	2	2001	2006	1	-8.5938	0	-1.4(-1.75, -1.05)
Middle SDI	Female	5	3	2006	2009	1	-9.4624	0	-4.75(-5.8, -3.69)
Middle SDI	Female	5	4	2009	2015	1	-33.1577	0	-3.66(-3.9, -3.43)
Middle SDI	Female	5	5	2015	2019	0	0.3768	0.712	0.06(-0.29, 0.41)
Middle SDI	Male	5	0	1990	1995	1	-2.7787	0.016	-0.21(-0.37, -0.05)
Middle SDI	Male	5	1	1995	2001	1	16.6338	0	1.24(1.08, 1.41)
Middle SDI	Male	5	2	2001	2006	1	-3.2084	0.007	-0.34(-0.57, -0.11)
Middle SDI	Male	5	3	2006	2009	1	-14.5057	0	-4.77(-5.46, -4.08)
Middle SDI	Male	5	4	2009	2014	1	-35.9862	0	-3.71(-3.93, -3.49)
Middle SDI	Male	5	5	2014	2019	1	-13.4899	0	-1.01(-1.17, -0.85)
North Africa and Middle East	Both	5	0	1990	1998	1	11.3127	0	0.39(0.32, 0.46)

North Africa and Middle East	Both	5	1	1998	2001	0	-1.0015	0.335	-0.32(-0.99, 0.37)
North Africa and Middle East	Both	5	2	2001	2004	1	-17.2423	0	-5.38(-6.03, -4.72)
North Africa and Middle East	Both	5	3	2004	2009	1	-22.2569	0	-2.33(-2.56, -2.11)
North Africa and Middle East	Both	5	4	2009	2017	1	-33.2773	0	-1.52(-1.62, -1.42)
North Africa and Middle East	Both	5	5	2017	2019	1	5.5593	0	2.02(1.23, 2.82)
North Africa and Middle East	Female	3	0	1990	2001	1	7.0849	0	0.2(0.14, 0.26)
North Africa and Middle East	Female	3	1	2001	2004	1	-11.8393	0	-4.93(-5.78, -4.08)
North Africa and Middle East	Female	3	2	2004	2017	1	-78.1873	0	-2.09(-2.14, -2.03)
North Africa and Middle East	Female	3	3	2017	2019	1	3.4736	0.003	1.7(0.67, 2.74)
North Africa and Middle East	Male	5	0	1990	1998	1	12.8334	0	0.48(0.4, 0.56)
North Africa and Middle East	Male	5	1	1998	2001	0	-1.5009	0.157	-0.5(-1.21, 0.22)
North Africa and Middle East	Male	5	2	2001	2004	1	-17.3144	0	-5.81(-6.51, -5.1)
North Africa and Middle East	Male	5	3	2004	2009	1	-20.8528	0	-2.39(-2.64, -2.15)
North Africa and Middle East	Male	5	4	2009	2017	1	-25.2392	0	-1.26(-1.36, -1.15)
North Africa and Middle East	Male	5	5	2017	2019	1	5.9453	0	2.31(1.47, 3.17)
Oceania	Both	5	0	1990	1993	0	0.0442	0.965	0.01(-0.61, 0.64)
Oceania	Both	5	1	1993	1996	0	-2.1322	0.053	-1.21(-2.42, 0.02)
Oceania	Both	5	2	1996	1999	1	-9.507	0	-5.14(-6.27, -4)
Oceania	Both	5	3	1999	2011	1	-69.1508	0	-2.72(-2.8, -2.63)
Oceania	Both	5	4	2011	2016	1	-7.261	0	-1.4(-1.81, -0.98)
Oceania	Both	5	5	2016	2019	0	-1.325	0.208	-0.41(-1.08, 0.26)
Oceania	Female	5	0	1990	1996	1	-17.993	0	-1.43(-1.6, -1.26)
Oceania	Female	5	1	1996	1999	1	-13.569	0	-6.15(-7.09, -5.2)
Oceania	Female	5	2	1999	2004	1	-28.3106	0	-4.28(-4.6, -3.96)
Oceania	Female	5	3	2004	2009	1	-17.4804	0	-2.74(-3.08, -2.41)
Oceania	Female	5	4	2009	2014	1	-10.2113	0	-1.64(-1.98, -1.29)

Oceania	Female	5	5	2014	2019	0	-0.6383	0.534	-0.07(-0.32, 0.17)
Oceania	Male	5	0	1990	1995	0	-0.322	0.753	-0.03(-0.22, 0.17)
Oceania	Male	5	1	1995	2000	1	-33.0211	0	-4.1(-4.36, -3.84)
Oceania	Male	5	2	2000	2006	1	-24.1142	0	-2.24(-2.43, -2.04)
Oceania	Male	5	3	2006	2010	1	-14.9971	0	-3.26(-3.72, -2.79)
Oceania	Male	5	4	2010	2017	1	-20.4962	0	-1.5(-1.66, -1.34)
Oceania	Male	5	5	2017	2019	0	0.0788	0.938	0.04(-0.94, 1.02)
South Asia	Both	5	0	1990	1995	0	-1.7555	0.103	-0.12(-0.27, 0.03)
South Asia	Both	5	1	1995	2001	1	36.1082	0	2.5(2.35, 2.66)
South Asia	Both	5	2	2001	2004	1	5.5718	0	1.74(1.06, 2.42)
South Asia	Both	5	3	2004	2010	1	7.9941	0	0.56(0.41, 0.71)
South Asia	Both	5	4	2010	2015	1	10.0893	0	1.01(0.8, 1.23)
South Asia	Both	5	5	2015	2019	1	-13.6827	0	-1.39(-1.61, -1.17)
South Asia	Female	5	0	1990	1993	1	-6.9813	0	-0.7(-0.92, -0.48)
South Asia	Female	5	1	1993	1996	1	2.3623	0.034	0.46(0.04, 0.89)
South Asia	Female	5	2	1996	2000	1	23.1269	0	2.31(2.09, 2.53)
South Asia	Female	5	3	2000	2011	1	101.0214	0	1.59(1.56, 1.63)
South Asia	Female	5	4	2011	2016	1	10.6086	0	0.67(0.54, 0.81)
South Asia	Female	5	5	2016	2019	1	-3.9823	0.002	-0.4(-0.62, -0.18)
South Asia	Male	5	0	1990	1995	0	-1.6688	0.119	-0.11(-0.26, 0.03)
South Asia	Male	5	1	1995	2001	1	37.265	0	2.57(2.42, 2.72)
South Asia	Male	5	2	2001	2004	1	5.9825	0	1.87(1.19, 2.56)
South Asia	Male	5	3	2004	2010	1	3.695	0.003	0.26(0.11, 0.41)
South Asia	Male	5	4	2010	2015	1	10.7415	0	1.09(0.87, 1.31)
South Asia	Male	5	5	2015	2019	1	-17.852	0	-1.8(-2.02, -1.59)
Southeast Asia	Both	5	0	1990	1995	1	10.2371	0	1.48(1.17, 1.8)

Southeast Asia	Both	5	1	1995	2001	1	-2.4033	0.032	-0.33(-0.63, -0.03)
Southeast Asia	Both	5	2	2001	2006	1	-9.1508	0	-1.8(-2.22, -1.38)
Southeast Asia	Both	5	3	2006	2009	1	-18.564	0	-11.24(-12.46, -10)
Southeast Asia	Both	5	4	2009	2015	1	-32.8963	0	-4.8(-5.11, -4.5)
Southeast Asia	Both	5	5	2015	2019	0	-0.13	0.899	-0.03(-0.5, 0.45)
Southeast Asia	Female	4	0	1990	1996	1	7.0903	0	0.82(0.58, 1.07)
Southeast Asia	Female	4	1	1996	2006	1	-20.443	0	-1.22(-1.35, -1.1)
Southeast Asia	Female	4	2	2006	2009	1	-20.3543	0	-12.59(-13.8, -11.35)
Southeast Asia	Female	4	3	2009	2015	1	-31.7676	0	-4.83(-5.14, -4.51)
Southeast Asia	Female	4	4	2015	2019	1	4.1419	0.001	0.95(0.46, 1.44)
Southeast Asia	Male	5	0	1990	1995	1	10.2003	0	1.54(1.21, 1.87)
Southeast Asia	Male	5	1	1995	2000	0	0.1741	0.864	0.04(-0.41, 0.48)
Southeast Asia	Male	5	2	2000	2006	1	-12.1157	0	-1.74(-2.04, -1.43)
Southeast Asia	Male	5	3	2006	2009	1	-17.4857	0	-10.95(-12.22, -9.67)
Southeast Asia	Male	5	4	2009	2015	1	-31.531	0	-4.8(-5.12, -4.48)
Southeast Asia	Male	5	5	2015	2019	0	-1.588	0.136	-0.37(-0.86, 0.13)
Southern Latin America	Both	5	0	1990	1995	1	8.1637	0	1.71(1.26, 2.17)
Southern Latin America	Both	5	1	1995	2000	1	-3.3178	0.006	-0.95(-1.56, -0.33)
Southern Latin America	Both	5	2	2000	2006	0	1.0198	0.326	0.2(-0.23, 0.64)
Southern Latin America	Both	5	3	2006	2009	1	-7.5415	0	-6.47(-8.25, -4.66)
Southern Latin America	Both	5	4	2009	2015	1	-26.4268	0	-5.26(-5.68, -4.84)
Southern Latin America	Both	5	5	2015	2019	1	4.4906	0.001	1.39(0.72, 2.06)
Southern Latin America	Female	4	0	1990	1995	1	6.8656	0	1.76(1.21, 2.31)
Southern Latin America	Female	4	1	1995	2000	1	-2.9976	0.009	-1.07(-1.82, -0.31)
Southern Latin America	Female	4	2	2000	2006	0	1.583	0.133	0.4(-0.14, 0.94)
Southern Latin America	Female	4	3	2006	2015	1	-44.0869	0	-5.19(-5.43, -4.95)

Southern Latin America	Female	4	4	2015	2019	1	3.7677	0.002	1.42(0.62, 2.22)
Southern Latin America	Male	5	0	1990	1994	1	4.2475	0.001	1.82(0.89, 2.76)
Southern Latin America	Male	5	1	1994	2006	1	-3.5188	0.004	-0.31(-0.49, -0.12)
Southern Latin America	Male	5	2	2006	2009	1	-5.6551	0	-6.9(-9.41, -4.32)
Southern Latin America	Male	5	3	2009	2014	1	-13.6481	0	-5.52(-6.36, -4.67)
Southern Latin America	Male	5	4	2014	2017	0	-1.1818	0.258	-1.62(-4.5, 1.36)
Southern Latin America	Male	5	5	2017	2019	1	2.5209	0.026	3.71(0.52, 6.99)
Southern Sub-Saharan Africa	Both	4	0	1990	1994	1	-11.4716	0	-1.49(-1.76, -1.22)
Southern Sub-Saharan Africa	Both	4	1	1994	2001	1	-2.9256	0.01	-0.21(-0.36, -0.06)
Southern Sub-Saharan Africa	Both	4	2	2001	2004	1	-24.3306	0	-9.9(-10.72, -9.08)
Southern Sub-Saharan Africa	Both	4	3	2004	2014	1	-80.4926	0	-3.13(-3.21, -3.05)
Southern Sub-Saharan Africa	Both	4	4	2014	2019	1	-7.4184	0	-0.72(-0.92, -0.51)
Southern Sub-Saharan Africa	Female	4	0	1990	1994	1	-19.5581	0	-2.26(-2.5, -2.02)
Southern Sub-Saharan Africa	Female	4	1	1994	2001	1	-2.7796	0.013	-0.17(-0.31, -0.04)
Southern Sub-Saharan Africa	Female	4	2	2001	2004	1	-28.2812	0	-10.45(-11.18, -9.7)
Southern Sub-Saharan Africa	Female	4	3	2004	2015	1	-128.4666	0	-3.86(-3.93, -3.8)
Southern Sub-Saharan Africa	Female	4	4	2015	2019	1	-5.2091	0	-0.66(-0.92, -0.39)
Southern Sub-Saharan Africa	Male	5	0	1990	1993	1	-5.4172	0	-1.36(-1.89, -0.82)
Southern Sub-Saharan Africa	Male	5	1	1993	2001	1	-4.7183	0	-0.32(-0.47, -0.17)
Southern Sub-Saharan Africa	Male	5	2	2001	2004	1	-18.835	0	-9.27(-10.28, -8.25)
Southern Sub-Saharan Africa	Male	5	3	2004	2007	1	-6.9677	0	-3.53(-4.6, -2.45)
Southern Sub-Saharan Africa	Male	5	4	2007	2014	1	-29.4446	0	-2.56(-2.75, -2.38)
Southern Sub-Saharan Africa	Male	5	5	2014	2019	1	-5.4028	0	-0.63(-0.89, -0.38)
Tropical Latin America	Both	5	0	1990	1996	1	-37.6416	0	-1.69(-1.79, -1.6)
Tropical Latin America	Both	5	1	1996	2003	1	-86.8857	0	-3.76(-3.85, -3.67)
Tropical Latin America	Both	5	2	2003	2006	1	-24.3839	0	-6.13(-6.66, -5.6)

Tropical Latin America	Both	5	3	2006	2010	1	-123.5672	0	-15.72(-15.97, -15.46)
Tropical Latin America	Both	5	4	2010	2015	1	-22.1808	0	-2.06(-2.26, -1.86)
Tropical Latin America	Both	5	5	2015	2019	0	-1.6381	0.125	-0.16(-0.37, 0.05)
Tropical Latin America	Female	2	0	1990	2006	1	-68.5089	0	-3.74(-3.85, -3.62)
Tropical Latin America	Female	2	1	2006	2010	1	-21.0608	0	-15(-16.35, -13.63)
Tropical Latin America	Female	2	2	2010	2019	1	-5.4292	0	-0.83(-1.15, -0.51)
Tropical Latin America	Male	5	0	1990	1996	1	-32.6903	0	-1.33(-1.42, -1.25)
Tropical Latin America	Male	5	1	1996	2003	1	-98.4465	0	-3.84(-3.92, -3.76)
Tropical Latin America	Male	5	2	2003	2006	1	-28.9254	0	-6.46(-6.92, -5.99)
Tropical Latin America	Male	5	3	2006	2010	1	-142.0956	0	-15.9(-16.13, -15.68)
Tropical Latin America	Male	5	4	2010	2015	1	-27.949	0	-2.32(-2.49, -2.14)
Tropical Latin America	Male	5	5	2015	2019	0	-0.6486	0.528	-0.06(-0.25, 0.13)
Western Europe	Both	5	0	1990	1993	1	-10.5358	0	-2.04(-2.46, -1.63)
Western Europe	Both	5	1	1993	1998	1	-8.4416	0	-1.05(-1.32, -0.78)
Western Europe	Both	5	2	1998	2001	1	-5.3028	0	-2.08(-2.92, -1.24)
Western Europe	Both	5	3	2001	2004	1	-13.0666	0	-5.05(-5.86, -4.23)
Western Europe	Both	5	4	2004	2014	1	-62.1533	0	-2.2(-2.27, -2.12)
Western Europe	Both	5	5	2014	2019	1	5.1099	0	0.45(0.26, 0.64)
Western Europe	Female	5	0	1990	1993	1	-12.6987	0	-1.95(-2.28, -1.62)
Western Europe	Female	5	1	1993	1998	1	-10.5617	0	-1.04(-1.25, -0.83)
Western Europe	Female	5	2	1998	2001	1	-5.8038	0	-1.84(-2.51, -1.16)
Western Europe	Female	5	3	2001	2004	1	-13.201	0	-4.11(-4.77, -3.45)
Western Europe	Female	5	4	2004	2014	1	-70.4578	0	-2.01(-2.07, -1.95)
Western Europe	Female	5	5	2014	2019	1	9.2894	0	0.66(0.5, 0.81)
Western Europe	Male	5	0	1990	1993	1	-6.5938	0	-2.1(-2.78, -1.42)
Western Europe	Male	5	1	1993	1998	1	-5.1509	0	-1.06(-1.5, -0.62)

Western Europe	Male	5	2	1998	2001	1	-3.4275	0.004	-2.24(-3.63, -0.84)
Western Europe	Male	5	3	2001	2004	1	-8.8589	0	-5.67(-7, -4.32)
Western Europe	Male	5	4	2004	2014	1	-39.6154	0	-2.33(-2.45, -2.2)
Western Europe	Male	5	5	2014	2019	0	1.9982	0.067	0.3(-0.02, 0.62)
Western Sub-Saharan Africa	Both	3	0	1990	2008	1	6.44	0	0.11(0.07, 0.14)
Western Sub-Saharan Africa	Both	3	1	2008	2011	0	-1.858	0.079	-0.96(-2.04, 0.12)
Western Sub-Saharan Africa	Both	3	2	2011	2014	1	-9.4521	0	-4.79(-5.82, -3.75)
Western Sub-Saharan Africa	Both	3	3	2014	2019	1	-11.5561	0	-1.4(-1.65, -1.15)
Western Sub-Saharan Africa	Female	3	0	1990	2007	1	8.4563	0	0.14(0.11, 0.17)
Western Sub-Saharan Africa	Female	3	1	2007	2011	1	-5.2584	0	-1.23(-1.72, -0.74)
Western Sub-Saharan Africa	Female	3	2	2011	2014	1	-14.2276	0	-6.48(-7.39, -5.55)
Western Sub-Saharan Africa	Female	3	3	2014	2019	1	-13.51	0	-1.46(-1.69, -1.24)
Western Sub-Saharan Africa	Male	5	0	1990	1995	1	2.8185	0.015	0.25(0.06, 0.44)
Western Sub-Saharan Africa	Male	5	1	1995	2000	1	-2.3187	0.037	-0.28(-0.55, -0.02)
Western Sub-Saharan Africa	Male	5	2	2000	2008	1	5.1136	0	0.26(0.15, 0.38)
Western Sub-Saharan Africa	Male	5	3	2008	2011	0	-1.7076	0.111	-0.66(-1.48, 0.18)
Western Sub-Saharan Africa	Male	5	4	2011	2014	1	-9.6989	0	-3.69(-4.49, -2.88)
Western Sub-Saharan Africa	Male	5	5	2014	2019	1	-14.8254	0	-1.33(-1.52, -1.14)

Supplementary Table 5: The joinpoint regression results of DALYs in children and adolescents from 1990-2019 at country and region level

Location	Sex	Joinpoint	Segment	Segment.Start	Segment.End	Significant_indicator	Test.Statistic	P.Value	APC_95CI
Andean Latin America	Both	3	0	1990	1992	0	-1.1021	0.284	-1.8(-5.13, 1.65)
Andean Latin America	Both	3	1	1992	2005	1	-37.5967	0	-3.43(-3.62, -3.24)
Andean Latin America	Both	3	2	2005	2014	1	-43.8382	0	-7.68(-8.03, -7.33)
Andean Latin America	Both	3	3	2014	2019	1	-9.9322	0	-4.01(-4.83, -3.17)
Andean Latin America	Female	5	0	1990	1994	1	-4.0889	0.001	-1.8(-2.73, -0.85)
Andean Latin America	Female	5	1	1994	2001	1	-26.6181	0	-5.79(-6.25, -5.33)
Andean Latin America	Female	5	2	2001	2005	1	-3.1263	0.008	-2(-3.36, -0.62)
Andean Latin America	Female	5	3	2005	2009	1	-15.0943	0	-9.49(-10.77, -8.19)
Andean Latin America	Female	5	4	2009	2014	1	-16.3019	0	-7.09(-7.99, -6.18)
Andean Latin America	Female	5	5	2014	2019	1	-14.2932	0	-4.88(-5.59, -4.15)
Andean Latin America	Male	2	0	1990	2005	1	-44.1032	0	-3.01(-3.15, -2.87)
Andean Latin America	Male	2	1	2005	2014	1	-42.3628	0	-7.79(-8.16, -7.43)
Andean Latin America	Male	2	2	2014	2019	1	-8.6759	0	-3.77(-4.66, -2.89)
Caribbean	Both	3	0	1990	1995	1	-12.5123	0	-3.7(-4.3, -3.09)
Caribbean	Both	3	1	1995	2002	1	-22.024	0	-4.91(-5.36, -4.45)
Caribbean	Both	3	2	2002	2010	1	-11.6765	0	-2.22(-2.62, -1.83)
Caribbean	Both	3	3	2010	2019	1	7.439	0	1.04(0.75, 1.34)
Caribbean	Female	2	0	1990	2002	1	-38.0497	0	-4.02(-4.24, -3.81)
Caribbean	Female	2	1	2002	2010	1	-7.3293	0	-1.91(-2.44, -1.37)
Caribbean	Female	2	2	2010	2019	0	0.3407	0.737	0.06(-0.32, 0.44)
Caribbean	Male	5	0	1990	1995	1	-15.9832	0	-3.56(-4.03, -3.08)
Caribbean	Male	5	1	1995	1998	1	-5.7746	0	-5.84(-7.94, -3.7)
Caribbean	Male	5	2	1998	2003	1	-14.0996	0	-4.61(-5.3, -3.92)

Caribbean	Male	5	3	2003	2010	1	-11.276	0	-2.15(-2.56, -1.75)
Caribbean	Male	5	4	2010	2014	0	1.4554	0.169	0.88(-0.42, 2.2)
Caribbean	Male	5	5	2014	2019	1	6.9723	0	1.94(1.33, 2.54)
Central Asia	Both	5	0	1990	1996	1	36.0367	0	9.98(9.35, 10.61)
Central Asia	Both	5	1	1996	2001	1	7.9715	0	4.1(2.97, 5.23)
Central Asia	Both	5	2	2001	2006	1	-6.1635	0	-3.03(-4.07, -1.98)
Central Asia	Both	5	3	2006	2011	1	-19.6893	0	-9.38(-10.36, -8.4)
Central Asia	Both	5	4	2011	2015	1	-16.4053	0	-12.52(-14.05, -10.97)
Central Asia	Both	5	5	2015	2019	1	-9.8751	0	-5.1(-6.18, -4.01)
Central Asia	Female	5	0	1990	1994	1	15.1458	0	9.09(7.75, 10.45)
Central Asia	Female	5	1	1994	1999	1	7.1546	0	4.26(2.95, 5.58)
Central Asia	Female	5	2	1999	2005	1	-3.6392	0.003	-1.51(-2.39, -0.61)
Central Asia	Female	5	3	2005	2011	1	-16.0455	0	-6.56(-7.41, -5.7)
Central Asia	Female	5	4	2011	2016	1	-20.5091	0	-11.66(-12.8, -10.5)
Central Asia	Female	5	5	2016	2019	1	-4.8329	0	-4.66(-6.67, -2.6)
Central Asia	Male	5	0	1990	1996	1	38.2213	0	10.66(10.03, 11.29)
Central Asia	Male	5	1	1996	2001	1	9.0269	0	4.64(3.51, 5.78)
Central Asia	Male	5	2	2001	2006	1	-6.2773	0	-3.08(-4.12, -2.03)
Central Asia	Male	5	3	2006	2011	1	-21.3741	0	-10.16(-11.13, -9.18)
Central Asia	Male	5	4	2011	2015	1	-16.7614	0	-12.69(-14.2, -11.15)
Central Asia	Male	5	5	2015	2019	1	-9.2334	0	-4.76(-5.85, -3.67)
Central Europe	Both	5	0	1990	1999	0	0.769	0.456	0.12(-0.22, 0.46)
Central Europe	Both	5	1	1999	2004	1	-18.911	0	-9.8(-10.86, -8.73)
Central Europe	Both	5	2	2004	2007	1	-3.1255	0.008	-5.28(-8.77, -1.66)
Central Europe	Both	5	3	2007	2011	1	-10.8805	0	-9.34(-11.09, -7.56)
Central Europe	Both	5	4	2011	2015	1	-7.1284	0	-6.16(-7.96, -4.34)

Central Europe	Both	5	5	2015	2019	1	-2.5575	0.024	-1.43(-2.61, -0.22)
Central Europe	Female	5	0	1990	1993	0	-1.9682	0.071	-2.08(-4.31, 0.21)
Central Europe	Female	5	1	1993	1997	0	0.615	0.549	0.66(-1.64, 3.02)
Central Europe	Female	5	2	1997	2000	1	-2.3385	0.036	-4.99(-9.38, -0.39)
Central Europe	Female	5	3	2000	2003	1	-4.5213	0.001	-9.36(-13.52, -5)
Central Europe	Female	5	4	2003	2014	1	-42.7568	0	-7.07(-7.41, -6.73)
Central Europe	Female	5	5	2014	2019	1	-6.0866	0	-3.01(-4.06, -1.95)
Central Europe	Male	5	0	1990	1999	1	2.6241	0.021	0.48(0.08, 0.87)
Central Europe	Male	5	1	1999	2004	1	-17.105	0	-10.02(-11.21, -8.81)
Central Europe	Male	5	2	2004	2007	1	-2.5777	0.023	-5.04(-9.07, -0.83)
Central Europe	Male	5	3	2007	2010	1	-5.4623	0	-10.57(-14.44, -6.53)
Central Europe	Male	5	4	2010	2015	1	-10.7767	0	-6.65(-7.93, -5.35)
Central Europe	Male	5	5	2015	2019	0	-1.4985	0.158	-0.95(-2.31, 0.42)
Central Latin America	Both	4	0	1990	1996	1	-12.3833	0	-2.42(-2.83, -2.01)
Central Latin America	Both	4	1	1996	2006	1	-32.6792	0	-3.44(-3.66, -3.22)
Central Latin America	Both	4	2	2006	2009	1	-5.9627	0	-7.15(-9.57, -4.67)
Central Latin America	Both	4	3	2009	2014	1	-9.3079	0	-3.81(-4.66, -2.96)
Central Latin America	Both	4	4	2014	2019	1	-5.114	0	-1.52(-2.15, -0.89)
Central Latin America	Female	3	0	1990	1995	1	-7.8897	0	-2.84(-3.58, -2.09)
Central Latin America	Female	3	1	1995	2006	1	-33.6614	0	-4.25(-4.51, -3.99)
Central Latin America	Female	3	2	2006	2010	1	-7.9479	0	-6.63(-8.3, -4.92)
Central Latin America	Female	3	3	2010	2019	1	-11.942	0	-2.07(-2.43, -1.71)
Central Latin America	Male	4	0	1990	1996	1	-11.9549	0	-2.2(-2.58, -1.81)
Central Latin America	Male	4	1	1996	2006	1	-32.3542	0	-3.18(-3.38, -2.97)
Central Latin America	Male	4	2	2006	2009	1	-6.427	0	-7.17(-9.42, -4.86)
Central Latin America	Male	4	3	2009	2014	1	-10.9479	0	-4.17(-4.95, -3.37)

Central Latin America	Male	4	4	2014	2019	1	-5.1773	0	-1.42(-2, -0.84)
Central Sub-Saharan Africa	Both	1	0	1990	2014	1	-22.6258	0	-1.19(-1.29, -1.08)
Central Sub-Saharan Africa	Both	1	1	2014	2019	1	-16.3573	0	-9.71(-10.86, -8.54)
Central Sub-Saharan Africa	Female	2	0	1990	2005	1	-6.9681	0	-0.73(-0.95, -0.52)
Central Sub-Saharan Africa	Female	2	1	2005	2015	1	-8.8087	0	-1.98(-2.44, -1.52)
Central Sub-Saharan Africa	Female	2	2	2015	2019	1	-13.3474	0	-11.13(-12.74, -9.48)
Central Sub-Saharan Africa	Male	1	0	1990	2014	1	-23.6746	0	-1.21(-1.31, -1.1)
Central Sub-Saharan Africa	Male	1	1	2014	2019	1	-16.4108	0	-9.66(-10.8, -8.5)
East Asia	Both	4	0	1990	1997	1	-27.1639	0	-3.95(-4.25, -3.64)
East Asia	Both	4	1	1997	2004	1	-11.8675	0	-2.12(-2.49, -1.74)
East Asia	Both	4	2	2004	2010	1	-23.9072	0	-5.63(-6.11, -5.14)
East Asia	Both	4	3	2010	2015	1	-54.8247	0	-17.61(-18.23, -16.99)
East Asia	Both	4	4	2015	2019	1	-18.1391	0	-6.47(-7.19, -5.73)
East Asia	Female	3	0	1990	2001	1	-33.1709	0	-3.12(-3.31, -2.93)
East Asia	Female	3	1	2001	2011	1	-52.2325	0	-6.2(-6.44, -5.96)
East Asia	Female	3	2	2011	2014	1	-18.2304	0	-22.21(-24.42, -19.94)
East Asia	Female	3	3	2014	2019	1	-28.5453	0	-8.68(-9.29, -8.07)
East Asia	Male	4	0	1990	1998	1	-38.204	0	-4.28(-4.51, -4.04)
East Asia	Male	4	1	1998	2004	1	-3.0089	0.008	-0.66(-1.12, -0.19)
East Asia	Male	4	2	2004	2010	1	-26.4026	0	-5.83(-6.28, -5.38)
East Asia	Male	4	3	2010	2015	1	-59.2373	0	-17.57(-18.14, -17)
East Asia	Male	4	4	2015	2019	1	-18.5557	0	-6.12(-6.79, -5.44)
Eastern Europe	Both	5	0	1990	1995	1	9.5194	0	8.61(6.59, 10.67)
Eastern Europe	Both	5	1	1995	1998	0	-0.0303	0.976	-0.12(-8.52, 9.05)
Eastern Europe	Both	5	2	1998	2001	0	1.5316	0.15	6.37(-2.5, 16.05)
Eastern Europe	Both	5	3	2001	2007	1	-2.613	0.021	-2.36(-4.26, -0.41)

Eastern Europe	Both	5	4	2007	2013	1	-19.5149	0	-16.18(-17.8, -14.53)
Eastern Europe	Both	5	5	2013	2019	1	-4.326	0.001	-3.13(-4.66, -1.58)
Eastern Europe	Female	5	0	1990	1994	1	5.1391	0	5.69(3.26, 8.18)
Eastern Europe	Female	5	1	1994	2002	1	6.062	0	2.9(1.86, 3.96)
Eastern Europe	Female	5	2	2002	2007	0	-2.1	0.056	-2.38(-4.77, 0.07)
Eastern Europe	Female	5	3	2007	2010	1	-3.8873	0.002	-13.44(-20.11, -6.21)
Eastern Europe	Female	5	4	2010	2013	1	-5.4913	0	-17.54(-23.57, -11.04)
Eastern Europe	Female	5	5	2013	2019	1	-3.1312	0.008	-1.94(-3.26, -0.61)
Eastern Europe	Male	3	0	1990	1995	1	6.7578	0	9.57(6.51, 12.72)
Eastern Europe	Male	3	1	1995	2006	1	2.9579	0.008	1.43(0.42, 2.46)
Eastern Europe	Male	3	2	2006	2013	1	-16.4049	0	-15.71(-17.52, -13.85)
Eastern Europe	Male	3	3	2013	2019	1	-3.2951	0.004	-3.67(-5.93, -1.36)
Eastern Sub-Saharan Africa	Both	3	0	1990	1994	0	1.2305	0.234	0.42(-0.3, 1.15)
Eastern Sub-Saharan Africa	Both	3	1	1994	2010	1	-29.1657	0	-1.28(-1.37, -1.19)
Eastern Sub-Saharan Africa	Both	3	2	2010	2013	1	-5.8242	0	-6.07(-8.06, -3.88)
Eastern Sub-Saharan Africa	Both	3	3	2013	2019	1	-20.8182	0	-3.58(-3.93, -3.23)
Eastern Sub-Saharan Africa	Female	4	0	1990	1993	0	-0.3882	0.703	-0.2(-1.3, 0.91)
Eastern Sub-Saharan Africa	Female	4	1	1993	2007	1	-32.1744	0	-1.65(-1.76, -1.54)
Eastern Sub-Saharan Africa	Female	4	2	2007	2010	1	-2.6765	0.017	-2.52(-4.47, -0.53)
Eastern Sub-Saharan Africa	Female	4	3	2010	2013	1	-5.7398	0	-5.15(-6.98, -3.28)
Eastern Sub-Saharan Africa	Female	4	4	2013	2019	1	-23.4178	0	-3.71(-4.04, -3.38)
Eastern Sub-Saharan Africa	Male	3	0	1990	1994	0	1.8816	0.075	0.66(-0.07, 1.4)
Eastern Sub-Saharan Africa	Male	3	1	1994	2010	1	-26.1331	0	-1.17(-1.26, -1.08)
Eastern Sub-Saharan Africa	Male	3	2	2010	2013	1	-5.8319	0	-6.11<br>(-8.21, -3.96)
Eastern Sub-Saharan Africa	Male	3	3	2013	2019	1	-20.2625	0	-3.57(-3.93, -3.21)

Global	Both	5	0	1990	1996	1	-3.2623	0.006	-0.65(-1.08, -0.22)
Global	Both	5	1	1996	2000	0	1.0593	0.309	0.61(-0.63, 1.87)
Global	Both	5	2	2000	2006	0	-1.2833	0.222	-0.33(-0.88, 0.23)
Global	Both	5	3	2006	2011	1	-6.2214	0	-2.23(-2.99, -1.46)
Global	Both	5	4	2011	2016	1	-11.4537	0	-4.02(-4.76, -3.28)
Global	Both	5	5	2016	2019	1	-4.0289	0.001	-2.42(-3.69, -1.13)
Global	Female	4	0	1990	1995	1	-4.6359	0	-1.16(-1.68, -0.63)
Global	Female	4	1	1995	2005	1	-3.3908	0.004	-0.34(-0.56, -0.13)
Global	Female	4	2	2005	2011	1	-10.3695	0	-2.46(-2.96, -1.96)
Global	Female	4	3	2011	2016	1	-11.3022	0	-3.76(-4.44, -3.06)
Global	Female	4	4	2016	2019	1	-4.5995	0	-2.55(-3.71, -1.39)
Global	Male	5	0	1990	1996	1	-2.2024	0.046	-0.5(-0.98, -0.01)
Global	Male	5	1	1996	2000	0	1.1436	0.273	0.76(-0.67, 2.2)
Global	Male	5	2	2000	2006	0	-0.5724	0.577	-0.17(-0.79, 0.46)
Global	Male	5	3	2006	2011	1	-5.2917	0	-2.13(-2.99, -1.27)
Global	Male	5	4	2011	2017	1	-14.0555	0	-4(-4.6, -3.39)
Global	Male	5	5	2017	2019	0	-1.1352	0.277	-1.53(-4.39, 1.41)
High SDI	Both	5	0	1990	1993	1	-27.3587	0	-17.88(-19.15, -16.59)
High SDI	Both	5	1	1993	1997	1	-14.703	0	-10.05(-11.44, -8.64)
High SDI	Both	5	2	1997	2000	1	-10.141	0	-13.2(-15.77, -10.54)
High SDI	Both	5	3	2000	2004	1	-13.0809	0	-8.54(-9.88, -7.18)
High SDI	Both	5	4	2004	2015	1	-48.0784	0	-5.08(-5.3, -4.85)
High SDI	Both	5	5	2015	2019	1	-4.7835	0	-2.2(-3.18, -1.21)
High SDI	Female	5	0	1990	1993	1	-33.8656	0	-19.12(-20.21, -18.02)
High SDI	Female	5	1	1993	1997	1	-16.1064	0	-9.7(-10.92, -8.45)
High SDI	Female	5	2	1997	2000	1	-11.5708	0	-13.16(-15.42, -10.84)

High SDI	Female	5	3	2000	2004	1	-15.1678	0	-8.6(-9.76, -7.42)
High SDI	Female	5	4	2004	2015	1	-53.9471	0	-4.97(-5.17, -4.78)
High SDI	Female	5	5	2015	2019	1	-5.4872	0	-2.13(-2.96, -1.3)
High SDI	Male	5	0	1990	1994	1	-32.8542	0	-16.03(-16.99, -15.06)
High SDI	Male	5	1	1994	1997	1	-5.509	0	-8.87(-12.13, -5.49)
High SDI	Male	5	2	1997	2000	1	-9.0125	0	-13.58(-16.55, -10.5)
High SDI	Male	5	3	2000	2004	1	-11.3555	0	-8.47(-10, -6.91)
High SDI	Male	5	4	2004	2015	1	-41.8916	0	-5.13(-5.39, -4.87)
High SDI	Male	5	5	2015	2019	1	-4.2138	0.001	-2.24(-3.37, -1.1)
High-middle SDI	Both	3	0	1990	2000	0	0.0244	0.981	0.01(-0.27, 0.28)
High-middle SDI	Both	3	1	2000	2006	1	-8.3522	0	-3.11(-3.87, -2.34)
High-middle SDI	Both	3	2	2006	2016	1	-46.8812	0	-7.1(-7.41, -6.8)
High-middle SDI	Both	3	3	2016	2019	0	0.3181	0.754	0.29(-1.6, 2.22)
High-middle SDI	Female	4	0	1990	2000	1	-5.7352	0	-0.47(-0.64, -0.3)
High-middle SDI	Female	4	1	2000	2005	1	-15.5992	0	-4.92(-5.56, -4.26)
High-middle SDI	Female	4	2	2005	2011	1	-34.8655	0	-7.69(-8.13, -7.24)
High-middle SDI	Female	4	3	2011	2014	1	-9.7784	0	-9.97(-11.99, -7.89)
High-middle SDI	Female	4	4	2014	2019	1	-8.1915	0	-2.06(-2.59, -1.53)
High-middle SDI	Male	3	0	1990	2000	0	0.8339	0.415	0.13(-0.2, 0.47)
High-middle SDI	Male	3	1	2000	2006	1	-5.2656	0	-2.43(-3.38, -1.47)
High-middle SDI	Male	3	2	2006	2016	1	-36.9341	0	-6.91(-7.28, -6.53)
High-middle SDI	Male	3	3	2016	2019	0	0.1318	0.897	0.15(-2.16, 2.51)
Low SDI	Both	3	0	1990	1997	1	-5.5571	0	-0.72(-0.99, -0.45)
Low SDI	Both	3	1	1997	2006	1	-2.1387	0.046	-0.22(-0.43, 0)
Low SDI	Both	3	2	2006	2015	1	-28.9166	0	-2.89(-3.09, -2.68)
Low SDI	Both	3	3	2015	2019	1	-12.2125	0	-3.67(-4.28, -3.05)

Low SDI	Female	3	0	1990	1998	1	-10.0753	0	-1.1(-1.33, -0.87)
Low SDI	Female	3	1	1998	2006	1	-3.9785	0.001	-0.53(-0.81, -0.25)
Low SDI	Female	3	2	2006	2017	1	-39.5932	0	-2.9(-3.05, -2.75)
Low SDI	Female	3	3	2017	2019	1	-4.8103	0	-4.55(-6.46, -2.6)
Low SDI	Male	3	0	1990	1997	1	-4.2586	0	-0.59(-0.88, -0.3)
Low SDI	Male	3	1	1997	2006	0	-0.988	0.336	-0.11(-0.33, 0.12)
Low SDI	Male	3	2	2006	2015	1	-27.574	0	-2.87(-3.08, -2.65)
Low SDI	Male	3	3	2015	2019	1	-11.7718	0	-3.68(-4.32, -3.04)
Low-middle SDI	Both	3	0	1990	2006	1	-3.1121	0.006	-0.24(-0.39, -0.08)
Low-middle SDI	Both	3	1	2006	2011	1	-4.2717	0	-2.55(-3.77, -1.31)
Low-middle SDI	Both	3	2	2011	2017	1	-12.4265	0	-5.29(-6.15, -4.42)
Low-middle SDI	Both	3	3	2017	2019	0	-0.3101	0.76	-0.62(-4.72, 3.65)
Low-middle SDI	Female	4	0	1990	1995	1	-3.8552	0.001	-1.31(-2.02, -0.59)
Low-middle SDI	Female	4	1	1995	2005	1	2.4499	0.026	0.32(0.04, 0.6)
Low-middle SDI	Female	4	2	2005	2011	1	-8.9303	0	-2.75(-3.39, -2.1)
Low-middle SDI	Female	4	3	2011	2016	1	-11.2047	0	-4.9(-5.8, -3.99)
Low-middle SDI	Female	4	4	2016	2019	1	-3.2518	0.005	-2.36(-3.87, -0.83)
Low-middle SDI	Male	3	0	1990	2006	1	-3.6966	0.002	-0.3(-0.47, -0.13)
Low-middle SDI	Male	3	1	2006	2011	1	-3.6363	0.002	-2.32(-3.62, -0.99)
Low-middle SDI	Male	3	2	2011	2017	1	-12.5284	0	-5.54(-6.44, -4.64)
Low-middle SDI	Male	3	3	2017	2019	0	-0.1171	0.908	-0.25(-4.67, 4.37)
Middle SDI	Both	5	0	1990	1997	1	-6.2494	0	-1.02(-1.37, -0.67)
Middle SDI	Both	5	1	1997	2000	0	0.6037	0.556	0.74(-1.89, 3.44)
Middle SDI	Both	5	2	2000	2005	1	-5.1593	0	-1.98(-2.79, -1.15)
Middle SDI	Both	5	3	2005	2011	1	-11.2337	0	-3.03(-3.6, -2.46)
Middle SDI	Both	5	4	2011	2016	1	-13.9115	0	-5.31(-6.11, -4.51)

Middle SDI	Both	5	5	2016	2019	1	-3.1017	0.008	-2.01(-3.39, -0.62)
Middle SDI	Female	5	0	1990	2000	1	-18.1201	0	-1.5(-1.67, -1.32)
Middle SDI	Female	5	1	2000	2005	1	-8.895	0	-2.87(-3.56, -2.18)
Middle SDI	Female	5	2	2005	2008	1	-4.6297	0	-4.67(-6.77, -2.52)
Middle SDI	Female	5	3	2008	2011	1	-2.4397	0.03	-2.56(-4.78, -0.3)
Middle SDI	Female	5	4	2011	2016	1	-16.9048	0	-5.67(-6.38, -4.97)
Middle SDI	Female	5	5	2016	2019	1	-2.5567	0.024	-1.48(-2.71, -0.23)
Middle SDI	Male	5	0	1990	1996	1	-4.7451	0	-0.98(-1.43, -0.54)
Middle SDI	Male	5	1	1996	2000	0	1.5537	0.144	0.98(-0.38, 2.35)
Middle SDI	Male	5	2	2000	2005	1	-3.7356	0.002	-1.47(-2.3, -0.62)
Middle SDI	Male	5	3	2005	2011	1	-10.5015	0	-2.88(-3.46, -2.29)
Middle SDI	Male	5	4	2011	2016	1	-13.5713	0	-5.3(-6.12, -4.48)
Middle SDI	Male	5	5	2016	2019	1	-3.2811	0.006	-2.15(-3.54, -0.74)
North Africa and Middle East	Both	5	0	1990	1992	1	-5.5236	0	-2.94(-4.07, -1.8)
North Africa and Middle East	Both	5	1	1992	2000	1	-13.8144	0	-0.98(-1.14, -0.83)
North Africa and Middle East	Both	5	2	2000	2004	1	-22.1256	0	-5.67(-6.21, -5.13)
North Africa and Middle East	Both	5	3	2004	2012	1	-52.8037	0	-4.04(-4.2, -3.88)
North Africa and Middle East	Both	5	4	2012	2017	1	-30.0472	0	-5.55(-5.94, -5.17)
North Africa and Middle East	Both	5	5	2017	2019	1	-3.2224	0.007	-2(-3.31, -0.66)
North Africa and Middle East	Female	3	0	1990	2000	1	-19.6543	0	-2.06(-2.27, -1.84)
North Africa and Middle East	Female	3	1	2000	2012	1	-49.683	0	-4.45(-4.63, -4.27)
North Africa and Middle East	Female	3	2	2012	2017	1	-12.7185	0	-5.76(-6.67, -4.83)
North Africa and Middle East	Female	3	3	2017	2019	0	-1.712	0.103	-2.59(-5.66, 0.59)
North Africa and Middle East	Male	5	0	1990	1992	1	-5.468	0	-3(-4.16, -1.83)
North Africa and Middle East	Male	5	1	1992	2000	1	-8.1745	0	-0.59(-0.75, -0.44)
North Africa and Middle East	Male	5	2	2000	2004	1	-22.7831	0	-6.03(-6.59, -5.48)

North Africa and Middle East	Male	5	3	2004	2012	1	-51.0042	0	-3.97(-4.13, -3.8)
North Africa and Middle East	Male	5	4	2012	2017	1	-28.8478	0	-5.43(-5.82, -5.03)
North Africa and Middle East	Male	5	5	2017	2019	1	-2.8422	0.014	-1.82(-3.19, -0.44)
Oceania	Both	2	0	1990	1996	1	-7.2309	0	-1.66(-2.14, -1.19)
Oceania	Both	2	1	1996	2009	1	-37.6331	0	-3.23(-3.41, -3.06)
Oceania	Both	2	2	2009	2019	1	-14.5371	0	-1.8(-2.05, -1.54)
Oceania	Female	3	0	1990	1996	1	-9.1269	0	-2(-2.45, -1.54)
Oceania	Female	3	1	1996	2004	1	-29.6642	0	-5.25(-5.61, -4.89)
Oceania	Female	3	2	2004	2009	1	-7.386	0	-3.26(-4.17, -2.35)
Oceania	Female	3	3	2009	2019	1	-11.164	0	-1.26(-1.49, -1.02)
Oceania	Male	2	0	1990	1996	1	-6.2252	0	-1.61(-2.14, -1.08)
Oceania	Male	2	1	1996	2010	1	-35.347	0	-3.04(-3.22, -2.87)
Oceania	Male	2	2	2010	2019	1	-10.7244	0	-1.77(-2.11, -1.43)
South Asia	Both	2	0	1990	1996	0	-1.4576	0.159	-0.9(-2.16, 0.38)
South Asia	Both	2	1	1996	2006	1	2.2029	0.038	0.69(0.04, 1.34)
South Asia	Both	2	2	2006	2019	1	-19.7137	0	-3.54(-3.9, -3.17)
South Asia	Female	2	0	1990	1995	1	-3.0101	0.006	-1.61(-2.71, -0.5)
South Asia	Female	2	1	1995	2005	1	3.7096	0.001	0.74(0.33, 1.16)
South Asia	Female	2	2	2005	2019	1	-28.2708	0	-2.83(-3.03, -2.62)
South Asia	Male	1	0	1990	2007	0	1.0008	0.326	0.16(-0.17, 0.48)
South Asia	Male	1	1	2007	2019	1	-14.9894	0	-3.85(-4.37, -3.33)
Southeast Asia	Both	5	0	1990	1998	1	-32.2489	0	-1.49(-1.59, -1.39)
Southeast Asia	Both	5	1	1998	2002	1	-14.0172	0	-2.85(-3.28, -2.41)
Southeast Asia	Both	5	2	2002	2005	1	-4.487	0.001	-1.8(-2.65, -0.94)
Southeast Asia	Both	5	3	2005	2009	1	-31.9933	0	-6.29(-6.7, -5.87)
Southeast Asia	Both	5	4	2009	2014	1	-32.0805	0	-4.12(-4.39, -3.85)

Southeast Asia	Both	5	5	2014	2019	1	-14.5068	0	-1.33(-1.52, -1.13)
Southeast Asia	Female	5	0	1990	1999	1	-58.9249	0	-2.02(-2.09, -1.95)
Southeast Asia	Female	5	1	1999	2005	1	-40.0585	0	-3.14(-3.31, -2.98)
Southeast Asia	Female	5	2	2005	2009	1	-49.204	0	-8.21(-8.56, -7.87)
Southeast Asia	Female	5	3	2009	2012	1	-18.5987	0	-6.31(-7.02, -5.6)
Southeast Asia	Female	5	4	2012	2015	1	-12.6802	0	-4.37(-5.1, -3.64)
Southeast Asia	Female	5	5	2015	2019	1	-8.2306	0	-0.92(-1.16, -0.68)
Southeast Asia	Male	5	0	1990	1998	1	-21.6994	0	-1.29(-1.42, -1.16)
Southeast Asia	Male	5	1	1998	2002	1	-11.0071	0	-2.87(-3.42, -2.31)
Southeast Asia	Male	5	2	2002	2005	1	-2.2166	0.045	-1.17(-2.3, -0.03)
Southeast Asia	Male	5	3	2005	2009	1	-21.5865	0	-5.52(-6.06, -4.98)
Southeast Asia	Male	5	4	2009	2014	1	-21.4007	0	-3.52(-3.87, -3.17)
Southeast Asia	Male	5	5	2014	2019	1	-11.5468	0	-1.36(-1.62, -1.11)
Southern Latin America	Both	3	0	1990	2001	1	-16.9444	0	-2.29(-2.57, -2.01)
Southern Latin America	Both	3	1	2001	2007	0	-1.0929	0.288	-0.51(-1.47, 0.47)
Southern Latin America	Both	3	2	2007	2015	1	-21.987	0	-6.26(-6.83, -5.68)
Southern Latin America	Both	3	3	2015	2019	1	-5.2943	0	-3.79(-5.24, -2.31)
Southern Latin America	Female	5	0	1990	1994	1	-8.0155	0	-4.35(-5.49, -3.2)
Southern Latin America	Female	5	1	1994	1998	0	-1.4349	0.175	-1.24(-3.09, 0.64)
Southern Latin America	Female	5	2	1998	2003	1	-5.2996	0	-2.87(-4.02, -1.71)
Southern Latin America	Female	5	3	2003	2007	0	0.6886	0.503	0.6(-1.26, 2.49)
Southern Latin America	Female	5	4	2007	2015	1	-27.1451	0	-6.72(-7.24, -6.2)
Southern Latin America	Female	5	5	2015	2019	1	-4.923	0	-3.19(-4.56, -1.81)
Southern Latin America	Male	3	0	1990	2000	1	-15.798	0	-2.16(-2.45, -1.88)
Southern Latin America	Male	3	1	2000	2007	1	-2.3245	0.031	-0.71(-1.34, -0.07)
Southern Latin America	Male	3	2	2007	2015	1	-24.671	0	-6.07(-6.57, -5.57)

Southern Latin America	Male	3	3	2015	2019	1	-6.4643	0	-4.06(-5.34, -2.77)
Southern Sub-Saharan Africa	Both	5	0	1990	1996	1	-2.6985	0.018	-0.88(-1.59, -0.18)
Southern Sub-Saharan Africa	Both	5	1	1996	2001	0	1.9172	0.077	1.16(-0.15, 2.49)
Southern Sub-Saharan Africa	Both	5	2	2001	2004	1	-4.9644	0	-9.27(-13.02, -5.34)
Southern Sub-Saharan Africa	Both	5	3	2004	2009	1	-3.4871	0.004	-2.32(-3.74, -0.89)
Southern Sub-Saharan Africa	Both	5	4	2009	2014	1	-10.1683	0	-7.03(-8.46, -5.58)
Southern Sub-Saharan Africa	Both	5	5	2014	2019	1	-7.1017	0	-3.67(-4.76, -2.57)
Southern Sub-Saharan Africa	Female	5	0	1990	1996	1	-15.1458	0	-6.18(-7.03, -5.32)
Southern Sub-Saharan Africa	Female	5	1	1996	2001	1	2.4297	0.03	2.04(0.22, 3.89)
Southern Sub-Saharan Africa	Female	5	2	2001	2005	1	-4.7852	0	-6.56(-9.38, -3.65)
Southern Sub-Saharan Africa	Female	5	3	2005	2010	0	-2.0183	0.065	-1.89(-3.87, 0.13)
Southern Sub-Saharan Africa	Female	5	4	2010	2014	1	-5.7928	0	-8.48(-11.45, -5.4)
Southern Sub-Saharan Africa	Female	5	5	2014	2019	1	-6.5571	0	-4.21(-5.56, -2.84)
Southern Sub-Saharan Africa	Male	4	0	1990	2001	1	3.7941	0.002	0.55(0.24, 0.85)
Southern Sub-Saharan Africa	Male	4	1	2001	2005	1	-8.1364	0	-8.49(-10.59, -6.35)
Southern Sub-Saharan Africa	Male	4	2	2005	2008	0	-0.1018	0.92	-0.24(-5.09, 4.86)
Southern Sub-Saharan Africa	Male	4	3	2008	2014	1	-12.5248	0	-6.73(-7.82, -5.62)
Southern Sub-Saharan Africa	Male	4	4	2014	2019	1	-6.1186	0	-3.59(-4.8, -2.36)
Tropical Latin America	Both	4	0	1990	1992	1	-3.033	0.008	-5.15(-8.59, -1.58)
Tropical Latin America	Both	4	1	1992	2001	1	-14.6741	0	-2.79(-3.18, -2.39)
Tropical Latin America	Both	4	2	2001	2006	1	-7.5896	0	-4.22(-5.37, -3.06)
Tropical Latin America	Both	4	3	2006	2009	1	-7.5019	0	-13.12(-16.51, -9.6)
Tropical Latin America	Both	4	4	2009	2019	1	-33.8446	0	-5.06(-5.37, -4.75)
Tropical Latin America	Female	4	0	1990	1994	1	-3.1321	0.006	-1.67(-2.78, -0.54)
Tropical Latin America	Female	4	1	1994	2006	1	-26.3188	0	-3.08(-3.32, -2.83)
Tropical Latin America	Female	4	2	2006	2009	1	-8.0951	0	-13.98(-17.31, -10.52)

Tropical Latin America	Female	4	3	2009	2014	1	-11.8101	0	-7.1(-8.32, -5.87)
Tropical Latin America	Female	4	4	2014	2019	1	-5.6576	0	-2.54(-3.47, -1.6)
Tropical Latin America	Male	4	0	1990	1992	1	-3.177	0.006	-5.97(-9.76, -2.03)
Tropical Latin America	Male	4	1	1992	2001	1	-13.5061	0	-2.87(-3.31, -2.42)
Tropical Latin America	Male	4	2	2001	2006	1	-7.1645	0	-4.47(-5.75, -3.17)
Tropical Latin America	Male	4	3	2006	2009	1	-6.5012	0	-12.55(-16.29, -8.64)
Tropical Latin America	Male	4	4	2009	2019	1	-30.6274	0	-5.1(-5.44, -4.76)
Western Europe	Both	5	0	1990	1994	1	-11.5185	0	-6.04(-7.14, -4.94)
Western Europe	Both	5	1	1994	2001	1	-12.6119	0	-3.55(-4.14, -2.95)
Western Europe	Both	5	2	2001	2004	1	-4.9259	0	-8.18(-11.55, -4.68)
Western Europe	Both	5	3	2004	2008	1	-5.0219	0	-4.25(-6.02, -2.44)
Western Europe	Both	5	4	2008	2014	1	-17.6957	0	-6.24(-6.98, -5.5)
Western Europe	Both	5	5	2014	2019	0	0.1693	0.868	0.06(-0.72, 0.84)
Western Europe	Female	5	0	1990	1994	1	-13.3247	0	-4.81(-5.57, -4.04)
Western Europe	Female	5	1	1994	2000	1	-9.5743	0	-2.5(-3.05, -1.94)
Western Europe	Female	5	2	2000	2005	1	-18.1791	0	-6.74(-7.5, -5.96)
Western Europe	Female	5	3	2005	2009	1	-6.2597	0	-3.72(-4.97, -2.45)
Western Europe	Female	5	4	2009	2013	1	-10.738	0	-6.13(-7.31, -4.93)
Western Europe	Female	5	5	2013	2019	1	-2.2673	0.041	-0.45(-0.87, -0.02)
Western Europe	Male	5	0	1990	1994	1	-10.3388	0	-6.61(-7.93, -5.26)
Western Europe	Male	5	1	1994	2001	1	-11.0491	0	-3.84(-4.58, -3.1)
Western Europe	Male	5	2	2001	2004	1	-4.0584	0.001	-8.38(-12.55, -4.01)
Western Europe	Male	5	3	2004	2007	0	-1.7489	0.104	-3.68(-8.03, 0.88)
Western Europe	Male	5	4	2007	2014	1	-20.0555	0	-6.6 (-7.29, -5.91)
Western Europe	Male	5	5	2014	2019	0	-0.1651	0.871	-0.07(-1.02, 0.88)

Western Sub-Saharan Africa	Both	3	0	1990	1992	0	0.5166	0.611	0.57(-1.72, 2.92)
Western Sub-Saharan Africa	Both	3	1	1992	2006	1	-13.027	0	-0.77(-0.89, -0.65)
Western Sub-Saharan Africa	Both	3	2	2006	2010	1	-4.7688	0	-2.62(-3.74, -1.48)
Western Sub-Saharan Africa	Both	3	3	2010	2019	1	-39.9548	0	-4.07(-4.28, -3.86)
Western Sub-Saharan Africa	Female	5	0	1990	1993	0	0.3671	0.719	0.13(-0.62, 0.88)
Western Sub-Saharan Africa	Female	5	1	1993	2006	1	-28.4226	0	-1.22(-1.31, -1.13)
Western Sub-Saharan Africa	Female	5	2	2006	2011	1	-12.6446	0	-2.85(-3.33, -2.37)
Western Sub-Saharan Africa	Female	5	3	2011	2014	1	-8.1438	0	-5.62(-7.05, -4.16)
Western Sub-Saharan Africa	Female	5	4	2014	2017	1	-4.317	0.001	-3.11(-4.62, -1.56)
Western Sub-Saharan Africa	Female	5	5	2017	2019	1	-7.3749	0	-5.42(-6.95, -3.86)
Western Sub-Saharan Africa	Male	4	0	1990	1993	0	0.5768	0.572	0.29(-0.77, 1.36)
Western Sub-Saharan Africa	Male	4	1	1993	1998	1	-3.5963	0.002	-1.12(-1.78, -0.46)
Western Sub-Saharan Africa	Male	4	2	1998	2005	1	-2.8611	0.011	-0.47(-0.82, -0.12)
Western Sub-Saharan Africa	Male	4	3	2005	2010	1	-7.2691	0	-2.25(-2.9, -1.6)
Western Sub-Saharan Africa	Male	4	4	2010	2019	1	-43.3111	0	-3.94(-4.13, -3.75)

Supplementary Table 6: The incidence of hepatitis B-associated cirrhosis in children and adolescents from 1990 to 2019 at the national and territorial levels

Location	<20 years	<5 years	5-9 years	10-14 years	15-19 years
Afghanistan	0.111(0.037-0.246)	0.078(0.023-0.165)	0.110(0.028-0.260)	0.123(0.026-0.306)	0.148(0.013-0.458)
Albania	0.080(0.024-0.205)	0.040(0.011-0.089)	0.053(0.014-0.125)	0.073(0.015-0.194)	0.140(0.027-0.407)
Algeria	0.065(0.020-0.152)	0.039(0.010-0.087)	0.061(0.015-0.148)	0.073(0.016-0.190)	0.096(0.016-0.284)
American Samoa	0.222(0.078-0.470)	0.044(0.009-0.108)	0.122(0.036-0.286)	0.227(0.062-0.524)	0.460(0.128-1.083)
Andorra	0.025(0.006-0.070)	0.004(0.000-0.011)	0.008(0.001-0.021)	0.022(0.004-0.062)	0.058(0.011-0.168)
Angola	0.281(0.106-0.577)	0.096(0.031-0.209)	0.206(0.066-0.465)	0.355(0.109-0.800)	0.614(0.175-1.482)
Antigua and Barbuda	0.020(0.005-0.055)	0.007(0.002-0.018)	0.012(0.002-0.030)	0.018(0.003-0.055)	0.038(0.006-0.121)
Argentina	0.028(0.007-0.073)	0.008(0.001-0.019)	0.016(0.003-0.039)	0.029(0.005-0.078)	0.059(0.010-0.172)
Armenia	0.109(0.032-0.269)	0.024(0.005-0.056)	0.067(0.016-0.163)	0.131(0.030-0.338)	0.243(0.059-0.637)
Australia	0.027(0.008-0.069)	0.011(0.001-0.028)	0.017(0.002-0.045)	0.025(0.003-0.074)	0.055(0.008-0.164)
Austria	0.029(0.007-0.076)	0.006(0.001-0.014)	0.011(0.002-0.028)	0.028(0.006-0.077)	0.071(0.014-0.197)
Azerbaijan	0.168(0.052-0.371)	0.030(0.006-0.074)	0.094(0.026-0.216)	0.200(0.054-0.495)	0.380(0.098-0.965)
Bahamas	0.023(0.006-0.062)	0.008(0.002-0.020)	0.013(0.003-0.034)	0.021(0.004-0.057)	0.044(0.007-0.138)
Bahrain	0.074(0.020-0.179)	0.041(0.011-0.089)	0.065(0.016-0.164)	0.078(0.017-0.200)	0.104(0.018-0.300)
Bangladesh	0.116(0.035-0.267)	0.103(0.031-0.224)	0.105(0.024-0.254)	0.101(0.023-0.261)	0.152(0.030-0.435)
Barbados	0.021(0.005-0.058)	0.008(0.002-0.019)	0.013(0.002-0.032)	0.020(0.003-0.056)	0.040(0.006-0.119)
Belarus	0.064(0.017-0.159)	0.019(0.004-0.045)	0.036(0.008-0.089)	0.063(0.012-0.167)	0.158(0.033-0.459)
Belgium	0.020(0.004-0.053)	0.003(0.000-0.009)	0.007(0.001-0.019)	0.019(0.003-0.055)	0.050(0.009-0.149)
Belize	0.025(0.006-0.064)	0.009(0.002-0.021)	0.015(0.003-0.037)	0.023(0.004-0.063)	0.051(0.009-0.141)
Benin	0.360(0.141-0.730)	0.173(0.058-0.361)	0.278(0.089-0.624)	0.411(0.119-0.935)	0.727(0.200-1.775)
Bermuda	0.040(0.011-0.100)	0.016(0.004-0.036)	0.026(0.006-0.058)	0.038(0.008-0.105)	0.075(0.015-0.227)
Bhutan	0.173(0.052-0.408)	0.091(0.026-0.200)	0.131(0.033-0.295)	0.165(0.039-0.410)	0.296(0.063-0.799)
Bolivia	0.022(0.005-0.052)	0.012(0.003-0.028)	0.015(0.003-0.039)	0.021(0.004-0.057)	0.044(0.007-0.132)

Bosnia and Herzegovina	0.083(0.024-0.202)	0.036(0.008-0.087)	0.053(0.012-0.126)	0.077(0.017-0.205)	0.152(0.028-0.443)
Botswana	0.132(0.041-0.296)	0.042(0.012-0.096)	0.085(0.024-0.198)	0.139(0.037-0.343)	0.277(0.068-0.717)
Brazil	0.032(0.012-0.070)	0.006(0.001-0.012)	0.011(0.001-0.023)	0.025(0.007-0.060)	0.085(0.023-0.209)
Brunei Darussalam	0.167(0.056-0.372)	0.103(0.030-0.230)	0.110(0.026-0.256)	0.146(0.030-0.375)	0.290(0.058-0.823)
Bulgaria	0.107(0.031-0.255)	0.045(0.011-0.102)	0.068(0.017-0.160)	0.102(0.023-0.260)	0.214(0.046-0.571)
Burkina Faso	0.330(0.124-0.670)	0.172(0.060-0.358)	0.261(0.078-0.602)	0.371(0.102-0.855)	0.646(0.149-1.648)
Burundi	0.091(0.030-0.206)	0.030(0.008-0.066)	0.062(0.017-0.153)	0.107(0.026-0.271)	0.213(0.051-0.557)
Cabo Verde	0.390(0.144-0.816)	0.148(0.048-0.320)	0.269(0.084-0.620)	0.415(0.116-0.943)	0.749(0.208-1.878)
Cambodia	0.190(0.061-0.425)	0.032(0.009-0.072)	0.084(0.024-0.203)	0.187(0.047-0.443)	0.500(0.125-1.261)
Cameroon	0.441(0.164-0.909)	0.189(0.064-0.397)	0.324(0.100-0.714)	0.489(0.136-1.114)	0.862(0.212-2.119)
Canada	0.010(0.002-0.027)	0.001(0.000-0.003)	0.002(0.000-0.007)	0.007(0.001-0.022)	0.028(0.005-0.086)
Central African Republic	0.335(0.117-0.717)	0.087(0.028-0.183)	0.194(0.060-0.444)	0.378(0.108-0.870)	0.821(0.216-2.076)
Chad	0.633(0.259-1.256)	0.319(0.115-0.663)	0.521(0.171-1.118)	0.739(0.209-1.673)	1.263(0.335-3.062)
Chile	0.031(0.008-0.081)	0.008(0.001-0.021)	0.018(0.004-0.045)	0.031(0.006-0.086)	0.067(0.013-0.192)
China	0.061(0.026-0.125)	0.015(0.003-0.031)	0.024(0.005-0.048)	0.051(0.016-0.117)	0.158(0.047-0.354)
Colombia	0.009(0.002-0.026)	0.004(0.001-0.010)	0.004(0.000-0.011)	0.006(0.001-0.020)	0.020(0.002-0.068)
Comoros	0.159(0.053-0.357)	0.043(0.012-0.093)	0.093(0.027-0.217)	0.166(0.045-0.392)	0.347(0.091-0.899)
Congo	0.218(0.078-0.459)	0.069(0.021-0.155)	0.149(0.046-0.346)	0.260(0.074-0.586)	0.462(0.126-1.157)
Cook Islands	0.066(0.019-0.156)	0.016(0.003-0.040)	0.039(0.009-0.095)	0.070(0.015-0.173)	0.137(0.025-0.383)
Costa Rica	0.010(0.002-0.027)	0.004(0.001-0.010)	0.004(0.000-0.012)	0.007(0.001-0.022)	0.024(0.004-0.072)
Croatia	0.090(0.028-0.219)	0.038(0.010-0.087)	0.054(0.015-0.128)	0.083(0.020-0.211)	0.177(0.039-0.516)
Cuba	0.021(0.005-0.056)	0.008(0.002-0.018)	0.013(0.002-0.032)	0.019(0.003-0.054)	0.040(0.006-0.121)
Cyprus	0.017(0.004-0.046)	0.003(0.000-0.009)	0.006(0.001-0.017)	0.017(0.003-0.047)	0.044(0.009-0.132)
Czechia	0.081(0.024-0.202)	0.034(0.007-0.082)	0.053(0.013-0.125)	0.081(0.017-0.216)	0.170(0.033-0.497)
Cte d'Ivoire	0.447(0.171-0.919)	0.198(0.068-0.419)	0.336(0.109-0.736)	0.511(0.147-1.200)	0.899(0.217-2.259)
Korea	0.075(0.021-0.192)	0.007(0.000-0.024)	0.021(0.000-0.059)	0.059(0.009-0.161)	0.200(0.044-0.545)

Democratic Republic of the Congo	0.319(0.116-0.690)	0.088(0.028-0.191)	0.200(0.063-0.443)	0.377(0.112-0.847)	0.739(0.209-1.855)
Denmark	0.055(0.015-0.139)	0.010(0.001-0.026)	0.020(0.004-0.051)	0.052(0.011-0.139)	0.130(0.030-0.357)
Djibouti	0.158(0.055-0.351)	0.048(0.014-0.107)	0.102(0.029-0.238)	0.180(0.048-0.427)	0.375(0.096-0.980)
Dominica	0.019(0.004-0.052)	0.007(0.001-0.019)	0.012(0.002-0.030)	0.018(0.003-0.049)	0.036(0.005-0.108)
Dominican Republic	0.031(0.008-0.080)	0.012(0.003-0.029)	0.020(0.005-0.048)	0.031(0.006-0.085)	0.066(0.012-0.190)
Ecuador	0.019(0.005-0.047)	0.007(0.002-0.017)	0.010(0.002-0.024)	0.017(0.004-0.045)	0.042(0.009-0.122)
Egypt	0.245(0.083-0.522)	0.107(0.031-0.227)	0.216(0.062-0.497)	0.288(0.074-0.681)	0.392(0.077-1.049)
El Salvador	0.011(0.002-0.032)	0.004(0.001-0.010)	0.004(0.000-0.011)	0.008(0.001-0.022)	0.029(0.004-0.089)
Equatorial Guinea	0.444(0.165-0.929)	0.114(0.036-0.242)	0.263(0.084-0.590)	0.501(0.157-1.098)	0.907(0.265-2.150)
Eritrea	0.131(0.041-0.305)	0.036(0.011-0.080)	0.077(0.022-0.185)	0.139(0.035-0.341)	0.300(0.073-0.789)
Estonia	0.061(0.016-0.148)	0.018(0.004-0.045)	0.035(0.008-0.083)	0.060(0.011-0.154)	0.144(0.029-0.411)
Eswatini	0.111(0.036-0.253)	0.035(0.010-0.079)	0.070(0.019-0.170)	0.118(0.031-0.305)	0.237(0.059-0.627)
Ethiopia	0.301(0.141-0.557)	0.090(0.039-0.170)	0.204(0.086-0.384)	0.353(0.126-0.701)	0.642(0.203-1.412)
Fiji	0.086(0.028-0.193)	0.021(0.004-0.051)	0.054(0.014-0.126)	0.097(0.023-0.228)	0.188(0.044-0.497)
Finland	0.051(0.014-0.130)	0.009(0.001-0.023)	0.018(0.003-0.045)	0.047(0.010-0.122)	0.126(0.029-0.356)
France	0.029(0.007-0.076)	0.005(0.001-0.013)	0.010(0.002-0.026)	0.027(0.006-0.072)	0.071(0.014-0.201)
Gabon	0.168(0.058-0.363)	0.058(0.018-0.125)	0.117(0.036-0.267)	0.192(0.054-0.440)	0.325(0.084-0.836)
Gambia	0.376(0.140-0.761)	0.157(0.055-0.326)	0.272(0.090-0.612)	0.412(0.115-0.954)	0.738(0.187-1.827)
Georgia	0.086(0.026-0.197)	0.017(0.004-0.038)	0.041(0.012-0.096)	0.094(0.024-0.231)	0.223(0.053-0.556)
Germany	0.027(0.006-0.072)	0.004(0.000-0.012)	0.009(0.001-0.025)	0.025(0.005-0.072)	0.067(0.013-0.189)
Ghana	0.323(0.117-0.675)	0.130(0.041-0.272)	0.232(0.072-0.512)	0.358(0.103-0.823)	0.623(0.170-1.544)
Greece	0.030(0.008-0.077)	0.005(0.000-0.014)	0.011(0.002-0.028)	0.028(0.005-0.076)	0.073(0.015-0.210)
Greenland	0.011(0.002-0.030)	0.001(0.000-0.004)	0.002(0.000-0.009)	0.009(0.001-0.026)	0.033(0.006-0.098)
Grenada	0.021(0.005-0.057)	0.007(0.001-0.019)	0.012(0.002-0.032)	0.019(0.003-0.055)	0.041(0.007-0.124)
Guam	0.309(0.113-0.667)	0.056(0.013-0.132)	0.168(0.051-0.378)	0.342(0.101-0.802)	0.737(0.216-1.774)
Guatemala	0.014(0.003-0.039)	0.005(0.001-0.013)	0.006(0.001-0.016)	0.010(0.001-0.029)	0.038(0.006-0.112)

Guinea	0.438(0.168-0.865)	0.236(0.083-0.499)	0.353(0.110-0.769)	0.494(0.138-1.108)	0.819(0.199-1.975)
Guinea-Bissau	0.482(0.185-0.985)	0.200(0.068-0.426)	0.347(0.109-0.792)	0.540(0.150-1.240)	0.983(0.249-2.516)
Guyana	0.027(0.006-0.065)	0.009(0.002-0.022)	0.016(0.003-0.042)	0.025(0.005-0.069)	0.056(0.010-0.156)
Haiti	0.030(0.009-0.077)	0.015(0.004-0.034)	0.021(0.004-0.051)	0.030(0.005-0.081)	0.061(0.009-0.188)
Honduras	0.008(0.002-0.024)	0.004(0.001-0.010)	0.004(0.000-0.011)	0.006(0.001-0.020)	0.020(0.003-0.065)
Hungary	0.195(0.065-0.430)	0.086(0.022-0.206)	0.127(0.034-0.277)	0.186(0.043-0.476)	0.366(0.075-0.965)
Iceland	0.035(0.009-0.088)	0.006(0.000-0.016)	0.013(0.002-0.034)	0.034(0.007-0.090)	0.087(0.018-0.243)
India	0.318(0.145-0.600)	0.145(0.066-0.270)	0.233(0.100-0.440)	0.323(0.105-0.671)	0.544(0.162-1.230)
Indonesia	0.073(0.024-0.151)	0.011(0.002-0.024)	0.030(0.008-0.065)	0.070(0.016-0.155)	0.168(0.021-0.414)
Iran	0.056(0.025-0.107)	0.028(0.011-0.055)	0.050(0.019-0.098)	0.066(0.020-0.141)	0.088(0.018-0.217)
Iraq	0.082(0.025-0.188)	0.045(0.012-0.102)	0.074(0.019-0.179)	0.092(0.020-0.238)	0.120(0.021-0.343)
Ireland	0.029(0.008-0.074)	0.005(0.000-0.013)	0.010(0.002-0.027)	0.028(0.006-0.076)	0.073(0.016-0.213)
Israel	0.017(0.004-0.046)	0.003(0.000-0.009)	0.007(0.001-0.018)	0.018(0.003-0.051)	0.046(0.009-0.133)
Italy	0.036(0.016-0.068)	0.011(0.004-0.021)	0.018(0.006-0.035)	0.033(0.012-0.069)	0.076(0.025-0.166)
Jamaica	0.016(0.004-0.044)	0.007(0.001-0.016)	0.010(0.002-0.027)	0.015(0.002-0.046)	0.029(0.004-0.090)
Japan	0.106(0.048-0.195)	0.066(0.028-0.123)	0.072(0.025-0.138)	0.092(0.029-0.202)	0.180(0.049-0.397)
Jordan	0.060(0.017-0.152)	0.033(0.008-0.075)	0.054(0.012-0.133)	0.066(0.013-0.179)	0.086(0.013-0.254)
Kazakhstan	0.132(0.036-0.306)	0.025(0.005-0.059)	0.074(0.018-0.180)	0.159(0.037-0.394)	0.353(0.083-0.920)
Kenya	0.189(0.082-0.363)	0.039(0.016-0.075)	0.102(0.043-0.200)	0.205(0.073-0.410)	0.432(0.132-0.980)
Kiribati	0.159(0.054-0.345)	0.029(0.006-0.067)	0.087(0.025-0.204)	0.176(0.045-0.398)	0.403(0.098-1.035)
Kuwait	0.064(0.018-0.163)	0.035(0.009-0.080)	0.059(0.014-0.142)	0.073(0.015-0.192)	0.097(0.015-0.289)
Kyrgyzstan	0.144(0.043-0.348)	0.017(0.002-0.046)	0.076(0.018-0.184)	0.175(0.044-0.420)	0.387(0.102-0.990)
Lao People's Democratic Republic	0.124(0.043-0.275)	0.025(0.005-0.057)	0.062(0.015-0.151)	0.130(0.031-0.306)	0.293(0.072-0.742)
Latvia	0.057(0.014-0.142)	0.016(0.003-0.039)	0.032(0.007-0.075)	0.055(0.010-0.146)	0.135(0.026-0.379)
Lebanon	0.102(0.033-0.229)	0.059(0.018-0.129)	0.098(0.026-0.232)	0.118(0.028-0.296)	0.153(0.028-0.441)
Lesotho	0.128(0.041-0.295)	0.037(0.010-0.085)	0.077(0.021-0.185)	0.131(0.034-0.313)	0.266(0.066-0.690)

Liberia	0.417(0.161-0.850)	0.215(0.072-0.451)	0.323(0.103-0.708)	0.448(0.125-1.003)	0.736(0.178-1.891)
Libya	0.064(0.018-0.152)	0.037(0.010-0.083)	0.056(0.014-0.137)	0.067(0.015-0.178)	0.089(0.014-0.252)
Lithuania	0.073(0.018-0.183)	0.019(0.004-0.048)	0.038(0.009-0.089)	0.066(0.013-0.185)	0.171(0.033-0.489)
Luxembourg	0.029(0.007-0.078)	0.005(0.000-0.013)	0.010(0.001-0.027)	0.028(0.005-0.080)	0.072(0.013-0.213)
Madagascar	0.146(0.050-0.320)	0.042(0.013-0.092)	0.090(0.026-0.226)	0.160(0.042-0.384)	0.339(0.090-0.872)
Malawi	0.151(0.049-0.335)	0.041(0.012-0.089)	0.088(0.025-0.210)	0.159(0.042-0.379)	0.338(0.081-0.908)
Malaysia	0.185(0.062-0.416)	0.032(0.006-0.076)	0.083(0.021-0.191)	0.188(0.051-0.435)	0.424(0.107-1.052)
Maldives	0.067(0.020-0.165)	0.014(0.003-0.034)	0.036(0.008-0.090)	0.078(0.016-0.196)	0.168(0.032-0.475)
Mali	0.450(0.184-0.898)	0.245(0.087-0.509)	0.354(0.112-0.803)	0.490(0.141-1.088)	0.877(0.228-2.140)
Malta	0.021(0.005-0.056)	0.004(0.001-0.010)	0.007(0.001-0.020)	0.020(0.004-0.054)	0.053(0.010-0.152)
Marshall Islands	0.116(0.039-0.256)	0.024(0.005-0.057)	0.066(0.018-0.159)	0.125(0.031-0.295)	0.264(0.062-0.668)
Mauritania	0.464(0.186-0.954)	0.195(0.067-0.417)	0.338(0.115-0.756)	0.509(0.152-1.144)	0.881(0.237-2.098)
Mauritius	0.115(0.037-0.265)	0.017(0.003-0.041)	0.045(0.011-0.106)	0.102(0.026-0.252)	0.245(0.066-0.645)
Mexico	0.012(0.005-0.026)	0.005(0.002-0.011)	0.006(0.001-0.012)	0.009(0.002-0.022)	0.028(0.007-0.070)
Micronesia (Federated States of)	0.141(0.045-0.316)	0.025(0.005-0.063)	0.071(0.020-0.165)	0.140(0.036-0.331)	0.310(0.079-0.815)
Monaco	0.024(0.006-0.065)	0.004(0.000-0.011)	0.008(0.001-0.021)	0.022(0.004-0.062)	0.058(0.011-0.167)
Mongolia	0.136(0.041-0.323)	0.028(0.006-0.064)	0.083(0.022-0.197)	0.168(0.041-0.428)	0.374(0.088-0.973)
Montenegro	0.079(0.023-0.202)	0.035(0.008-0.080)	0.052(0.013-0.124)	0.075(0.015-0.200)	0.143(0.023-0.433)
Morocco	0.068(0.020-0.163)	0.041(0.012-0.090)	0.063(0.015-0.150)	0.074(0.015-0.190)	0.094(0.014-0.277)
Mozambique	0.163(0.056-0.353)	0.048(0.014-0.103)	0.107(0.031-0.256)	0.201(0.053-0.477)	0.379(0.090-0.955)
Myanmar	0.184(0.059-0.418)	0.026(0.006-0.061)	0.068(0.017-0.165)	0.163(0.042-0.391)	0.482(0.126-1.233)
Namibia	0.117(0.038-0.273)	0.037(0.010-0.081)	0.076(0.022-0.179)	0.128(0.034-0.325)	0.250(0.066-0.657)
Nauru	0.129(0.043-0.290)	0.024(0.005-0.058)	0.070(0.020-0.168)	0.140(0.037-0.328)	0.317(0.079-0.823)
Nepal	0.169(0.049-0.395)	0.081(0.023-0.178)	0.129(0.033-0.304)	0.170(0.038-0.433)	0.284(0.052-0.798)
Netherlands	0.037(0.010-0.094)	0.006(0.001-0.016)	0.013(0.002-0.033)	0.034(0.007-0.090)	0.087(0.020-0.245)
New Zealand	0.029(0.013-0.054)	0.014(0.005-0.027)	0.022(0.006-0.044)	0.028(0.006-0.064)	0.051(0.009-0.124)

Nicaragua	0.010(0.002-0.028)	0.004(0.001-0.010)	0.004(0.001-0.011)	0.007(0.001-0.022)	0.026(0.004-0.081)
Niger	0.523(0.212-1.074)	0.273(0.099-0.561)	0.419(0.137-0.919)	0.601(0.165-1.378)	1.068(0.268-2.580)
Nigeria	0.519(0.256-0.942)	0.246(0.114-0.451)	0.428(0.181-0.799)	0.602(0.205-1.222)	0.919(0.254-2.108)
Niue	0.104(0.033-0.235)	0.021(0.005-0.051)	0.054(0.015-0.135)	0.106(0.027-0.255)	0.226(0.056-0.588)
North Macedonia	0.080(0.023-0.200)	0.036(0.008-0.083)	0.053(0.013-0.129)	0.078(0.016-0.202)	0.148(0.028-0.438)
Northern Mariana Islands	0.459(0.161-0.975)	0.062(0.016-0.146)	0.197(0.063-0.450)	0.405(0.118-0.924)	0.846(0.260-2.003)
Norway	0.030(0.011-0.062)	0.007(0.002-0.016)	0.018(0.006-0.039)	0.031(0.009-0.068)	0.060(0.016-0.144)
Oman	0.062(0.018-0.147)	0.036(0.009-0.082)	0.058(0.014-0.140)	0.071(0.015-0.186)	0.094(0.014-0.272)
Pakistan	0.310(0.144-0.565)	0.136(0.060-0.258)	0.258(0.110-0.483)	0.367(0.130-0.757)	0.521(0.135-1.223)
Palau	0.131(0.043-0.302)	0.024(0.006-0.056)	0.065(0.018-0.152)	0.126(0.033-0.296)	0.291(0.076-0.760)
Palestine	0.071(0.020-0.169)	0.042(0.011-0.097)	0.066(0.016-0.164)	0.079(0.016-0.211)	0.102(0.014-0.292)
Panama	0.008(0.002-0.023)	0.004(0.001-0.010)	0.004(0.000-0.011)	0.006(0.001-0.020)	0.020(0.003-0.064)
Papua New Guinea	0.078(0.026-0.179)	0.019(0.002-0.049)	0.049(0.010-0.115)	0.090(0.018-0.226)	0.192(0.044-0.523)
Paraguay	0.036(0.009-0.092)	0.006(0.000-0.015)	0.011(0.000-0.029)	0.030(0.005-0.087)	0.097(0.020-0.283)
Peru	0.029(0.007-0.071)	0.012(0.003-0.029)	0.019(0.004-0.046)	0.028(0.005-0.078)	0.059(0.010-0.170)
Philippines	0.081(0.033-0.156)	0.018(0.006-0.036)	0.047(0.016-0.094)	0.090(0.028-0.189)	0.183(0.054-0.407)
Poland	0.114(0.050-0.215)	0.042(0.016-0.082)	0.088(0.036-0.168)	0.123(0.042-0.254)	0.209(0.058-0.490)
Portugal	0.017(0.004-0.048)	0.002(0.000-0.007)	0.004(0.000-0.013)	0.014(0.003-0.040)	0.040(0.007-0.118)
Puerto Rico	0.024(0.006-0.065)	0.008(0.002-0.021)	0.014(0.003-0.035)	0.021(0.004-0.058)	0.043(0.007-0.128)
Qatar	0.076(0.023-0.180)	0.043(0.012-0.098)	0.071(0.018-0.175)	0.086(0.019-0.217)	0.115(0.020-0.344)
South Korea	0.425(0.159-0.905)	0.290(0.093-0.637)	0.300(0.085-0.654)	0.369(0.094-0.868)	0.693(0.150-1.767)
Republic of Moldova	0.076(0.020-0.188)	0.020(0.004-0.047)	0.040(0.009-0.098)	0.069(0.015-0.176)	0.170(0.036-0.483)
Romania	0.106(0.030-0.249)	0.042(0.010-0.095)	0.065(0.015-0.151)	0.099(0.021-0.250)	0.212(0.045-0.587)
Russian Federation	0.074(0.030-0.153)	0.017(0.006-0.036)	0.042(0.016-0.084)	0.077(0.026-0.167)	0.184(0.057-0.438)
Rwanda	0.079(0.022-0.196)	0.023(0.006-0.053)	0.049(0.012-0.116)	0.085(0.018-0.217)	0.174(0.037-0.486)
Saint Kitts and Nevis	0.021(0.005-0.055)	0.008(0.002-0.019)	0.013(0.002-0.031)	0.019(0.003-0.055)	0.040(0.007-0.119)

Saint Lucia	0.023(0.005-0.059)	0.008(0.002-0.020)	0.013(0.003-0.033)	0.020(0.003-0.056)	0.043(0.008-0.127)
Saint Vincent and the Grenadines	0.018(0.004-0.048)	0.007(0.001-0.017)	0.011(0.002-0.029)	0.017(0.003-0.049)	0.036(0.006-0.104)
Samoa	0.191(0.066-0.415)	0.038(0.008-0.090)	0.105(0.031-0.245)	0.198(0.054-0.465)	0.414(0.109-1.020)
San Marino	0.029(0.007-0.076)	0.004(0.000-0.012)	0.009(0.001-0.024)	0.025(0.005-0.072)	0.070(0.014-0.193)
Sao Tome and Principe	0.639(0.238-1.321)	0.215(0.071-0.441)	0.436(0.139-0.964)	0.700(0.207-1.578)	1.224(0.319-3.067)
Saudi Arabia	0.059(0.015-0.145)	0.030(0.008-0.071)	0.051(0.012-0.124)	0.064(0.014-0.167)	0.086(0.014-0.251)
Senegal	0.502(0.187-1.017)	0.202(0.070-0.417)	0.359(0.115-0.808)	0.557(0.153-1.225)	0.999(0.252-2.493)
Serbia	0.070(0.019-0.182)	0.029(0.007-0.069)	0.044(0.010-0.103)	0.066(0.014-0.182)	0.132(0.025-0.393)
Seychelles	0.097(0.030-0.235)	0.017(0.003-0.041)	0.044(0.011-0.105)	0.100(0.025-0.247)	0.235(0.057-0.635)
Sierra Leone	0.444(0.169-0.920)	0.199(0.070-0.413)	0.330(0.106-0.753)	0.492(0.136-1.118)	0.854(0.211-2.172)
Singapore	0.313(0.121-0.635)	0.213(0.079-0.448)	0.224(0.074-0.477)	0.281(0.082-0.644)	0.550(0.148-1.367)
Slovakia	0.109(0.032-0.258)	0.048(0.011-0.113)	0.073(0.018-0.173)	0.107(0.023-0.275)	0.217(0.043-0.599)
Slovenia	0.085(0.027-0.198)	0.037(0.009-0.088)	0.056(0.014-0.130)	0.084(0.018-0.224)	0.172(0.034-0.482)
Solomon Islands	0.159(0.057-0.352)	0.025(0.005-0.061)	0.076(0.021-0.185)	0.169(0.047-0.386)	0.444(0.119-1.091)
Somalia	0.285(0.109-0.593)	0.100(0.032-0.212)	0.199(0.059-0.458)	0.327(0.089-0.763)	0.653(0.179-1.628)
South Africa	0.105(0.049-0.192)	0.035(0.014-0.068)	0.076(0.029-0.143)	0.120(0.042-0.245)	0.199(0.063-0.445)
South Sudan	0.226(0.084-0.476)	0.078(0.026-0.174)	0.152(0.045-0.353)	0.248(0.069-0.570)	0.485(0.132-1.212)
Spain	0.030(0.008-0.080)	0.005(0.001-0.015)	0.011(0.002-0.028)	0.029(0.006-0.078)	0.073(0.016-0.208)
Sri Lanka	0.084(0.025-0.193)	0.014(0.003-0.035)	0.037(0.009-0.089)	0.083(0.021-0.208)	0.192(0.048-0.505)
Sudan	0.064(0.020-0.149)	0.046(0.014-0.102)	0.060(0.013-0.144)	0.068(0.011-0.180)	0.087(0.006-0.269)
Suriname	0.020(0.005-0.053)	0.008(0.002-0.019)	0.013(0.003-0.034)	0.020(0.003-0.057)	0.041(0.007-0.120)
Sweden	0.033(0.013-0.069)	0.010(0.002-0.021)	0.021(0.006-0.045)	0.035(0.011-0.080)	0.067(0.018-0.168)
Switzerland	0.035(0.010-0.091)	0.006(0.001-0.017)	0.012(0.002-0.033)	0.034(0.007-0.093)	0.091(0.020-0.255)
Syrian Arab Republic	0.087(0.026-0.203)	0.052(0.014-0.113)	0.077(0.020-0.183)	0.089(0.019-0.228)	0.113(0.020-0.333)
Taiwan (Province of China)	0.060(0.014-0.166)	0.003(0.000-0.013)	0.014(0.000-0.043)	0.043(0.004-0.123)	0.156(0.029-0.465)
Tajikistan	0.161(0.047-0.363)	0.031(0.008-0.070)	0.094(0.024-0.230)	0.191(0.049-0.476)	0.394(0.094-1.008)

Thailand	0.063(0.017-0.157)	0.009(0.001-0.024)	0.024(0.005-0.064)	0.057(0.012-0.146)	0.140(0.033-0.376)
Timor-Leste	0.125(0.043-0.277)	0.022(0.005-0.052)	0.059(0.015-0.146)	0.130(0.034-0.315)	0.300(0.077-0.763)
Togo	0.455(0.175-0.912)	0.189(0.064-0.403)	0.328(0.107-0.722)	0.507(0.138-1.149)	0.928(0.256-2.301)
Tokelau	0.162(0.058-0.349)	0.038(0.008-0.091)	0.100(0.028-0.228)	0.188(0.051-0.436)	0.395(0.103-0.961)
Tonga	0.106(0.034-0.233)	0.023(0.005-0.056)	0.063(0.017-0.154)	0.116(0.028-0.271)	0.239(0.060-0.607)
Trinidad and Tobago	0.021(0.005-0.054)	0.008(0.002-0.020)	0.013(0.003-0.033)	0.020(0.003-0.056)	0.040(0.006-0.124)
Tunisia	0.026(0.006-0.070)	0.015(0.003-0.037)	0.024(0.005-0.059)	0.029(0.005-0.084)	0.038(0.005-0.114)
Turkey	0.141(0.047-0.318)	0.080(0.024-0.180)	0.126(0.036-0.300)	0.149(0.037-0.360)	0.191(0.038-0.546)
Turkmenistan	0.303(0.087-0.736)	0.045(0.011-0.102)	0.159(0.043-0.368)	0.352(0.095-0.804)	0.792(0.195-2.227)
Tuvalu	0.132(0.044-0.290)	0.026(0.005-0.063)	0.070(0.019-0.165)	0.135(0.034-0.309)	0.290(0.078-0.744)
Uganda	0.120(0.040-0.258)	0.038(0.011-0.084)	0.079(0.022-0.190)	0.137(0.035-0.328)	0.272(0.066-0.693)
Ukraine	0.149(0.060-0.316)	0.027(0.009-0.056)	0.070(0.027-0.141)	0.140(0.046-0.300)	0.380(0.123-0.894)
United Arab Emirates	0.060(0.017-0.152)	0.035(0.008-0.078)	0.054(0.012-0.134)	0.066(0.012-0.180)	0.086(0.010-0.267)
United Kingdom	0.026(0.010-0.056)	0.005(0.001-0.010)	0.010(0.003-0.022)	0.025(0.008-0.057)	0.066(0.020-0.155)
United Republic of Tanzania	0.106(0.035-0.242)	0.038(0.011-0.084)	0.072(0.019-0.173)	0.119(0.029-0.300)	0.238(0.061-0.602)
United States of America	0.005(0.002-0.012)	0.001(0.000-0.001)	0.001(0.000-0.002)	0.003(0.001-0.008)	0.015(0.005-0.037)
United States Virgin Islands	0.025(0.007-0.064)	0.009(0.002-0.022)	0.016(0.003-0.038)	0.024(0.004-0.068)	0.052(0.009-0.151)
Uruguay	0.024(0.006-0.063)	0.007(0.001-0.017)	0.014(0.003-0.036)	0.025(0.005-0.068)	0.050(0.009-0.150)
Uzbekistan	0.215(0.064-0.502)	0.033(0.008-0.077)	0.110(0.029-0.251)	0.243(0.067-0.581)	0.545(0.139-1.380)
Vanuatu	0.162(0.056-0.355)	0.033(0.007-0.079)	0.097(0.027-0.232)	0.184(0.048-0.421)	0.396(0.101-1.041)
Venezuela	0.009(0.002-0.026)	0.004(0.001-0.010)	0.004(0.000-0.012)	0.007(0.001-0.022)	0.022(0.003-0.068)
Viet Nam	0.122(0.041-0.282)	0.019(0.004-0.047)	0.050(0.014-0.119)	0.121(0.033-0.278)	0.304(0.082-0.767)
Yemen	0.070(0.020-0.164)	0.046(0.013-0.099)	0.066(0.016-0.158)	0.077(0.015-0.202)	0.098(0.013-0.297)
Zambia	0.154(0.049-0.341)	0.041(0.012-0.094)	0.094(0.026-0.230)	0.173(0.047-0.417)	0.365(0.089-0.966)
Zimbabwe	0.143(0.049-0.310)	0.047(0.013-0.102)	0.096(0.027-0.230)	0.159(0.044-0.374)	0.306(0.079-0.802)

Supplementary Table 7: The AAPC of Incidence, Prevalence and DALYs of hepatitis B-associated cirrhosis in children and adolescents from 1990 to 2019 at the national and territorial levels

Country or region	Incidence		Prevalence		DALYs	
	AAPC (95% CI)	p value	AAPC (95% CI)	p value	AAPC (95% CI)	p value
Afghanistan	-0.08 (-0.2 to 0.03)	0.165	-2.21(-2.43 to -1.99)	<0.001	-2.73(-3.62 to -1.82)	<0.001
Albania	-1.5(-1.76 to -1.24)	<0.001	-9.41(-10.19 to -8.62)	<0.001	-3.49(-4.09 to -2.88)	<0.001
Algeria	-1.93(-2.05 to -1.81)	<0.001	-6.68(-7.46 to -5.9)	<0.001	-4.52(-4.79 to -4.25)	<0.001
American Samoa	-0.04(-0.22 to 0.15)	0.692	-0.2(-0.24 to -0.16)	<0.001	0.14(-0.12 to 0.4)	0.3
Andorra	-1.61(-1.78 to -1.44)	<0.001	-6.67(-7.15 to -6.19)	<0.001	-3.9(-4.08 to -3.73)	<0.001
Angola	-0.09(-0.36 to 0.17)	0..487	-2.18(-2.42 to -1.94)	<0.001	-2.69(-3.64 to -1.74)	<0.001
Antigua and Barbuda	-1.32(-1.45 to -1.19)	<0.001	-7.65(-8.34 to -6.96)	<0.001	-2.64(-3.55 to -1.73)	<0.001
Argentina	-1.33(-1.43 to -1.23)	<0.001	-5.54(-5.65 to -5.43)	<0.001	-3.04(-3.74 to -2.33)	<0.001
Armenia	-0.98(-1.44 to -0.52)	<0.001	-7.68(-8.19 to -7.17)	<0.001	-0.3(-1.76 to 1.18)	0.685
Australia	-2.13(-2.41 to -1.86)	<0.001	-8.22(-8.83 to -7.6)	<0.001	-3.57(-4.23 to -2.91)	<0.001
Austria	-2.13(-2.23 to -2.02)	<0.001	-6.2(-6.38 to -6.01)	<0.001	-4.06(-4.72 to -3.39)	<0.001
Azerbaijan	-0.96(-1.13 to -0.79)	<0.001	-3.3(-3.68 to -2.91)	<0.001	-1.47(-2.18 to -0.75)	<0.001
Bahamas	-1.34(-1.4 to -1.27)	<0.001	-7.12(-7.93 to -6.3)	<0.001	-3.34(-3.78 to -2.89)	<0.001
Bahrain	-1.12(-1.43 to -0.82)	<0.001	-9.03(-9.59 to -8.47)	<0.001	-3.67(-4.09 to -3.24)	<0.001
Bangladesh	-2.34(-2.57 to -2.11)	<0.001	-4.55(-4.73 to -4.37)	<0.001	-5.13(-5.84 to -4.43)	<0.001
Barbados	-1.4(-1.6 to -1.19)	<0.001	-6.13(-6.24 to -6.02)	<0.001	-3.21(-5.56 to -0.81)	0.009
Belarus	-1.11(-1.5 to -0.71)	<0.001	-9.37(-9.8 to -8.93)	<0.001	-2.61(-3.84 to -1.36)	<0.001
Belgium	-2.09(-2.23 to -1.96)	<0.001	-6.72(-6.82 to -6.63)	<0.001	-4.26(-4.99 to -3.53)	<0.001
Belize	-0.87(-1.14 to -0.6)	<0.001	-5.56(-5.98 to -5.13)	<0.001	-2.59(-3.48 to -1.69)	<0.001
Benin	-1.76(-1.97 to -1.55)	<0.001	-4.74(-5.07 to -4.4)	<0.001	-3.56(-3.83 to -3.3)	<0.001
Bermuda	0.84(0.78 to 0.91)	<0.001	-0.34(-0.42 to -0.27)	<0.001	-1.95(-2.49 to -1.41)	<0.001
Bhutan	-1.48(-1.77 to -1.19)	<0.001	-10.47(-11.14 to -9.78)	<0.001	-3.09(-3.83 to -2.33)	<0.001
Bosnia and Herzegovina	-1.88(-2.13 to -1.64)	<0.001	-4.46(-4.99 to -3.93)	<0.001	-3.24(-4.35 to -2.12)	<0.001

Botswana	-2.23(-2.89 to -1.57)	<0.001	-5.86(-6.36 to -5.36)	<0.001	-2.95(-3.84 to -2.05)	<0.001
Brazil	-4.68(-4.93 to -4.43)	<0.001	-9.36(-9.93 to -8.78)	<0.001	-5.11(-5.57 to -4.66)	<0.001
Brunei Darussalam	-2.1(-2.35 to -1.85)	<0.001	-6.98(-7.32 to -6.64)	<0.001	-4.01(-4.45 to -3.57)	<0.001
Bulgaria	-2.25(-2.4 to -2.1)	<0.001	-7.86(-8.42 to -7.3)	<0.001	-3.65(-4.65 to -2.63)	<0.001
Burkina Faso	-2.29(-3.01 to -1.57)	<0.001	-3.83(-4.5 to -3.15)	<0.001	-3.53(-4.6 to -2.44)	<0.001
Burundi	-1.72(-1.85 to -1.59)	<0.001	-6.05(-6.5 to -5.59)	<0.001	-3.89(-4.32 to -3.46)	<0.001
Cabo Verde	-2.09(-2.25 to -1.92)	<0.001	-6.72(-7.24 to -6.2)	<0.001	-2.66(-3.28 to -2.03)	<0.001
Cambodia	-1.77(-1.94 to -1.6)	<0.001	-6.46(-7.43 to -5.48)	<0.001	-3.53(-3.85 to -3.22)	<0.001
Cameroon	-1.61(-1.83 to -1.38)	<0.001	-4.12(-4.65 to -3.59)	<0.001	-2.59(-2.91 to -2.28)	<0.001
Canada	0.56(0.52 to 0.61)	<0.001	-1.77(-1.83 to -1.71)	<0.001	-1.53(-2.08 to -0.98)	<0.001
Central African Republic	-0.76(-0.91 to -0.6)	<0.001	-1.8(-1.98 to -1.61)	<0.001	-2.33(-2.72 to -1.94)	<0.001
Chad	-0.28(-0.37 to -0.19)	<0.001	-1.5(-1.66 to -1.33)	<0.001	-0.68(-1.07 to -0.3)	0.001
Chile	-1.51(-1.78 to -1.23)	<0.001	-3.01(-3.2 to -2.81)	<0.001	-4.04(-4.66 to -3.42)	<0.001
China	-4.3(-4.57 to -4.03)	<0.001	-8.43(-9.19 to -7.68)	<0.001	-6.87(-7.08 to -6.66)	<0.001
Colombia	-1.5(-1.94 to -1.06)	<0.001	-6.04(-6.56 to -5.52)	<0.001	-4.59(-5.5 to -3.66)	<0.001
Comoros	-0.79(-0.88 to -0.71)	<0.001	-4.1(-4.45 to -3.75)	<0.001	-2.26(-5.93 to 1.55)	0.241
Congo	-1.77(-2.09 to -1.46)	<0.001	-3.25(-3.58 to -2.91)	<0.001	-3.48(-4.77 to -2.17)	<0.001
Cook Islands	-1.42(-1.57 to -1.28)	<0.001	-6.46(-6.8 to -6.12)	<0.001	-4.98(-5.55 to -4.4)	<0.001
Costa Rica	-1.48(-1.65 to -1.31)	<0.001	-7.25(-7.8 to -6.71)	<0.001	-1.51(-3.51 to 0.52)	0.145
Croatia	-2.71(-2.86 to -2.57)	<0.001	-8.83(-9.66 to -7.99)	<0.001	-4.53(-5.28 to -3.79)	<0.001
Cuba	-1.52(-1.84 to -1.2)	<0.001	-7.04(-8.06 to -6.02)	<0.001	-3.9(-4.89 to -2.9)	<0.001
Cyprus	-1.49(-1.78 to -1.2)	<0.001	-8.59(-8.76 to -8.42)	<0.001	-4.09(-5.07 to -3.1)	<0.001
Czechia	-2.43(-2.58 to -2.28)	<0.001	-9.53(-10.35 to -8.71)	<0.001	-4.89(-5.88 to -3.89)	<0.001
Denmark	0.48(0.33 to 0.64)	<0.001	-0.14(-0.17 to -0.11)	<0.001	-0.75(-1.07 to -0.44)	<0.001
Djibouti	-0.9(-1.3 to -0.51)	<0.001	-2.27(-2.59 to -1.96)	<0.001	-1.44(-2.14 to -0.73)	<0.001
Dominica	-1.85(-2.45 to -1.25)	<0.001	-3.19(-3.77 to -2.61)	<0.001	-2.73(-3.85 to -1.6)	<0.001

Dominican Republic	-0.95(-1.03 to -0.87)	<0.001	-3.97(-4.22 to -3.72)	<0.001	-3.09(-3.44 to -2.73)	<0.001
Ecuador	-2.21(-2.41 to -2.02)	<0.001	-5.8(-6.09 to -5.51)	<0.001	-4.1(-4.87 to -3.33)	<0.001
Egypt	-1.8(-2.16 to -1.44)	<0.001	-7.61(-8.38 to -6.83)	<0.001	-4.47(-5.06 to -3.87)	<0.001
El Salvador	-2.11(-2.39 to -1.83)	<0.001	-7.73(-8.38 to -7.08)	<0.001	-4.88(-5.72 to -4.02)	<0.001
Equatorial Guinea	0.27(0.2 to 0.33)	<0.001	-2.61(-2.69 to -2.53)	<0.001	-2.37(-2.72 to -2.02)	<0.001
Eritrea	-0.77(-1.05 to -0.49)	<0.001	-7.9(-9.05 to -6.73)	<0.001	-2.26(-2.97 to -1.55)	<0.001
Estonia	-1.48(-1.83 to -1.12)	<0.001	-8.69(-9.32 to -8.05)	<0.001	-2.74(-3.88 to -1.6)	<0.001
Eswatini	-2.28(-2.53 to -2.02)	<0.001	-7.11(-7.55 to -6.66)	<0.001	-2.17(-2.43 to -1.92)	<0.001
Ethiopia	0.67(0.46 to 0.88)	<0.001	-2(-2.07 to -1.93)	<0.001	-1.58(-2.1 to -1.07)	<0.001
Fiji	-2.53(-2.81 to -2.25)	<0.001	-5.58(-5.85 to -5.3)	<0.001	-2.41(-2.86 to -1.95)	<0.001
Finland	0.92(0.86 to 0.98)	<0.001	-0.21(-0.29 to -0.14)	<0.001	-0.18(-0.75 to 0.4)	0.538
France	-0.98(-1.04 to -0.91)	<0.001	-3.33(-3.41 to -3.25)	<0.001	-3.68(-4.33 to -3.03)	<0.001
Gabon	-1.52(-1.62 to -1.41)	<0.001	-4.16(-4.28 to -4.05)	<0.001	-3.22(-3.71 to -2.73)	<0.001
Gambia	-1.86(-1.94 to -1.78)	<0.001	-6.67(-7.11 to -6.23)	<0.001	-2.71(-3.71 to -1.7)	<0.001
Georgia	-3.4(-3.83 to -2.96)	<0.001	-3.93(-4.09 to -3.77)	<0.001	-3.29(-5.96 to -0.54)	0.019
Germany	-1.57(-1.9 to -1.24)	<0.001	-6.18(-6.39 to -5.96)	<0.001	-3.69(-4.64 to -2.73)	<0.001
Ghana	-2.33(-2.58 to -2.08)	<0.001	-7.42(-8.29 to -6.54)	<0.001	-3.49(-3.79 to -3.19)	<0.001
Greece	-1.99(-2.22 to -1.77)	<0.001	-8.4(-8.87 to -7.93)	<0.001	-4.85(-5.62 to -4.06)	<0.001
Greenland	1.35(1.17 to 1.54)	<0.001	-0.2(-0.3 to -0.09)	<0.001	-0.84(-2.03 to 0.37)	0.175
Grenada	-1.28(-1.61 to -0.94)	<0.001	-6.58(-7.54 to -5.62)	<0.001	-2.6(-3.49 to -1.7)	<0.001
Guam	-0.21(-0.39 to -0.04)	0.0015	-0.49(-0.59 to -0.38)	<0.001	-1.38(-2.02 to -0.73)	<0.001
Guatemala	-1.72(-1.84 to -1.6)	<0.001	-2.93(-3.18 to -2.68)	<0.001	-4.16(-4.75 to -3.58)	<0.001
Guinea	-1.2(-1.3 to -1.1)	<0.001	-2.25(-2.56 to -1.94)	<0.001	-2.4(-3 to -1.79)	<0.001
Guinea-Bissau	-1.32(-1.72 to -0.93)	<0.001	-3.08(-3.5 to -2.66)	<0.001	-2.76(-3.42 to -2.08)	<0.001
Guyana	-1.95(-2.12 to -1.77)	<0.001	-7.37(-8.73 to -6)	<0.001	-3.22(-4.9 to -1.51)	<0.001
Haiti	0.43(0.37 to 0.5)	<0.001	-1.33(-1.8 to -0.85)	<0.001	-1.85(-2.17 to -1.52)	<0.001

Honduras	-3.03(-3.27 to -2.79)	<0.001	-7.01(-7.73 to -6.3)	<0.001	-5.45(-5.9 to -4.99)	<0.001
Hungary	-0.68(-0.9 to -0.45)	<0.001	-0.52(-0.58 to -0.45)	<0.001	-2.4(-3.11 to -1.69)	<0.001
Iceland	1.14(0.98 to 1.3)	<0.001	-0.47(-0.52 to -0.41)	<0.001	-0.73(-1.01 to -0.44)	<0.001
India	1.12(0.98 to 1.26)	<0.001	-1.69(-1.76 to -1.61)	<0.001	-1.15(-1.63 to -0.67)	<0.001
Indonesia	-3.81(-4.16 to -3.47)	<0.001	-2.99(-3.17 to -2.81)	<0.001	-2.22(-2.38 to -2.07)	<0.001
Iran	-1.96(-2.09 to -1.82)	<0.001	-8.91(-9.23 to -8.59)	<0.001	-4.12(-4.6 to -3.64)	<0.001
Iraq	-0.76(-0.97 to -0.54)	<0.001	-4.28(-4.58 to -3.97)	<0.001	-4.4(-4.67 to -4.13)	<0.001
Ireland	-0.15(-0.29 to -0.02)	0.026	-3.01(-3.3 to -2.72)	<0.001	-1.68(-3.64 to 0.31)	0.097
Israel	-1.66(-1.8 to -1.53)	<0.001	-7.74(-8.44 to -7.05)	<0.001	-3.95(-5.37 to -2.51)	<0.001
Italy	-3.15(-3.27 to -3.02)	<0.001	-9.14(-9.57 to -8.7)	<0.001	-5.15(-5.78 to -4.51)	<0.001
Jamaica	-1.44(-1.73 to -1.15)	<0.001	-7.01(-7.8 to -6.22)	<0.001	-4.75(-6.24 to -3.24)	<0.001
Japan	-1.2(-1.36 to -1.05)	<0.001	-0.74(-0.94 to -0.55)	<0.001	-2.81(-3.27 to -2.35)	<0.001
Jordan	-1.54(-1.89 to -1.19)	<0.001	-9.05(-9.49 to -8.6)	<0.001	-3.74(-3.96 to -3.52)	<0.001
Kazakhstan	-0.58(-0.93 to -0.23)	0.001	-8.06(-8.52 to -7.6)	<0.001	-2.86(-3.81 to -1.91)	<0.001
Kenya	-0.55(-0.68 to -0.42)	<0.001	-4.16(-4.91 to -3.4)	<0.001	-1.51(-1.81 to -1.22)	<0.001
Kiribati	-2.39(-2.6 to -2.17)	<0.001	-2.81(-3.23 to -2.37)	<0.001	-3.3(-3.55 to -3.06)	<0.001
Kuwait	-1.14(-1.35 to -0.92)	<0.001	-8.95(-9.53 to -8.37)	<0.001	-3.63(-4.81 to -2.44)	<0.001
Kyrgyzstan	-1.92(-2.28 to -1.55)	<0.001	-6.05(-6.37 to -5.73)	<0.001	-3.44(-4.3 to -2.58)	<0.001
Latvia	-1.45(-1.94 to -0.96)	<0.001	-8.68(-9.11 to -8.25)	<0.001	-2.45(-3.51 to -1.38)	<0.001
Lebanon	-0.53(-0.89 to -0.16)	0.005	-4.59(-5.24 to -3.94)	<0.001	-2.69(-3.08 to -2.29)	<0.001
Lesotho	-2.09(-2.18 to -2)	<0.001	-5.06(-5.49 to -4.63)	<0.001	-2(-2.21 to -1.8)	<0.001
Liberia	-0.43(-0.83 to -0.03)	0.036	-2.67(-2.93 to -2.41)	<0.001	-2.89(-3.85 to -1.91)	<0.001
Libya	-1.6(-1.89 to -1.31)	<0.001	-8.02(-8.64 to -7.39)	<0.001	-3.76(-4.26 to -3.26)	<0.001
Lithuania	-1.27(-1.68 to -0.85)	<0.001	-8.21(-8.77 to -7.65)	<0.001	-1.14(-2.75 to 0.5)	0.172
Luxembourg	-1.65(-1.85 to -1.45)	<0.001	-5.4(-5.68 to -5.11)	<0.001	-5.34(-6.88 to -3.77)	<0.001
Madagascar	-0.71(-0.86 to -0.57)	<0.001	-5.39(-5.94 to -4.84)	<0.001	-3.98(-4.43 to -3.52)	<0.001

Malawi	-1.3(-1.45 to -1.14)	<0.001	-5.74(-6.58 to -4.9)	<0.001	-3.68(-4.09 to -3.26)	<0.001
Malaysia	-0.57(-0.76 to -0.37)	<0.001	-6.63(-6.96 to -6.29)	<0.001	-1.5(-2.13 to -0.86)	<0.001
Maldives	-1.01(-1.14 to -0.88)	<0.001	-7.13(-7.3 to -6.96)	<0.001	-5.59(-6 to -5.17)	<0.001
Mali	-0.88(-1.01 to -0.75)	<0.001	-2.84(-3.13 to -2.55)	<0.001	-3.44(-4.02 to -2.86)	<0.001
Malta	-1.15(-1.28 to -1.02)	<0.001	-5.19(-5.56 to -4.82)	<0.001	-2.69(-3.15 to -2.23)	<0.001
Marshall Islands	-2.62(-2.86 to -2.39)	<0.001	-4.11(-4.36 to -3.85)	<0.001	-2.36(-2.6 to -2.12)	<0.001
Mauritania	-1.22(-1.26 to -1.18)	<0.001	-3.18(-3.26 to -3.1)	<0.001	-3.8(-4.08 to -3.53)	<0.001
Mauritius	-1.9(-2.15 to -1.66)	<0.001	-6.15(-6.76 to -5.55)	<0.001	-3.95(-4.73 to -3.16)	<0.001
Mexico	-3.2(-3.33 to -3.06)	<0.001	-6.19(-6.75 to -5.63)	<0.001	-2.95(-3.55 to -2.34)	<0.001
Monaco	-1.34(-1.57 to -1.11)	<0.001	-9.87(-10.55 to -9.19)	<0.001	-3.35(-3.7 to -3)	<0.001
Mongolia	-2.14(-2.41 to -1.88)	<0.001	-7.19(-7.8 to -6.57)	<0.001	-5.69(-6.56 to -4.81)	<0.001
Montenegro	-2.19(-2.76 to -1.61)	<0.001	-2.85(-3.45 to -2.25)	<0.001	-3.59(-5.64 to -1.5)	0.001
Morocco	-1.38(-1.47 to -1.3)	<0.001	-8.71(-9.14 to -8.28)	<0.001	-3.6(-4.16 to -3.05)	<0.001
Mozambique	-0.85(-0.98 to -0.73)	<0.001	-5.65(-6.12 to -5.17)	<0.001	-2.74(-3.2 to -2.28)	<0.001
Myanmar	-1.32(-1.5 to -1.14)	<0.001	-3.4(-3.78 to -3.02)	<0.001	-4.69(-5.38 to -4)	<0.001
Namibia	-1.25(-1.46 to -1.04)	<0.001	-4.28(-4.6 to -3.97)	<0.001	-1.88(-2.33 to -1.42)	<0.001
Nauru	-1.79(-2.19 to -1.39)	<0.001	-4.45(-4.7 to -4.19)	<0.001	-2.48(-2.66 to -2.31)	<0.001
Nepal	-0.95(-1.11 to -0.79)	<0.001	-4.42(-4.62 to -4.21)	<0.001	-3.22(-3.49 to -2.96)	<0.001
Netherlands	0.01(-0.17 to 0.19)	0.891	-1.61(-1.68 to -1.54)	<0.001	-2.44(-3 to -1.88)	<0.001
New Zealand	-2.57(-2.75 to -2.39)	<0.001	-5.92(-6.15 to -5.68)	<0.001	-3.67(-4.48 to -2.86)	<0.001
Nicaragua	-1.77(-1.93 to -1.61)	<0.001	-9.77(-10.2 to -9.35)	<0.001	-2.25(-2.82 to -1.67)	<0.001
Niger	-1.03(-1.35 to -0.71)	<0.001	-2.96(-3.27 to -2.66)	<0.001	-2.9(-3.43 to -2.37)	<0.001
Nigeria	-0.1(-0.13 to -0.07)	<0.001	-1.95(-2.03 to -1.88)	<0.001	-1.1(-1.37 to -0.83)	<0.001
Niue	-2.21(-2.46 to -1.96)	<0.001	-6.51(-6.88 to -6.13)	<0.001	-2.77(-3.22 to -2.31)	<0.001
North Macedonia	-1.71(-1.85 to -1.58)	<0.001	-4.73(-5.07 to -4.38)	<0.001	-3.69(-4.24 to -3.13)	<0.001
Norway	-1.84(-2.3 to -1.37)	<0.001	-6.99(-8.02 to -5.95)	<0.001	-3.55(-4.85 to -2.24)	<0.001

Oman	-1.69(-1.88 to -1.5)	<0.001	-10.15(-10.8 to -9.49)	<0.001	-3.51(-4.12 to -2.88)	<0.001
Pakistan	0.11(-0.01 to 0.23)	0.062	-4.12(-4.58 to -3.65)	<0.001	0.3(-0.02 to 0.63)	0.07
Palau	-1.95(-2.29 to -1.61)	<0.001	-5.01(-5.78 to -4.22)	<0.001	-2.58(-3.14 to -2.03)	<0.001
Palestine	-1.33(-1.55 to -1.1)	<0.001	-7.5(-8.41 to -6.58)	<0.001	-3.86(-4.42 to -3.3)	<0.001
Panama	-1.63(-1.75 to -1.51)	<0.001	-5.26(-6.09 to -4.42)	<0.001	-2.81(-3.36 to -2.27)	<0.001
Papua New Guinea	-1.63(-1.72 to -1.54)	<0.001	-3.42(-3.55 to -3.29)	<0.001	-1.96(-2.2 to -1.71)	<0.001
Paraguay	-1.78(-2.04 to -1.52)	<0.001	-6.56(-6.88 to -6.23)	<0.001	-3.84(-4.28 to -3.39)	<0.001
Peru	-1.15(-1.25 to -1.06)	<0.001	-3.64(-3.84 to -3.43)	<0.001	-4.88(-5.33 to -4.42)	<0.001
Philippines	-2.22(-2.32 to -2.12)	<0.001	-4.96(-5.44 to -4.48)	<0.001	-2.37(-2.75 to -1.99)	<0.001
Poland	-1.09(-1.71 to -0.46)	0.001	-9.69(-10.13 to -9.25)	<0.001	-4.55(-5.16 to -3.93)	<0.001
Portugal	-3.07(-3.25 to -2.89)	<0.001	-8.27(-8.55 to -7.99)	<0.001	-7.63(-8.6 to -6.64)	<0.001
Puerto Rico	-1.64(-1.69 to -1.59)	<0.001	-5.55(-5.68 to -5.42)	<0.001	-4.05(-5.27 to -2.81)	<0.001
Qatar	-2.03(-2.1 to -1.97)	<0.001	-6.35(-7.11 to -5.58)	<0.001	-4.27(-5.44 to -3.1)	<0.001
South Korea	-3.39(-3.52 to -3.27)	<0.001	-7.63(-8.1 to -7.15)	<0.001	-9.08(-10.13 to -8.02)	<0.001
Republic of Moldova	-1.8(-2.05 to -1.54)	<0.001	-4.5(-4.87 to -4.14)	<0.001	-3.7(-4.75 to -2.64)	<0.001
Romania	-2.27(-2.46 to -2.09)	<0.001	-7.75(-8.45 to -7.04)	<0.001	-5.15(-6.05 to -4.25)	<0.001
Russian Federation	-1.02(-1.26 to -0.77)	<0.001	-9.46(-10.03 to -8.88)	<0.001	-3.87(-4.7 to -3.04)	<0.001
Rwanda	-1.8(-2.11 to -1.49)	<0.001	-6.48(-7.04 to -5.92)	<0.001	-4.4(-5.02 to -3.77)	<0.001
Saint Kitts and Nevis	-1.59(-1.96 to -1.23)	<0.001	-7.8(-8.46 to -7.12)	<0.001	-3.15(-4.99 to -1.28)	0.001
Saint Lucia	-1.05(-1.23 to -0.88)	<0.001	-6.34(-6.59 to -6.09)	<0.001	-2.96(-4.7 to -1.19)	0.001
Samoa	-1.05(-1.16 to -0.94)	<0.001	-1.23(-1.57 to -0.9)	<0.001	-2.4(-2.54 to -2.27)	<0.001
San Marino	-1.47(-1.57 to -1.38)	<0.001	-5.73(-6.41 to -5.04)	<0.001	-3.24(-3.5 to -2.97)	<0.001
Sao Tome and Principe	-2.19(-2.32 to -2.05)	<0.001	-6.2(-6.81 to -5.59)	<0.001	-3.78(-6.28 to -1.21)	0.004
Saudi Arabia	-1.5(-1.67 to -1.33)	<0.001	-10.11(-10.41 to -9.81)	<0.001	-5.13(-5.34 to -4.92)	<0.001
Senegal	-1.58(-1.82 to -1.33)	<0.001	-5.3(-5.89 to -4.72)	<0.001	-3.26(-4.3 to -2.21)	<0.001
Serbia	-1.97(-2.09 to -1.86)	<0.001	-5.08(-5.58 to -4.58)	<0.001	-5.73(-6.58 to -4.88)	<0.001

Seychelles	-1.46(-1.6 to -1.31)	<0.001	-6.16(-7.01 to -5.3)	<0.001	-2.5(-3.72 to -1.27)	<0.001
Sierra Leone	-0.97(-1.77 to -0.17)	0.018	-3.94(-4.37 to -3.5)	<0.001	-3.7(-5.08 to -2.3)	<0.001
Singapore	-1.86(-2.23 to -1.5)	<0.001	-7.96(-8.41 to -7.51)	<0.001	-5.83(-6.74 to -4.9)	<0.001
Slovakia	-2.09(-2.51 to -1.66)	<0.001	-9.71(-10.36 to -9.06)	<0.001	-3.92(-4.41 to -3.43)	<0.001
Slovenia	-2.72(-2.9 to -2.53)	<0.001	-7.85(-8.49 to -7.2)	<0.001	-3.83(-4.41 to -3.24)	<0.001
Solomon Islands	-2.78(-3.22 to -2.34)	<0.001	-4.92(-5.27 to -4.57)	<0.001	-3.26(-3.67 to -2.84)	<0.001
Somalia	0.59(0.51 to 0.66)	<0.001	-0.98(-1.17 to -0.79)	<0.001	0.29(-0.04 to 0.63)	0.088
South Africa	-2.7(-2.81 to -2.6)	<0.001	-3.93(-4.25 to -3.62)	<0.001	-4.49(-5.07 to -3.91)	<0.001
South Sudan	1.06(0.94 to 1.19)	<0.001	-1.07(-1.26 to -0.89)	<0.001	0.98(-0.09 to 2.07)	0.072
Spain	-3.24(-3.56 to -2.93)	<0.001	-8.37(-8.79 to -7.95)	<0.001	-6.83(-7.47 to -6.18)	<0.001
Sri Lanka	-2.5(-2.7 to -2.3)	<0.001	-6.68(-7.36 to -6)	<0.001	-5.74(-6.74 to -4.72)	<0.001
Sudan	-0.61(-0.77 to -0.45)	<0.001	-4.21(-4.44 to -3.97)	<0.001	-2.97(-3.34 to -2.59)	<0.001
Suriname	-1.84(-1.91 to -1.77)	<0.001	-6.09(-6.66 to -5.52)	<0.001	-3.95(-5.21 to -2.67)	<0.001
Sweden	0.11(0.02 to 0.19)	0.014	-0.81(-0.95 to -0.67)	<0.001	-0.69(-1.34 to -0.04)	0.038
Switzerland	0.37(0.23 to 0.51)	<0.001	-0.78(-0.94 to -0.62)	<0.001	-1.99(-2.61 to -1.37)	<0.001
Syrian Arab Republic	-1.35(-1.54 to -1.15)	<0.001	-5.15(-5.68 to -4.62)	<0.001	-2.53(-3.26 to -1.79)	<0.001
Tajikistan	-1.05(-1.25 to -0.85)	<0.001	-6.06(-6.42 to -5.7)	<0.001	-2.57(-3.75 to -1.38)	<0.001
Thailand	-2.32(-2.69 to -1.94)	<0.001	-7.37(-7.54 to -7.2)	<0.001	-5.11(-5.64 to -4.58)	<0.001
Timor-Leste	-0.53(-0.84 to -0.21)	0.001	-2.69(-3.01 to -2.37)	<0.001	-1.96(-3.04 to -0.86)	0.001
Togo	-1.23(-1.35 to -1.1)	<0.001	-3.45(-3.81 to -3.1)	<0.001	-3.16(-3.69 to -2.62)	<0.001
Tokelau	-0.12(-0.18 to -0.06)	<0.001	-0.46(-0.61 to -0.32)	<0.001	-1.34(-1.52 to -1.16)	<0.001
Tonga	-2.77(-3 to -2.53)	<0.001	-7.37(-8.05 to -6.68)	<0.001	-2.94(-3.3 to -2.58)	<0.001
Trinidad and Tobago	-1.39(-1.61 to -1.17)	<0.001	-4.32(-4.55 to -4.1)	<0.001	-3.48(-3.96 to -2.99)	<0.001
Tunisia	-1.09(-1.3 to -0.88)	<0.001	-8.32(-8.86 to -7.78)	<0.001	-3.83(-4.01 to -3.65)	<0.001
Turkey	-0.49(-0.68 to -0.29)	<0.001	-7.56(-7.75 to -7.37)	<0.001	-5(-5.96 to -4.02)	<0.001
Turkmenistan	-0.08(-0.65 to 0.5)	0.792	-6.93(-7.7 to -6.14)	<0.001	-0.83(-1.96 to 0.31)	0.153

Tuvalu	-1.64(-2.12 to -1.16)	<0.001	-2.66(-2.93 to -2.4)	<0.001	-3.14(-3.42 to -2.86)	<0.001
Uganda	-0.92(-1.02 to -0.82)	<0.001	-5.57(-5.89 to -5.25)	<0.001	-1.55(-2.18 to -0.91)	<0.001
Ukraine	0.64(0.41 to 0.87)	<0.001	-4.77(-5.17 to -4.36)	<0.001	-0.59(-2.36 to 1.22)	0.521
United Arab Emirates	-0.57(-1.08 to -0.05)	0.031	-6.01(-6.8 to -5.21)	<0.001	-2.6(-3.27 to -1.92)	<0.001
United Kingdom	-0.27(-0.38 to -0.16)	<0.001	-0.64(-0.77 to -0.51)	<0.001	0.12(-0.46 to 0.71)	0.678
United States of America	-2.98(-3.17 to -2.79)	<0.001	-5.98(-6.37 to -5.58)	<0.001	-3.49(-3.91 to -3.07)	<0.001
Uruguay	-1.34(-1.58 to -1.11)	<0.001	-7.03(-7.75 to -6.3)	<0.001	-4.07(-5.68 to -2.43)	<0.001
Uzbekistan	-1.44(-1.68 to -1.2)	<0.001	-6.62(-6.84 to -6.39)	<0.001	-2.07(-2.45 to -1.68)	<0.001
Vanuatu	-2.87(-3.36 to -2.38)	<0.001	-3.67(-4.69 to -2.64)	<0.001	-2.47(-2.88 to -2.06)	<0.001
Venezuela	-1.3(-1.82 to -0.78)	<0.001	-3.61(-3.83 to -3.39)	<0.001	-2.48(-4.37 to -0.55)	0.012
Viet Nam	-1.45(-1.73 to -1.18)	<0.001	-6.23(-6.47 to -5.99)	<0.001	-3.23(-3.54 to -2.93)	<0.001
Yemen	-0.84(-1 to -0.69)	<0.001	-4.3(-4.52 to -4.07)	<0.001	-2.41(-2.73 to -2.08)	<0.001
Zambia	-1.52(-1.62 to -1.42)	<0.001	-3.98(-4.23 to -3.73)	<0.001	-2.39(-2.79 to -1.99)	<0.001
Zimbabwe	-2.43(-2.52 to -2.34)	<0.001	-4.68(-4.83 to -4.54)	<0.001	-2.5(-3.77 to -1.22)	<0.001

Supplementary Table 8: The prevalence of hepatitis B-associated cirrhosis in children and adolescents from 1990 to 2019 at the country and regional levels

Location	<20 years	<5 years	5-9 years	10-14 years	15-19 years
Afghanistan	3997.63(2959.48-5071.63)	1659.99(1159.82-2199.80)	3152.76(2263.94-4113.34)	4667.83(3439.16-5971.04)	7931.38(6018.60-10016.40)
Albania	48.35(36.36-61.77)	32.03(22.62-42.67)	49.21(35.82-64.98)	49.80(37.61-63.76)	60.25(47.39-74.91)
Algeria	473.76(356.90-605.10)	272.78(190.61-366.87)	406.95(297.61-534.37)	395.53(297.49-506.07)	933.66(721.21-1166.34)
American Samoa	4024.34(3066.03-4989.78)	2572.96(1801.95-3364.20)	3837.70(2838.51-4913.23)	4485.63(3411.10-5626.50)	4974.65(3939.10-6094.59)
Andorra	132.08(97.87-169.41)	40.87(27.96-56.69)	83.13(58.93-112.23)	100.24(73.11-129.66)	266.67(201.98-338.04)
Angola	9378.13(7793.78-11099.94)	6817.42(5185.75-8565.36)	8736.59(7176.23-10465.58)	9004.62(7557.67-10617.16)	15172.42(12866.24-17659.18)
Antigua and Barbuda	51.44(39.13-65.21)	30.45(21.94-41.49)	46.27(33.75-61.26)	60.08(45.24-76.89)	63.44(49.49-80.16)
Argentina	54.45(40.36-69.59)	21.68(13.72-29.51)	37.12(25.45-49.89)	47.56(35.15-61.76)	110.49(86.20-139.48)
Armenia	252.61(192.25-308.95)	156.12(112.70-206.79)	250.23(187.80-309.64)	358.48(272.46-438.32)	253.67(195.81-305.13)
Australia	144.20(124.41-167.24)	72.46(57.01-89.64)	142.60(118.96-170.01)	168.99(146.03-196.28)	196.06(173.94-222.85)
Austria	123.88(92.95-160.57)	56.10(37.41-76.97)	66.50(48.11-91.22)	168.71(125.92-218.71)	201.08(155.04-257.52)
Azerbaijan	1255.38(1008.31-1516.49)	564.74(410.07-722.04)	771.15(599.19-958.82)	1348.29(1078.30-1635.52)	2530.47(2065.10-3009.36)
Bahamas	70.15(53.33-87.23)	29.83(20.62-40.33)	49.65(35.40-64.12)	80.56(60.96-100.42)	105.86(83.25-128.17)
Bahrain	181.82(139.61-230.60)	118.82(85.02-158.34)	162.36(122.46-209.11)	194.82(148.69-247.63)	239.51(188.12-298.09)
Bangladesh	590.96(520.47-667.19)	128.66(102.10-156.36)	213.64(180.27-249.65)	263.57(229.87-299.72)	1702.06(1510.85-1912.92)
Barbados	74.40(55.37-94.25)	34.09(24.08-45.93)	59.56(42.86-77.10)	92.97(68.88-118.68)	98.98(76.04-124.40)
Belarus	54.19(44.34-64.06)	40.71(31.87-49.65)	49.50(39.62-59.17)	60.08(48.95-70.90)	70.74(58.55-83.00)
Belgium	69.32(57.84-84.06)	15.90(11.96-20.77)	29.14(23.21-37.56)	44.91(36.99-55.48)	190.18(161.00-227.88)
Belize	144.81(108.29-179.53)	66.32(46.25-87.61)	124.94(89.12-158.79)	161.76(120.52-203.90)	211.97(163.68-262.55)
Benin	3370.09(2451.20-4110.65)	2787.26(1840.70-3633.49)	3440.97(2442.89-4248.83)	2322.29(1734.24-2808.79)	5491.93(4278.93-6591.68)
Bermuda	389.57(285.34-504.52)	235.88(160.91-321.34)	375.27(266.33-503.65)	438.91(321.84-570.35)	481.90(365.78-605.26)
Bhutan	210.10(191.90-224.06)	156.44(133.20-174.18)	197.89(177.54-213.93)	234.42(216.68-249.54)	247.73(231.96-263.11)
Bolivia	69.92(56.59-86.63)	26.64(19.86-35.35)	60.51(47.12-78.82)	82.68(67.24-101.78)	129.40(107.98-155.76)
Bosnia and Herzegovina	136.72(105.68-168.18)	78.99(55.21-104.83)	101.01(74.67-129.79)	97.44(75.41-122.28)	247.97(199.15-302.17)

Botswana	1381.28(1047.98-1775.39)	1388.58(980.34-1844.23)	1221.71(919.64-1600.16)	1384.71(1059.90-1774.83)	1548.39(1193.02-1959.98)
Brazil	236.69(192.92-289.18)	124.52(97.56-156.73)	199.52(157.68-254.90)	260.77(212.21-321.50)	354.48(294.87-424.98)
Brunei Darussalam	352.40(288.65-427.92)	199.18(152.03-247.85)	310.78(250.09-385.76)	414.02(338.38-505.16)	467.64(388.24-558.09)
Bulgaria	341.48(310.64-367.48)	271.59(219.53-324.18)	440.35(388.41-486.29)	317.50(296.57-336.76)	332.37(316.46-349.65)
Burkina Faso	5715.80(4265.29-6995.09)	1811.23(1222.24-2344.35)	2403.91(1698.42-3009.80)	3056.94(2240.67-3769.97)	20407.48(15553.39-24807.73)
Burundi	514.15(478.59-548.51)	182.84(149.99-216.34)	240.09(213.72-265.35)	344.80(319.59-369.25)	1657.08(1576.37-1735.63)
Cabo Verde	1438.56(1114.70-1739.29)	682.39(508.92-867.65)	883.05(666.39-1080.17)	745.81(574.61-908.84)	3559.78(2738.14-4305.76)
Cambodia	1300.76(1013.25-1592.67)	546.79(403.63-718.82)	1035.98(778.37-1326.81)	1432.57(1112.72-1765.00)	2359.76(1891.49-2823.47)
Cameroon	1859.03(1602.01-2093.66)	1024.29(840.87-1222.30)	1549.68(1311.61-1782.60)	1930.27(1667.63-2169.52)	3260.37(2851.65-3617.15)
Canada	817.02(681.45-987.77)	265.39(204.24-338.72)	523.63(419.34-660.07)	1055.69(873.57-1280.08)	1394.78(1194.99-1647.53)
Central African Republic	6267.88(5909.67-6648.49)	3311.52(2937.52-3745.72)	6295.36(5823.03-6805.73)	5420.62(5136.81-5737.72)	11438.78(10927.65-12015.71)
Chad	10382.07(7762.72-12612.92)	7875.83(5664.38-10013.87)	9333.09(6851.93-11486.36)	11239.92(8471.86-13678.37)	15622.48(12076.01-18868.28)
Chile	142.06(107.37-179.43)	17.54(11.09-25.99)	37.00(25.43-51.42)	49.86(36.56-65.18)	456.27(349.62-571.88)
China	904.29(764.33-1065.38)	420.77(341.40-506.45)	566.55(470.33-686.01)	805.46(676.38-964.81)	1848.12(1565.03-2176.26)
Colombia	534.77(402.46-682.85)	259.77(181.75-348.72)	471.48(337.06-634.02)	553.05(412.78-708.32)	836.82(647.24-1046.18)
Comoros	1639.50(1212.53-2098.60)	697.41(486.71-951.35)	1360.84(976.19-1794.68)	2046.12(1512.40-2636.50)	2528.83(1930.55-3238.62)
Congo	4810.14(3728.75-5854.53)	2569.04(1885.69-3352.75)	3300.89(2485.25-4103.16)	4557.52(3537.55-5608.38)	10171.51(8012.97-12438.67)
Cook Islands	501.70(375.67-641.92)	246.56(166.56-331.87)	446.42(322.81-597.38)	737.79(556.37-951.44)	554.62(429.46-699.05)
Costa Rica	92.51(69.09-118.69)	23.52(16.25-32.11)	49.46(35.19-66.38)	133.64(98.73-172.97)	158.59(120.47-200.40)
Croatia	104.11(87.18-121.51)	87.43(69.80-106.79)	111.74(92.09-134.22)	106.64(89.73-124.27)	108.65(93.57-124.37)
Cuba	51.97(38.96-64.34)	21.65(15.23-28.55)	34.52(24.90-44.74)	55.79(41.39-69.99)	89.88(68.89-110.35)
Cyprus	53.74(49.43-57.72)	18.63(14.44-23.06)	36.22(31.81-40.41)	58.36(53.98-62.53)	107.88(102.33-113.74)
Czechia	52.39(40.60-63.47)	29.80(21.07-39.46)	41.84(30.72-53.22)	45.27(35.04-55.18)	100.64(80.87-120.51)
C d'Ivoire	2545.67(2407.47-2677.15)	1247.61(1077.45-1395.97)	2146.83(1981.57-2304.58)	3603.22(3457.03-3764.69)	3821.13(3717.69-3919.62)
Korea	1968.94(1607.05-2335.98)	611.60(465.87-783.58)	987.83(781.87-1213.46)	1485.81(1201.52-1769.24)	4524.80(3734.72-5357.63)

Democratic Republic of the Congo	2663.37(2073.44-3305.44)	1226.94(883.33-1586.42)	1831.94(1367.23-2338.32)	2511.53(1946.34-3131.02)	5991.81(4772.94-7347.35)
Denmark	700.04(532.87-908.13)	371.94(244.36-517.31)	681.56(492.66-921.84)	815.59(623.23-1063.79)	897.61(707.59-1127.90)
Djibouti	3172.20(2309.39-4160.38)	1795.22(1238.45-2496.17)	2643.68(1859.53-3576.76)	2594.87(1874.95-3370.45)	6663.26(4999.26-8488.55)
Dominica	229.64(165.58-284.53)	36.69(24.83-48.77)	55.73(37.82-72.28)	75.38(53.06-94.67)	662.94(480.91-818.65)
Dominican Republic	267.92(207.74-325.91)	175.16(126.02-224.24)	283.63(212.15-348.51)	321.46(250.69-390.44)	304.01(243.75-366.82)
Ecuador	65.03(52.00-80.38)	40.98(29.24-55.25)	66.49(51.38-85.10)	54.84(44.52-67.14)	98.59(82.78-118.00)
Egypt	308.16(280.46-336.64)	105.05(86.04-125.61)	240.19(213.30-268.35)	308.63(284.07-334.90)	629.22(586.01-674.24)
El Salvador	96.89(71.49-124.38)	70.05(47.53-96.20)	102.31(72.88-135.32)	82.55(61.18-106.81)	131.29(100.30-165.77)
Equatorial Guinea	971.38(790.88-1152.80)	484.35(360.06-624.22)	628.51(494.51-768.77)	1324.50(1085.06-1568.63)	1449.72(1228.37-1674.87)
Eritrea	760.41(564.74-982.29)	416.40(293.17-568.01)	694.56(502.71-928.93)	892.16(664.37-1152.17)	1114.06(850.03-1405.28)
Estonia	51.65(38.62-66.62)	33.23(23.24-44.88)	42.10(30.38-56.42)	52.52(39.37-68.07)	82.95(64.01-104.08)
Eswatini	1238.61(946.96-1552.29)	1147.66(834.94-1513.53)	1283.82(975.99-1658.70)	1102.58(843.71-1395.03)	1439.00(1106.92-1790.96)
Ethiopia	2859.12(2368.07-3445.06)	1321.18(1056.61-1630.89)	2298.95(1853.98-2893.98)	3277.54(2700.30-4016.63)	5113.30(4288.04-6112.75)
Fiji	1016.00(820.06-1175.91)	466.64(337.57-583.97)	765.78(589.22-913.22)	1214.00(980.95-1419.21)	1733.06(1455.00-1999.44)
Finland	704.26(534.59-901.61)	375.95(254.63-516.06)	680.36(493.75-922.15)	816.43(620.46-1037.89)	901.65(706.47-1105.54)
France	422.10(378.27-470.30)	71.49(54.75-86.92)	200.58(168.64-233.93)	521.78(461.92-585.97)	849.17(778.43-930.28)
Gabon	2630.56(2275.77-3087.67)	1205.71(949.87-1512.82)	1741.76(1460.67-2105.87)	2847.82(2446.71-3343.79)	4992.92(4389.95-5738.88)
Gambia	726.24(652.44-798.07)	475.93(388.33-560.16)	628.98(549.13-703.19)	858.09(783.26-931.38)	1013.07(949.06-1076.53)
Georgia	561.89(535.13-589.22)	242.89(208.61-276.63)	324.04(298.43-349.59)	745.74(715.07-778.22)	1071.32(1042.66-1099.01)
Germany	45.47(35.46-58.94)	15.65(10.18-22.64)	36.34(26.56-50.59)	46.82(36.30-60.91)	81.29(66.09-101.21)
Ghana	1932.40(1680.38-2228.26)	639.38(495.32-796.73)	1627.87(1352.54-1947.12)	2408.76(2109.33-2776.23)	3326.72(2969.69-3771.87)
Greece	118.40(97.69-141.64)	50.57(37.39-65.87)	94.40(74.76-118.50)	135.29(111.81-162.88)	183.43(157.17-214.00)
Greenland	1024.19(760.22-1360.17)	578.39(395.16-805.04)	1016.87(726.51-1417.98)	1211.64(899.60-1586.66)	1332.04(1003.49-1683.83)
Grenada	102.48(76.88-125.92)	60.42(42.12-80.60)	79.40(57.86-100.86)	87.39(65.38-107.73)	170.37(131.35-207.21)
Guam	3314.06(2485.34-4271.13)	2083.08(1410.92-2838.19)	3200.49(2319.74-4285.94)	3840.65(2934.84-4882.19)	4348.15(3415.98-5422.83)

Guatemala	551.70(412.01-695.00)	162.38(111.15-216.33)	391.61(280.66-521.45)	252.48(185.95-323.38)	1422.05(1084.94-1762.89)
Guinea	8322.47(6093.55-9968.26)	6357.74(4256.79-8060.37)	7336.67(5270.59-8859.75)	6516.34(4841.71-7732.94)	15166.31(11617.58-17943.44)
Guinea-Bissau	6301.92(4950.54-7460.31)	3768.09(2776.23-4696.89)	3869.77(2939.27-4644.85)	4267.59(3330.98-5116.97)	15342.38(12298.58-18187.23)
Guyana	104.20(78.10-128.22)	49.48(34.00-66.67)	89.20(64.67-113.14)	114.48(86.64-141.40)	161.95(126.06-195.84)
Haiti	1152.36(919.10-1393.06)	314.86(234.77-412.66)	818.46(638.90-1017.32)	1778.36(1413.23-2169.82)	1896.34(1517.87-2280.60)
Honduras	174.20(131.49-230.14)	118.68(83.06-160.54)	204.94(150.53-282.72)	147.38(111.61-194.45)	228.58(176.73-291.05)
Hungary	456.56(380.00-552.62)	222.20(159.28-304.73)	408.20(323.76-519.64)	532.30(448.41-641.31)	637.71(560.81-742.04)
Iceland	534.72(394.08-694.66)	299.30(199.82-414.03)	533.06(376.39-719.26)	625.64(466.14-811.31)	672.54(519.88-852.84)
India	1935.19(1626.19-2299.71)	517.87(419.18-631.76)	1062.95(873.87-1290.08)	2612.37(2190.46-3135.33)	3312.60(2809.15-3928.06)
Indonesia	1593.08(1330.09-1898.00)	615.21(491.65-758.02)	1341.09(1091.81-1639.98)	2200.90(1831.22-2635.53)	2057.11(1736.90-2436.74)
Iran	145.76(121.77-173.48)	86.89(69.68-105.31)	131.89(107.61-161.24)	157.35(130.57-189.52)	226.19(189.66-267.61)
Iraq	933.74(726.89-1147.68)	426.75(314.75-558.28)	1044.16(797.06-1319.89)	968.27(746.78-1194.49)	1317.59(1031.63-1601.43)
Ireland	277.92(206.20-370.31)	30.45(20.19-44.17)	71.93(49.78-102.97)	311.98(227.84-427.59)	719.07(537.60-927.92)
Israel	65.87(47.88-86.57)	36.18(24.75-49.89)	72.83(51.45-98.25)	78.65(57.89-102.15)	82.63(62.49-104.83)
Italy	49.01(39.83-59.02)	26.60(20.62-33.20)	47.94(37.70-59.66)	56.12(45.75-68.05)	61.27(50.96-73.17)
Jamaica	46.70(37.02-58.26)	13.36(9.50-17.59)	27.06(20.13-35.29)	42.22(32.92-53.87)	92.68(74.79-114.93)
Japan	1850.52(1527.58-2272.06)	82.42(64.57-104.09)	1971.88(1588.28-2505.51)	2387.95(1964.51-2946.77)	2661.16(2227.84-3204.07)
Jordan	542.45(522.16-560.18)	531.36(497.40-564.50)	720.86(679.24-754.75)	459.30(441.60-475.69)	452.34(435.71-470.32)
Kazakhstan	313.70(243.09-379.09)	220.67(170.87-278.38)	326.59(250.96-402.77)	289.59(222.98-348.07)	468.48(367.16-555.59)
Kenya	923.91(765.61-1130.32)	536.38(427.09-664.46)	787.68(632.43-988.18)	1054.66(876.85-1299.16)	1356.25(1141.97-1618.14)
Kiribati	3787.15(2720.03-4715.58)	2752.57(1775.06-3638.65)	3723.37(2601.40-4701.50)	3964.40(2935.29-4882.41)	5027.40(3834.51-6099.12)
Kuwait	104.65(79.43-130.11)	66.62(47.36-88.81)	99.80(74.60-125.94)	123.90(94.83-154.84)	137.03(106.19-170.36)
Kyrgyzstan	625.57(499.36-758.77)	642.91(497.23-809.95)	731.79(581.41-900.42)	539.24(433.86-647.78)	550.83(446.80-652.48)
Lao People's Republic	4488.68(3575.00-5491.71)	2110.36(1561.17-2692.25)	3091.33(2414.99-3836.19)	4875.63(3894.66-5946.99)	8252.85(6698.76-9983.20)
Latvia	74.12(61.19-86.60)	51.18(41.22-61.64)	65.69(53.73-77.56)	112.12(92.79-130.74)	67.69(57.18-78.31)
Lebanon	944.21(738.99-1163.76)	581.56(434.41-761.75)	852.51(656.02-1073.32)	1232.95(969.04-1518.12)	1263.78(1018.37-1520.67)

Lesotho	2448.08(1903.30-3074.57)	587.67(427.26-768.72)	1496.05(1143.59-1937.87)	2135.65(1647.03-2709.67)	5560.34(4318.80-6932.36)
Liberia	6056.56(4582.64-7354.73)	2202.78(1546.52-2796.33)	4436.30(3223.14-5446.91)	5044.81(3802.13-6145.65)	13739.28(10610.06-16851.09)
Libya	197.30(157.06-238.98)	133.64(97.47-181.08)	237.46(183.64-298.16)	182.81(147.01-219.25)	223.64(183.88-262.45)
Lithuania	60.21(45.15-73.11)	30.18(21.49-39.27)	45.93(33.30-57.06)	58.32(43.70-71.44)	107.75(83.41-130.23)
Luxembourg	135.27(98.19-179.04)	88.29(58.36-125.60)	98.77(68.82-138.70)	102.83(74.75-137.35)	248.98(189.20-318.21)
Madagascar	1552.16(1207.51-1902.80)	850.28(599.83-1126.57)	1486.63(1105.09-1873.96)	1864.96(1470.46-2296.01)	2219.67(1817.00-2653.31)
Malawi	2220.96(1724.97-2718.96)	1594.44(1168.67-2140.26)	1935.80(1475.39-2404.73)	2321.81(1800.28-2827.77)	3144.25(2475.31-3809.24)
Malaysia	182.48(152.89-216.48)	90.56(68.39-116.94)	173.58(140.85-214.48)	208.08(176.63-247.40)	253.38(219.54-294.36)
Maldives	520.61(381.46-651.72)	477.73(337.35-639.52)	737.87(539.03-937.96)	421.74(313.58-519.32)	415.68(315.26-505.04)
Mali	2844.49(1852.29-3591.54)	2328.88(1358.40-3062.14)	3092.32(1963.97-3984.48)	2979.07(2015.31-3729.19)	3199.40(2294.34-3972.40)
Malta	148.76(105.42-193.37)	58.74(37.60-79.32)	136.24(90.44-182.18)	208.61(148.02-272.73)	198.21(149.05-255.48)
Marshall Islands	1319.28(1039.66-1630.91)	742.05(531.91-992.77)	1340.45(1034.27-1713.02)	1006.54(796.04-1251.06)	2284.66(1844.39-2781.77)
Mauritania	6944.16(6697.91-7156.42)	3364.46(2992.31-3683.76)	4958.31(4672.48-5221.92)	6403.77(6224.57-6577.51)	14254.44(13972.56-14535.83)
Mauritius	523.08(393.92-655.65)	423.00(296.56-558.78)	619.23(453.47-806.84)	487.22(369.60-607.51)	551.64(432.42-665.93)
Mexico	33.14(26.57-40.72)	17.67(12.50-22.80)	39.52(30.76-49.95)	34.59(28.23-42.49)	40.18(33.99-48.01)
Micronesia	1125.12(902.40-1388.66)	818.68(618.70-1083.51)	1203.26(951.00-1524.09)	1239.82(1006.15-1520.00)	1204.77(995.66-1440.99)
Monaco	33.52(25.03-43.96)	17.69(12.06-24.19)	32.20(23.12-43.94)	39.09(29.31-51.16)	43.72(33.52-55.50)
Mongolia	597.89(477.59-705.37)	285.20(197.80-360.68)	426.52(326.37-518.79)	755.83(611.37-892.55)	1244.79(1043.48-1438.09)
Montenegro	287.71(221.65-349.55)	87.49(61.11-116.43)	134.71(97.96-173.51)	116.43(87.73-143.71)	755.08(591.20-910.29)
Morocco	198.96(183.16-214.54)	134.54(116.66-151.69)	188.65(171.62-205.64)	197.32(181.58-213.30)	274.33(254.43-293.85)
Mozambique	2915.22(2270.93-3528.15)	848.36(620.65-1092.01)	2796.07(2117.91-3424.79)	3956.48(3065.83-4842.55)	5072.83(4007.85-6162.40)
Myanmar	1041.87(822.14-1275.35)	611.61(458.79-818.69)	1035.48(798.58-1303.69)	901.22(713.85-1101.19)	1625.11(1317.70-1935.11)
Namibia	1841.00(1791.86-1878.11)	426.77(384.44-464.27)	743.77(706.17-771.41)	1347.37(1305.56-1378.55)	5403.45(5289.05-5504.64)
Nauru	1503.60(1125.45-1915.54)	526.78(364.40-692.74)	2143.11(1557.42-2791.31)	1166.62(875.77-1492.92)	2346.34(1825.96-2936.00)
Nepal	369.72(312.92-424.95)	145.51(109.44-184.30)	210.98(170.22-251.50)	254.66(214.33-294.27)	830.33(717.22-941.55)
Netherlands	463.82(345.58-598.93)	55.29(37.72-73.40)	197.02(139.85-265.04)	721.40(532.91-948.67)	808.54(616.17-1045.01)

New Zealand	147.77(121.77-180.72)	66.30(52.06-83.77)	125.46(100.10-159.80)	168.56(138.44-206.94)	230.83(192.11-276.53)
Nicaragua	62.96(61.06-65.08)	28.72(26.85-30.79)	45.15(42.97-47.39)	54.45(52.51-56.64)	127.18(122.86-132.42)
Niger	5400.46(4956.67-5785.07)	3286.49(2772.74-3724.51)	4015.94(3606.90-4406.20)	4699.59(4357.48-5000.66)	12499.39(11912.45-13049.71)
Nigeria	7909.87(6681.00-9390.68)	6581.23(5377.45-7980.32)	8036.20(6638.29-9669.52)	7894.86(6644.22-9377.91)	9614.72(8126.93-11353.89)
Niue	542.36(399.34-715.20)	290.21(195.22-397.70)	481.16(340.76-655.33)	710.67(522.78-939.20)	659.68(500.37-848.00)
North Macedonia	196.69(152.31-237.67)	75.97(53.70-102.07)	111.35(81.48-141.98)	147.01(112.21-181.00)	437.63(343.81-525.02)
Northern Mariana Islands	4543.89(3496.60-5728.74)	2552.14(1779.97-3519.34)	4061.05(3013.83-5361.11)	4920.45(3772.00-6215.94)	5615.19(4448.03-6923.22)
Norway	88.72(70.66-108.77)	33.31(25.02-42.43)	70.84(54.54-89.89)	98.10(77.46-121.31)	148.23(119.03-179.31)
Oman	139.30(115.49-164.73)	85.45(65.28-111.07)	149.02(122.26-178.29)	166.36(138.62-193.56)	178.47(149.88-206.82)
Pakistan	979.24(822.29-1179.58)	542.99(429.02-677.13)	864.26(706.94-1057.50)	1189.53(995.24-1435.19)	1418.87(1214.08-1684.96)
Palau	949.42(716.16-1209.00)	489.51(333.19-650.10)	885.09(640.65-1165.86)	1591.76(1211.47-2030.53)	721.89(566.16-903.48)
Palestine	393.95(307.32-505.06)	182.78(131.75-241.31)	343.95(260.68-454.83)	613.93(477.65-790.70)	458.07(362.92-572.82)
Panama	156.87(112.62-199.26)	79.29(53.82-107.31)	149.14(103.48-196.73)	176.98(127.26-224.40)	229.87(170.80-288.79)
Papua New Guinea	3005.23(2513.74-3434.02)	2569.88(1892.18-3145.94)	3053.65(2501.15-3510.31)	3247.84(2841.61-3601.81)	3341.35(3046.82-3616.34)
Paraguay	433.03(322.07-567.87)	140.44(97.11-188.35)	338.29(237.29-464.61)	530.75(391.39-705.76)	718.08(549.10-924.31)
Peru	119.50(104.00-138.46)	42.50(32.39-54.59)	72.63(60.75-86.28)	129.48(112.94-151.87)	243.14(216.69-280.38)
Philippines	3039.75(2535.59-3650.12)	1163.86(953.03-1420.37)	2235.85(1827.99-2758.44)	3490.84(2899.74-4219.12)	5664.15(4746.58-6769.93)
Poland	34.52(27.78-42.42)	21.65(16.51-27.23)	37.35(29.25-47.51)	37.21(30.08-45.80)	41.99(34.81-50.44)
Portugal	87.94(64.60-112.82)	36.37(24.22-49.56)	68.11(47.09-92.17)	95.57(69.68-122.04)	133.74(101.97-166.89)
Puerto Rico	73.26(57.46-95.61)	37.40(26.94-51.67)	62.52(47.18-85.30)	76.07(59.23-99.58)	99.41(78.63-125.11)
Qatar	157.03(122.40-192.44)	95.52(66.51-127.17)	174.04(131.69-218.27)	161.13(128.01-197.37)	210.08(174.01-251.49)
South Korea	357.76(310.73-423.56)	103.51(78.02-133.35)	167.27(135.09-211.46)	365.89(313.20-438.89)	725.94(646.84-838.33)
Republic of Moldova	322.50(252.35-394.30)	329.01(248.55-422.93)	430.59(334.34-530.25)	363.24(288.05-441.33)	164.02(131.82-195.91)
Romania	121.01(94.34-152.47)	139.51(103.25-183.75)	153.70(119.02-199.37)	117.73(93.64-146.50)	76.17(60.80-93.10)
Russian Federation	55.79(46.40-66.48)	34.24(27.16-41.23)	46.44(37.72-57.05)	55.68(46.24-67.26)	94.67(79.51-112.02)

Rwanda	978.68(752.53-1223.84)	353.42(257.53-472.88)	564.86(425.77-723.11)	1251.18(962.81-1561.03)	1886.15(1471.22-2343.56)
Saint Kitts and Nevis	60.65(47.49-74.89)	28.32(20.56-38.11)	56.43(42.61-72.19)	73.41(57.53-90.61)	78.20(62.44-94.28)
Saint Lucia	89.01(62.68-114.99)	54.30(35.70-74.50)	59.65(40.01-79.49)	85.71(59.25-111.88)	138.78(99.93-176.35)
Saint Vincent and the Grenadines	58.11(44.74-71.29)	19.35(13.51-25.89)	33.18(24.04-42.29)	44.27(33.71-55.10)	127.06(99.16-154.85)
Samoa	3401.65(2433.38-4463.35)	1990.33(1310.98-2757.48)	3114.49(2127.50-4245.81)	3911.71(2837.86-5145.82)	4396.68(3331.64-5620.28)
San Marino	124.31(86.16-169.06)	102.26(65.81-146.37)	173.48(115.19-243.50)	136.44(97.86-183.76)	88.00(65.68-115.01)
Sao Tome and Principe	1762.23(1343.91-2181.27)	926.57(663.32-1194.63)	1247.29(910.59-1574.15)	1500.39(1140.06-1868.30)	3476.85(2707.53-4286.44)
Saudi Arabia	268.66(233.86-320.63)	145.66(115.32-194.20)	217.50(184.36-267.21)	276.98(241.90-328.07)	414.20(361.69-485.16)
Senegal	2033.36(1540.86-2469.31)	970.35(702.56-1212.61)	1577.28(1181.85-1922.35)	2040.34(1541.35-2488.57)	3945.39(3065.98-4771.75)
Serbia	187.89(141.08-232.54)	62.72(43.77-83.68)	87.61(62.90-112.83)	92.47(67.94-115.76)	482.75(367.89-596.61)
Seychelles	560.73(424.35-694.96)	299.39(214.22-402.58)	556.15(412.04-703.72)	902.45(687.74-1131.97)	499.33(385.79-623.04)
Sierra Leone	5341.11(3775.77-6375.47)	2124.70(1350.10-2693.07)	2865.47(1910.66-3489.04)	4214.14(2945.84-5028.50)	13774.03(9933.04-16226.95)
Singapore	200.58(171.11-247.04)	103.10(79.06-137.37)	181.64(149.81-236.29)	231.29(198.50-283.39)	305.91(269.90-355.50)
Slovakia	49.43(37.94-62.43)	39.54(28.30-54.15)	51.68(38.30-67.24)	50.72(39.51-63.67)	56.32(44.93-68.54)
Slovenia	78.85(59.39-99.63)	61.02(42.28-80.82)	106.44(78.25-137.95)	66.48(51.07-83.34)	78.90(62.65-96.43)
Solomon Islands	2334.64(1432.07-2981.35)	1290.06(668.41-1781.71)	2071.37(1183.72-2748.52)	2757.39(1718.87-3525.20)	3669.65(2407.14-4603.20)
Somalia	8446.34(7471.11-9575.80)	4387.18(3636.51-5259.58)	6936.54(6039.63-7997.39)	11996.65(10653.87-13624.22)	12957.95(11660.67-14464.32)
South Africa	2326.96(1980.78-2664.13)	1966.68(1627.66-2335.50)	2424.34(2043.60-2841.42)	2388.80(2038.20-2754.20)	2555.36(2176.11-2922.83)
South Sudan	5348.99(4071.56-6984.27)	2555.39(1873.70-3407.32)	4292.02(3208.51-5747.07)	7508.63(5663.87-9909.25)	7855.63(6034.68-10249.74)
Spain	61.88(51.18-75.56)	29.23(21.49-39.47)	55.87(44.28-72.24)	66.88(55.58-81.62)	91.67(77.91-107.65)
Sri Lanka	266.02(219.87-317.09)	74.89(56.73-95.12)	112.43(89.40-140.23)	166.13(135.95-202.09)	692.79(580.10-824.10)
Sudan	2511.80(2078.76-2987.91)	1193.85(938.23-1506.80)	1917.53(1544.78-2347.51)	2468.64(2048.02-2947.58)	4894.98(4129.24-5710.73)
Suriname	117.21(90.02-142.25)	37.25(27.43-49.75)	55.27(41.74-69.65)	85.62(65.32-105.07)	295.48(228.91-360.31)
Sweden	391.67(304.51-480.44)	77.87(55.17-100.89)	291.87(218.60-379.14)	586.04(455.14-723.09)	624.56(497.14-764.72)
Switzerland	525.71(390.60-665.75)	151.14(98.08-204.70)	492.65(346.34-650.90)	703.00(522.17-894.20)	773.44(597.68-955.68)

Syrian Arab Republic	1315.64(1178.25-1431.50)	821.79(669.22-964.41)	2088.55(1809.13-2339.19)	1101.36(1001.81-1193.35)	1298.73(1220.59-1376.62)
Taiwan (Province of China)	356.41(332.07-381.91)	155.53(132.54-180.12)	275.33(251.44-302.03)	382.14(356.67-409.70)	561.84(529.06-595.53)
Tajikistan	609.12(489.23-728.87)	291.93(219.26-368.61)	454.76(359.63-558.10)	654.64(525.42-781.69)	1196.89(977.57-1405.24)
Thailand	592.62(507.36-688.49)	361.92(283.81-448.98)	576.91(483.20-687.35)	743.84(641.49-870.98)	634.88(559.85-725.72)
Timor-Leste	2628.40(2063.72-3311.56)	1348.73(1011.87-1803.67)	1821.36(1405.10-2356.64)	2017.14(1574.66-2572.92)	5484.92(4304.43-6867.63)
Togo	2625.76(2188.47-3127.77)	843.04(648.93-1048.26)	1329.00(1073.63-1629.42)	2257.16(1868.21-2712.66)	7242.91(6049.05-8571.84)
Tokelau	4518.31(3367.24-5915.17)	2973.54(1985.15-4117.58)	4488.54(3263.00-5902.92)	5309.39(4021.47-6885.73)	5939.58(4627.56-7533.14)
Tonga	595.62(397.33-744.26)	411.09(236.97-551.35)	597.78(384.18-763.44)	599.53(411.93-739.48)	801.27(579.84-971.11)
Trinidad and Tobago	140.44(106.67-178.39)	60.79(41.90-84.05)	99.34(71.69-132.22)	149.64(112.34-191.80)	247.66(194.81-307.91)
Tunisia	370.93(321.17-423.35)	272.75(222.29-329.89)	393.30(334.25-458.64)	401.15(348.83-461.17)	418.95(367.92-474.70)
Turkey	431.00(387.35-474.36)	169.09(139.21-199.53)	256.18(222.22-289.10)	370.00(330.22-410.73)	818.72(745.07-897.97)
Turkmenistan	526.57(410.64-628.96)	377.54(279.15-464.59)	549.32(420.27-666.18)	616.46(485.31-738.66)	602.38(484.60-712.84)
Tuvalu	2679.82(2029.70-3365.96)	1482.98(1034.55-2004.36)	2383.23(1762.29-3102.50)	2718.34(2063.52-3430.05)	4046.55(3155.91-4989.27)
Uganda	1938.74(1638.49-2263.28)	891.35(684.43-1128.35)	1501.16(1219.76-1805.36)	2357.51(1999.00-2776.96)	3552.83(3074.73-4082.41)
Ukraine	482.27(410.39-568.44)	291.74(233.42-352.48)	392.62(327.96-471.03)	799.79(682.81-948.96)	427.16(364.96-497.84)
United Arab Emirates	226.99(173.06-285.57)	124.67(87.10-165.33)	170.46(125.74-221.23)	278.20(211.20-352.85)	339.99(264.51-419.99)
United Kingdom	509.51(405.77-638.43)	69.57(51.44-89.60)	568.20(434.01-732.71)	673.73(536.98-843.10)	734.45(605.50-891.71)
United Republic of Tanzania	838.26(779.26-918.61)	375.41(310.87-454.15)	615.31(553.43-699.30)	1021.23(957.35-1108.87)	1625.87(1552.99-1720.88)
United States of America	59.71(47.62-74.06)	31.61(23.32-39.97)	61.43(46.58-78.70)	71.75(57.28-88.96)	71.00(58.47-85.48)
United States Virgin Islands	129.24(99.49-164.88)	100.67(71.60-133.23)	154.12(113.85-201.07)	123.77(95.82-158.85)	137.59(109.60-172.37)
Uruguay	31.32(23.10-40.84)	14.46(8.88-19.78)	28.69(19.84-38.37)	36.17(26.77-47.61)	44.71(34.53-57.26)
Uzbekistan	900.45(695.56-1071.10)	418.23(287.80-522.63)	560.30(411.05-681.53)	782.84(603.19-938.89)	2064.74(1660.30-2446.66)
Vanuatu	2386.14(1733.06-3035.08)	1450.51(960.57-1953.02)	2115.99(1475.42-2748.19)	2668.18(1955.84-3409.02)	3642.58(2759.91-4579.39)

Venezuela	269.48(199.16-350.01)	145.05(100.65-196.58)	244.66(173.41-326.93)	319.34(235.34-417.20)	368.37(281.52-468.71)
Viet Nam	1549.61(1346.13-1780.41)	719.55(556.64-925.89)	1088.57(906.55-1305.47)	2032.12(1769.90-2332.62)	2395.95(2148.34-2675.02)
Yemen	2633.17(2227.74-3063.18)	1631.56(1275.67-2011.65)	2448.97(2028.43-2916.94)	2495.00(2133.85-2879.36)	4365.37(3808.90-4944.95)
Zambia	3232.10(2462.87-4087.22)	872.12(617.20-1146.67)	1578.21(1150.48-2089.34)	1724.69(1299.72-2212.92)	10261.96(7845.86-12913.90)
Zimbabwe	3039.87(2875.05-3250.75)	1774.03(1496.75-2094.99)	2220.37(2046.06-2423.98)	3149.96(3010.92-3335.37)	5527.16(5342.68-5766.98)

Supplementary Table 9: The DALYs of hepatitis B-associated cirrhosis in children and adolescents from 1990 to 2019 at the national and territorial levels

Location	<20 years	<5 years	5-9 years	10-14 years	15-19 years
Afghanistan	2.267(0.848-4.317)	1.163(0.251-3.107)	1.571(0.543-3.236)	2.504(0.904-5.351)	4.604(1.519-9.884)
Albania	0.980(0.365-1.945)	0.521(0.168-1.144)	0.817(0.265-1.613)	0.835(0.304-1.698)	1.615(0.532-3.424)
Algeria	0.743(0.266-1.478)	0.279(0.072-0.735)	0.435(0.155-0.974)	0.875(0.309-1.879)	1.645(0.521-3.620)
American Samoa	3.993(1.677-7.556)	0.090(0.034-0.201)	1.130(0.486-2.315)	2.989(1.201-5.943)	10.881(4.305-21.583)
Andorra	0.034(0.013-0.065)	0.010(0.003-0.024)	0.014(0.005-0.027)	0.026(0.010-0.050)	0.076(0.027-0.155)
Angola	4.758(1.769-9.691)	1.243(0.222-4.309)	2.149(0.622-5.313)	4.522(1.601-9.515)	15.079(5.262-32.324)
Antigua and Barbuda	0.139(0.037-0.297)	0.039(0.009-0.094)	0.070(0.020-0.154)	0.110(0.034-0.226)	0.298(0.080-0.671)
Argentina	0.229(0.076-0.441)	0.047(0.015-0.098)	0.087(0.032-0.171)	0.176(0.063-0.346)	0.599(0.192-1.209)
Armenia	0.671(0.262-1.240)	0.273(0.085-0.570)	0.355(0.141-0.673)	0.647(0.252-1.232)	1.597(0.586-3.363)
Australia	0.060(0.029-0.104)	0.017(0.007-0.032)	0.045(0.021-0.078)	0.054(0.026-0.091)	0.130(0.060-0.232)
Austria	0.035(0.014-0.064)	0.008(0.003-0.016)	0.016(0.006-0.031)	0.029(0.012-0.056)	0.085(0.032-0.164)
Azerbaijan	1.694(0.645-3.559)	0.093(0.023-0.291)	0.540(0.198-1.157)	1.327(0.522-2.792)	5.334(1.848-11.917)
Bahamas	0.187(0.049-0.404)	0.026(0.006-0.058)	0.059(0.018-0.118)	0.133(0.041-0.276)	0.469(0.114-1.034)
Bahrain	0.538(0.192-1.123)	0.142(0.045-0.314)	0.301(0.106-0.627)	0.597(0.207-1.291)	1.030(0.332-2.272)
Bangladesh	2.919(1.094-5.645)	0.875(0.235-2.288)	4.402(1.523-9.055)	2.994(1.095-5.891)	3.232(1.023-7.170)
Barbados	0.135(0.041-0.285)	0.011(0.004-0.025)	0.037(0.013-0.073)	0.097(0.031-0.195)	0.342(0.094-0.758)
Belarus	0.189(0.069-0.376)	0.055(0.017-0.125)	0.105(0.041-0.203)	0.176(0.069-0.343)	0.483(0.158-1.046)
Belgium	0.033(0.012-0.060)	0.009(0.003-0.019)	0.021(0.008-0.039)	0.030(0.011-0.057)	0.073(0.025-0.139)
Belize	0.323(0.082-0.673)	0.049(0.012-0.112)	0.099(0.028-0.200)	0.222(0.059-0.448)	0.856(0.195-1.914)
Benin	5.991(2.361-12.249)	2.451(0.723-6.021)	2.554(0.880-5.462)	5.460(2.112-11.696)	17.452(6.385-38.321)
Bermuda	0.149(0.062-0.282)	0.026(0.009-0.056)	0.051(0.023-0.094)	0.101(0.046-0.181)	0.397(0.150-0.796)
Bhutan	3.195(1.079-7.170)	0.799(0.214-2.100)	1.105(0.378-2.754)	1.801(0.643-4.354)	8.605(2.647-20.064)
Bolivia	0.290(0.075-0.677)	0.128(0.023-0.354)	0.147(0.038-0.339)	0.261(0.069-0.600)	0.725(0.166-1.820)
Bosnia and Herzegovina	0.441(0.197-0.860)	0.074(0.030-0.151)	0.165(0.075-0.296)	0.390(0.174-0.714)	1.011(0.400-2.069)

Botswana	1.204(0.398-2.601)	0.393(0.046-1.326)	0.405(0.141-0.845)	0.997(0.333-2.254)	3.223(0.992-7.361)
Brazil	0.368(0.210-0.569)	0.065(0.037-0.107)	0.132(0.082-0.203)	0.285(0.176-0.438)	0.964(0.522-1.547)
Brunei Darussalam	0.519(0.263-0.878)	0.066(0.031-0.117)	0.195(0.097-0.350)	0.427(0.213-0.735)	1.278(0.569-2.353)
Bulgaria	1.023(0.406-2.056)	0.151(0.054-0.330)	0.385(0.159-0.745)	0.862(0.354-1.644)	2.740(0.989-5.658)
Burkina Faso	5.763(1.995-12.714)	3.737(1.055-9.468)	2.642(0.810-6.274)	4.810(1.408-11.133)	14.900(4.476-34.615)
Burundi	1.645(0.564-3.773)	0.278(0.060-0.833)	0.791(0.243-1.968)	1.657(0.503-3.978)	5.101(1.501-12.527)
Cabo Verde	3.767(1.581-7.150)	0.433(0.102-1.228)	0.950(0.400-1.932)	2.347(0.999-4.338)	11.801(4.405-23.792)
Cambodia	4.859(1.839-9.820)	0.912(0.214-2.377)	1.332(0.477-2.854)	3.168(1.264-6.018)	15.376(5.090-33.618)
Cameroon	6.410(2.439-12.924)	1.612(0.458-3.925)	2.567(0.936-5.328)	5.489(2.045-11.289)	18.634(6.188-41.366)
Canada	0.027(0.007-0.057)	0.011(0.002-0.025)	0.011(0.003-0.023)	0.020(0.006-0.042)	0.065(0.017-0.139)
Central African Republic	4.577(1.669-9.778)	1.091(0.274-3.002)	1.824(0.680-4.060)	3.741(1.337-7.932)	13.974(4.670-32.648)
Chad	12.017(4.906-21.758)	4.309(1.047-11.121)	6.004(2.186-12.352)	11.951(4.752-23.405)	35.821(13.345-68.156)
Chile	0.142(0.050-0.277)	0.027(0.009-0.059)	0.059(0.023-0.117)	0.114(0.045-0.223)	0.362(0.125-0.771)
China	0.244(0.148-0.365)	0.041(0.024-0.066)	0.120(0.077-0.178)	0.190(0.122-0.276)	0.633(0.346-1.021)
Colombia	0.075(0.022-0.163)	0.023(0.005-0.056)	0.047(0.015-0.103)	0.074(0.022-0.153)	0.151(0.041-0.343)
Comoros	1.621(0.506-3.471)	0.293(0.053-1.129)	0.805(0.242-1.936)	1.647(0.532-3.646)	3.916(1.009-8.939)
Congo	2.708(1.035-5.409)	0.496(0.070-2.036)	1.010(0.335-2.516)	2.292(0.815-5.177)	8.478(2.877-17.616)
Cook Islands	0.454(0.164-0.957)	0.013(0.005-0.027)	0.101(0.038-0.220)	0.310(0.113-0.747)	1.392(0.447-3.125)
Costa Rica	0.095(0.024-0.206)	0.016(0.004-0.038)	0.039(0.012-0.081)	0.091(0.025-0.189)	0.227(0.057-0.516)
Croatia	0.165(0.077-0.294)	0.043(0.018-0.085)	0.076(0.034-0.139)	0.118(0.051-0.225)	0.407(0.169-0.769)
Cuba	0.119(0.035-0.238)	0.019(0.005-0.047)	0.046(0.015-0.099)	0.104(0.032-0.210)	0.283(0.077-0.588)
Cyprus	0.023(0.009-0.044)	0.012(0.003-0.028)	0.013(0.005-0.027)	0.019(0.007-0.036)	0.049(0.018-0.103)
Czechia	0.231(0.108-0.411)	0.052(0.023-0.095)	0.120(0.056-0.215)	0.222(0.106-0.383)	0.590(0.257-1.079)
C d'Ivoire	6.571(2.526-13.308)	1.909(0.506-4.590)	2.663(0.967-5.594)	6.032(2.372-11.875)	19.580(6.673-41.175)
Korea	0.745(0.277-1.552)	0.081(0.027-0.226)	0.198(0.080-0.456)	0.419(0.175-0.808)	2.137(0.633-5.037)
Democratic Republic of the Congo	3.557(1.305-7.571)	0.774(0.107-3.432)	1.564(0.428-4.427)	3.061(1.103-6.529)	10.752(3.885-24.020)

Denmark	0.055(0.025-0.104)	0.014(0.006-0.027)	0.028(0.012-0.057)	0.053(0.023-0.104)	0.120(0.053-0.228)
Djibouti	2.000(0.669-4.290)	0.474(0.118-1.391)	0.859(0.282-2.089)	2.061(0.683-4.778)	5.787(1.606-13.529)
Dominica	0.190(0.048-0.428)	0.031(0.006-0.081)	0.050(0.015-0.108)	0.164(0.044-0.396)	0.446(0.105-1.071)
Dominican Republic	0.739(0.177-1.712)	0.250(0.044-0.728)	0.441(0.105-1.017)	0.725(0.179-1.753)	1.612(0.338-4.109)
Ecuador	0.251(0.063-0.567)	0.044(0.010-0.117)	0.104(0.026-0.230)	0.243(0.057-0.545)	0.620(0.145-1.442)
Egypt	2.405(0.881-5.150)	0.421(0.078-1.532)	0.819(0.264-1.972)	2.313(0.816-5.081)	6.789(2.311-14.622)
El Salvador	0.144(0.034-0.338)	0.019(0.003-0.055)	0.056(0.016-0.128)	0.116(0.030-0.277)	0.381(0.081-0.968)
Equatorial Guinea	3.277(1.157-6.906)	0.527(0.065-2.320)	1.090(0.314-2.877)	2.461(0.799-6.005)	9.183(3.139-19.912)
Eritrea	2.324(0.782-4.988)	0.477(0.104-1.492)	0.771(0.212-1.892)	2.003(0.647-4.384)	6.733(2.049-16.028)
Estonia	0.158(0.061-0.292)	0.026(0.010-0.055)	0.045(0.019-0.084)	0.101(0.041-0.183)	0.511(0.169-1.025)
Eswatini	1.441(0.487-3.097)	0.236(0.048-0.615)	0.468(0.154-1.006)	1.173(0.392-2.623)	4.215(1.285-9.994)
Ethiopia	4.369(2.532-7.085)	1.049(0.356-2.565)	1.849(0.880-3.537)	3.979(2.227-6.680)	12.283(6.635-20.366)
Fiji	1.955(0.738-3.671)	0.093(0.031-0.214)	0.972(0.357-1.975)	2.286(0.794-4.416)	4.925(1.714-9.900)
Finland	0.052(0.024-0.093)	0.012(0.005-0.022)	0.029(0.013-0.052)	0.049(0.021-0.095)	0.114(0.050-0.208)
France	0.038(0.015-0.068)	0.009(0.004-0.017)	0.018(0.008-0.034)	0.032(0.013-0.061)	0.087(0.034-0.166)
Gabon	3.017(1.167-5.802)	0.496(0.063-2.131)	0.905(0.303-2.271)	2.144(0.787-4.411)	9.107(3.050-18.431)
Gambia	5.153(1.969-10.341)	1.176(0.270-3.503)	1.500(0.565-3.219)	3.634(1.519-7.651)	16.231(5.197-34.586)
Georgia	0.847(0.324-1.671)	0.065(0.022-0.138)	0.166(0.066-0.326)	0.547(0.205-1.101)	3.062(1.068-6.185)
Germany	0.039(0.015-0.074)	0.007(0.003-0.014)	0.019(0.007-0.037)	0.035(0.014-0.068)	0.093(0.035-0.181)
Ghana	5.615(2.104-10.785)	1.115(0.284-2.940)	1.655(0.631-3.319)	4.160(1.547-8.077)	17.168(5.904-34.423)
Greece	0.044(0.017-0.081)	0.012(0.004-0.024)	0.021(0.009-0.040)	0.037(0.015-0.070)	0.101(0.037-0.197)
Greenland	0.038(0.011-0.092)	0.015(0.003-0.041)	0.024(0.006-0.063)	0.033(0.010-0.077)	0.083(0.021-0.216)
Grenada	0.177(0.044-0.374)	0.019(0.005-0.046)	0.067(0.019-0.142)	0.131(0.037-0.266)	0.442(0.101-0.946)
Guam	4.676(2.011-8.494)	0.222(0.084-0.484)	1.499(0.655-2.814)	4.095(1.762-7.681)	14.312(5.592-27.609)
Guatemala	0.348(0.076-0.775)	0.076(0.016-0.183)	0.134(0.033-0.292)	0.260(0.059-0.591)	0.938(0.192-2.242)
Guinea	9.710(3.965-18.269)	3.739(0.936-9.938)	4.674(1.551-9.795)	8.930(3.309-18.817)	27.671(10.358-54.908)

Guinea-Bissau	9.707(3.688-18.656)	1.937(0.546-5.220)	3.244(1.217-6.815)	7.808(2.776-15.584)	31.129(10.690-64.358)
Guyana	0.456(0.108-0.967)	0.060(0.015-0.144)	0.203(0.053-0.423)	0.432(0.110-0.922)	1.105(0.258-2.508)
Haiti	0.747(0.165-1.855)	0.500(0.029-1.735)	0.525(0.100-1.387)	0.759(0.178-1.855)	1.296(0.294-3.288)
Honduras	0.316(0.071-0.770)	0.144(0.022-0.493)	0.218(0.043-0.606)	0.293(0.064-0.718)	0.623(0.124-1.622)
Hungary	0.450(0.226-0.751)	0.075(0.035-0.135)	0.183(0.088-0.334)	0.382(0.188-0.653)	1.108(0.537-1.966)
Iceland	0.035(0.014-0.070)	0.008(0.004-0.016)	0.021(0.008-0.044)	0.036(0.014-0.079)	0.073(0.030-0.146)
India	3.648(2.054-5.790)	0.688(0.340-1.254)	1.277(0.708-2.070)	2.224(1.299-3.396)	9.857(5.154-16.011)
Indonesia	3.760(2.115-6.000)	0.798(0.427-1.285)	0.918(0.544-1.562)	2.202(1.277-3.420)	10.634(5.518-17.916)
Iran	0.555(0.315-0.870)	0.173(0.070-0.366)	0.346(0.187-0.593)	0.614(0.342-0.960)	1.249(0.650-2.010)
Iraq	0.766(0.302-1.494)	0.241(0.072-0.558)	0.486(0.183-0.961)	0.737(0.301-1.370)	1.644(0.583-3.533)
Ireland	0.043(0.018-0.080)	0.007(0.003-0.014)	0.018(0.007-0.034)	0.032(0.013-0.062)	0.117(0.043-0.241)
Israel	0.044(0.016-0.083)	0.015(0.005-0.030)	0.031(0.011-0.060)	0.046(0.017-0.088)	0.094(0.032-0.194)
Italy	0.065(0.042-0.094)	0.014(0.009-0.022)	0.030(0.020-0.045)	0.057(0.037-0.082)	0.148(0.090-0.225)
Jamaica	0.145(0.039-0.332)	0.017(0.005-0.039)	0.059(0.018-0.139)	0.138(0.039-0.333)	0.322(0.084-0.747)
Japan	0.190(0.124-0.278)	0.055(0.034-0.082)	0.108(0.067-0.170)	0.176(0.110-0.276)	0.382(0.238-0.560)
Jordan	0.405(0.162-0.781)	0.101(0.031-0.245)	0.237(0.090-0.471)	0.423(0.172-0.830)	0.871(0.322-1.787)
Kazakhstan	0.739(0.293-1.426)	0.131(0.043-0.320)	0.332(0.125-0.642)	0.684(0.265-1.332)	2.352(0.846-4.891)
Kenya	2.033(1.047-3.488)	0.147(0.062-0.289)	0.497(0.260-0.871)	1.530(0.829-2.531)	6.322(3.025-11.161)
Kiribati	5.630(1.602-13.034)	0.177(0.029-0.524)	2.139(0.486-5.303)	5.205(1.473-11.775)	17.833(4.500-43.995)
Kuwait	0.208(0.083-0.381)	0.046(0.018-0.096)	0.072(0.031-0.134)	0.293(0.117-0.549)	0.486(0.179-0.950)
Kyrgyzstan	1.364(0.523-2.568)	0.114(0.045-0.225)	0.466(0.199-0.902)	1.313(0.523-2.525)	4.463(1.572-8.775)
Lao People's Democratic Republic	1.681(0.646-3.381)	0.263(0.071-0.667)	0.813(0.279-1.761)	1.295(0.482-2.655)	4.582(1.638-9.479)
Latvia	0.160(0.060-0.305)	0.020(0.008-0.043)	0.061(0.026-0.110)	0.101(0.042-0.183)	0.506(0.172-1.021)
Lebanon	0.787(0.277-1.808)	0.224(0.050-0.769)	0.383(0.125-0.964)	0.760(0.276-1.786)	2.215(0.749-5.142)
Lesotho	1.427(0.465-3.155)	0.240(0.046-0.747)	0.456(0.146-1.061)	1.069(0.310-2.625)	3.937(1.217-9.612)
Liberia	10.158(3.569-21.827)	2.787(0.347-11.232)	5.091(1.444-13.609)	9.253(3.246-20.707)	26.001(9.390-54.273)

Libya	0.730(0.241-1.643)	0.359(0.080-0.946)	0.344(0.112-0.863)	0.550(0.185-1.305)	1.490(0.463-3.452)
Lithuania	0.143(0.056-0.269)	0.020(0.008-0.042)	0.049(0.021-0.091)	0.095(0.040-0.177)	0.413(0.145-0.859)
Luxembourg	0.047(0.019-0.089)	0.008(0.003-0.016)	0.022(0.009-0.042)	0.040(0.015-0.080)	0.118(0.045-0.236)
Madagascar	1.974(0.740-4.154)	0.273(0.061-0.911)	0.726(0.245-1.771)	1.866(0.662-3.929)	5.924(1.864-13.383)
Malawi	2.809(0.974-5.859)	0.540(0.130-1.457)	0.975(0.327-2.243)	2.396(0.800-5.324)	7.974(2.432-17.587)
Malaysia	1.184(0.537-2.161)	0.094(0.024-0.273)	0.408(0.185-0.774)	0.921(0.436-1.653)	3.187(1.341-6.209)
Maldives	0.386(0.166-0.715)	0.193(0.055-0.452)	0.267(0.107-0.514)	0.328(0.149-0.596)	0.857(0.339-1.767)
Mali	7.400(3.024-15.232)	4.095(0.815-11.461)	3.459(1.102-7.629)	6.478(2.502-13.554)	19.414(7.633-43.275)
Malta	0.032(0.012-0.062)	0.005(0.002-0.011)	0.015(0.006-0.030)	0.027(0.010-0.051)	0.082(0.029-0.167)
Marshall Islands	3.071(0.927-6.730)	0.087(0.026-0.228)	1.116(0.355-2.582)	2.591(0.809-5.668)	9.147(2.546-20.759)
Mauritania	4.987(1.977-10.390)	0.701(0.159-2.200)	2.175(0.776-4.727)	4.408(1.670-9.245)	14.194(5.030-31.928)
Mauritius	0.892(0.350-1.704)	0.096(0.035-0.202)	0.324(0.127-0.623)	0.529(0.229-0.978)	2.181(0.766-4.348)
Mexico	0.139(0.074-0.218)	0.044(0.023-0.076)	0.055(0.032-0.085)	0.109(0.063-0.166)	0.343(0.169-0.585)
Micronesia (Federated States of)	3.372(1.093-7.288)	0.075(0.017-0.200)	0.942(0.193-2.148)	2.412(0.618-5.600)	9.501(2.714-21.634)
Monaco	0.042(0.015-0.082)	0.010(0.003-0.026)	0.015(0.006-0.029)	0.030(0.012-0.056)	0.105(0.034-0.223)
Mongolia	1.490(0.537-3.133)	0.352(0.084-1.053)	0.968(0.322-2.215)	1.558(0.499-3.557)	4.296(1.395-9.743)
Montenegro	0.184(0.087-0.337)	0.030(0.012-0.059)	0.082(0.034-0.159)	0.170(0.074-0.313)	0.418(0.175-0.809)
Morocco	0.753(0.257-1.546)	0.254(0.061-0.696)	0.408(0.138-0.899)	0.740(0.246-1.569)	1.607(0.497-3.518)
Mozambique	2.226(0.661-5.092)	0.364(0.055-1.306)	0.601(0.166-1.733)	1.900(0.554-4.943)	7.904(2.142-19.124)
Myanmar	2.136(0.788-4.178)	0.182(0.049-0.527)	0.265(0.102-0.621)	0.565(0.242-1.102)	7.564(2.630-14.980)
Namibia	0.992(0.329-2.123)	0.181(0.039-0.519)	0.312(0.109-0.680)	0.771(0.251-1.693)	3.027(0.897-6.900)
Nauru	3.304(0.953-7.888)	0.185(0.036-0.549)	1.385(0.385-3.446)	2.953(0.934-6.830)	9.749(2.527-24.068)
Nepal	3.262(1.054-6.960)	0.315(0.073-0.953)	0.990(0.328-2.250)	1.654(0.584-3.644)	9.580(2.915-21.147)
Netherlands	0.045(0.019-0.083)	0.009(0.004-0.016)	0.023(0.010-0.043)	0.046(0.020-0.085)	0.095(0.039-0.180)
New Zealand	0.079(0.051-0.111)	0.019(0.011-0.031)	0.042(0.027-0.061)	0.065(0.042-0.094)	0.190(0.117-0.280)
Nicaragua	0.184(0.043-0.425)	0.060(0.012-0.153)	0.091(0.022-0.213)	0.167(0.042-0.388)	0.432(0.094-1.040)

Niger	7.732(3.155-15.472)	3.913(0.840-10.781)	3.419(1.274-7.309)	7.069(2.731-15.666)	22.633(8.188-50.673)
Nigeria	9.856(5.366-17.366)	5.416(2.589-10.008)	4.978(2.651-8.388)	8.492(4.714-14.308)	24.030(11.180-47.944)
Niue	2.869(0.966-6.445)	0.177(0.027-0.637)	0.955(0.292-2.178)	2.387(0.788-5.208)	7.756(2.306-18.162)
North Macedonia	0.522(0.224-0.994)	0.101(0.040-0.222)	0.237(0.105-0.433)	0.524(0.222-0.997)	1.186(0.448-2.380)
Northern Mariana Islands	6.990(2.852-13.129)	0.301(0.096-0.708)	1.800(0.744-3.421)	4.425(1.784-8.593)	15.020(5.914-29.190)
Norway	0.032(0.018-0.051)	0.006(0.003-0.010)	0.014(0.007-0.025)	0.030(0.016-0.050)	0.077(0.042-0.123)
Oman	0.506(0.183-1.002)	0.153(0.041-0.384)	0.244(0.085-0.518)	0.556(0.202-1.140)	1.352(0.441-2.788)
Pakistan	5.412(3.052-8.646)	0.904(0.414-1.806)	2.884(1.383-5.313)	4.298(2.345-6.884)	15.194(7.830-26.043)
Palau	2.543(0.899-5.259)	0.107(0.024-0.301)	0.789(0.273-1.644)	1.729(0.653-3.568)	7.154(2.243-15.853)
Palestine	0.628(0.234-1.212)	0.200(0.063-0.470)	0.388(0.140-0.766)	0.609(0.229-1.177)	1.459(0.516-2.870)
Panama	0.137(0.033-0.295)	0.036(0.008-0.087)	0.057(0.016-0.125)	0.142(0.035-0.305)	0.333(0.073-0.766)
Papua New Guinea	1.954(0.698-4.132)	0.272(0.062-0.724)	0.976(0.366-2.079)	2.027(0.737-4.428)	5.652(1.727-13.077)
Paraguay	0.402(0.135-0.895)	0.025(0.007-0.080)	0.136(0.050-0.313)	0.332(0.117-0.732)	1.117(0.348-2.674)
Peru	0.347(0.095-0.780)	0.050(0.010-0.159)	0.153(0.045-0.374)	0.294(0.084-0.647)	0.930(0.237-2.214)
Philippines	0.788(0.444-1.223)	0.182(0.099-0.311)	0.433(0.254-0.731)	0.621(0.364-0.997)	2.062(1.073-3.393)
Poland	0.216(0.133-0.317)	0.038(0.022-0.059)	0.096(0.060-0.145)	0.188(0.120-0.273)	0.564(0.325-0.879)
Portugal	0.044(0.015-0.085)	0.006(0.002-0.012)	0.015(0.006-0.029)	0.032(0.012-0.060)	0.103(0.032-0.217)
Puerto Rico	0.088(0.029-0.182)	0.013(0.004-0.029)	0.027(0.010-0.052)	0.057(0.021-0.114)	0.207(0.065-0.455)
Qatar	0.639(0.219-1.302)	0.152(0.045-0.385)	0.227(0.079-0.496)	0.629(0.232-1.384)	1.871(0.553-4.100)
South Korea	1.046(0.599-1.668)	0.227(0.104-0.445)	0.591(0.323-0.966)	0.966(0.525-1.602)	2.184(1.192-3.499)
Republic of Moldova	0.485(0.173-0.933)	0.045(0.016-0.091)	0.135(0.054-0.260)	0.436(0.159-0.838)	1.283(0.413-2.530)
Romania	0.693(0.289-1.297)	0.108(0.043-0.217)	0.236(0.110-0.431)	0.541(0.246-0.985)	1.831(0.729-3.623)
Russian Federation	0.168(0.097-0.256)	0.027(0.016-0.043)	0.068(0.043-0.101)	0.137(0.085-0.202)	0.505(0.267-0.837)
Rwanda	1.187(0.336-2.697)	0.330(0.059-1.156)	0.483(0.132-1.243)	1.054(0.290-2.448)	3.153(0.865-7.496)
Saint Kitts and Nevis	0.181(0.048-0.396)	0.023(0.004-0.059)	0.070(0.020-0.156)	0.143(0.042-0.311)	0.444(0.108-0.993)
Saint Lucia	0.192(0.050-0.398)	0.027(0.007-0.064)	0.071(0.021-0.146)	0.153(0.043-0.304)	0.435(0.109-0.946)

Saint Vincent and the Grenadines	0.168(0.045-0.357)	0.022(0.006-0.053)	0.043(0.015-0.088)	0.150(0.043-0.319)	0.421(0.105-0.938)
Samoa	3.498(1.410-6.710)	0.081(0.028-0.199)	1.088(0.421-2.184)	2.853(1.146-5.529)	9.891(3.477-20.755)
San Marino	0.061(0.022-0.126)	0.024(0.006-0.055)	0.026(0.010-0.049)	0.044(0.017-0.081)	0.138(0.045-0.316)
Sao Tome and Principe	8.442(3.508-16.135)	1.587(0.280-5.737)	2.709(1.009-6.054)	5.682(2.205-11.773)	24.864(8.929-50.216)
Saudi Arabia	0.251(0.089-0.528)	0.042(0.011-0.132)	0.082(0.031-0.170)	0.183(0.071-0.362)	0.648(0.209-1.454)
Senegal	5.360(2.050-10.990)	1.076(0.282-3.055)	2.028(0.749-4.269)	4.472(1.791-9.741)	15.931(5.579-35.323)
Serbia	0.195(0.094-0.346)	0.034(0.013-0.074)	0.102(0.044-0.190)	0.173(0.083-0.319)	0.441(0.189-0.864)
Seychelles	0.745(0.320-1.395)	0.166(0.055-0.386)	0.414(0.154-0.895)	0.547(0.236-1.044)	1.915(0.746-3.828)
Sierra Leone	6.640(2.460-13.287)	2.965(0.663-7.536)	2.940(0.859-6.975)	5.511(1.914-11.537)	17.143(6.048-35.776)
Singapore	0.527(0.297-0.803)	0.123(0.061-0.216)	0.216(0.115-0.360)	0.542(0.305-0.836)	1.311(0.714-2.098)
Slovakia	0.341(0.161-0.623)	0.099(0.040-0.198)	0.142(0.064-0.266)	0.310(0.146-0.575)	0.851(0.353-1.666)
Slovenia	0.129(0.059-0.231)	0.022(0.009-0.042)	0.064(0.025-0.133)	0.133(0.058-0.253)	0.320(0.142-0.573)
Solomon Islands	2.748(1.049-5.551)	0.122(0.039-0.317)	0.438(0.172-0.974)	1.931(0.739-3.982)	10.333(3.590-22.179)
Somalia	6.036(2.384-11.933)	1.059(0.299-2.958)	2.435(0.906-5.176)	6.397(2.267-12.744)	18.555(6.072-41.550)
South Africa	0.456(0.258-0.764)	0.150(0.075-0.266)	0.294(0.175-0.463)	0.208(0.090-0.451)	1.244(0.564-2.350)
South Sudan	3.859(1.446-7.859)	1.215(0.337-3.330)	1.560(0.562-3.432)	3.746(1.330-8.399)	10.282(3.478-22.291)
Spain	0.057(0.024-0.100)	0.012(0.005-0.022)	0.025(0.011-0.045)	0.049(0.021-0.088)	0.138(0.056-0.259)
Sri Lanka	0.704(0.279-1.378)	0.129(0.044-0.290)	0.362(0.135-0.698)	0.552(0.233-1.060)	1.713(0.606-3.726)
Sudan	1.789(0.611-3.923)	1.060(0.219-2.806)	1.163(0.356-2.750)	1.855(0.591-4.103)	3.358(1.019-8.540)
Suriname	0.284(0.070-0.641)	0.050(0.010-0.139)	0.095(0.025-0.211)	0.266(0.061-0.626)	0.733(0.168-1.760)
Sweden	0.050(0.030-0.076)	0.012(0.007-0.019)	0.028(0.017-0.045)	0.058(0.035-0.089)	0.106(0.058-0.170)
Switzerland	0.039(0.018-0.070)	0.008(0.004-0.016)	0.019(0.009-0.038)	0.036(0.016-0.071)	0.093(0.040-0.178)
Syrian Arab Republic	1.634(0.578-3.173)	0.515(0.152-1.176)	0.878(0.293-1.852)	1.471(0.489-3.034)	2.986(0.980-6.163)
Taiwan (Province of China)	0.443(0.213-0.792)	0.098(0.042-0.194)	0.182(0.091-0.308)	0.341(0.164-0.592)	1.017(0.437-1.922)
Tajikistan	2.535(0.908-5.065)	0.321(0.087-0.869)	0.935(0.318-2.008)	2.472(0.885-4.933)	7.717(2.596-16.039)
Thailand	0.386(0.144-0.792)	0.073(0.019-0.186)	0.141(0.054-0.290)	0.240(0.095-0.464)	0.952(0.330-2.071)

Timor-Leste	1.275(0.493-2.490)	0.262(0.041-0.691)	0.548(0.175-1.209)	0.873(0.323-1.805)	3.545(1.179-7.317)
Togo	5.889(2.294-11.136)	1.245(0.324-3.412)	2.200(0.798-4.679)	4.857(1.869-9.783)	18.413(6.285-37.386)
Tokelau	3.176(1.289-6.302)	0.104(0.036-0.231)	1.137(0.430-2.525)	2.877(1.190-5.958)	10.718(4.080-21.758)
Tonga	2.369(0.913-4.955)	0.123(0.038-0.315)	0.884(0.328-1.851)	2.096(0.778-4.304)	6.969(2.515-15.542)
Trinidad and Tobago	0.184(0.050-0.415)	0.058(0.014-0.131)	0.077(0.023-0.163)	0.144(0.046-0.308)	0.457(0.119-1.088)
Tunisia	0.247(0.054-0.649)	0.108(0.017-0.335)	0.156(0.038-0.409)	0.269(0.061-0.696)	0.477(0.101-1.399)
Turkey	0.676(0.301-1.241)	0.368(0.116-0.853)	0.419(0.173-0.823)	0.596(0.264-1.062)	1.179(0.492-2.257)
Turkmenistan	4.150(1.542-8.409)	0.337(0.105-0.749)	1.381(0.503-2.706)	3.821(1.430-7.859)	13.394(4.658-27.184)
Tuvalu	3.365(1.268-6.618)	0.075(0.026-0.170)	1.145(0.429-2.342)	2.752(1.036-5.571)	9.258(3.081-19.835)
Uganda	1.739(0.617-3.523)	0.393(0.093-1.201)	0.668(0.223-1.516)	1.628(0.558-3.502)	5.238(1.535-11.818)
Ukraine	0.684(0.341-1.188)	0.076(0.039-0.129)	0.179(0.104-0.278)	0.464(0.255-0.751)	2.167(0.930-4.083)
United Arab Emirates	0.109(0.048-0.202)	0.013(0.005-0.028)	0.048(0.016-0.106)	0.096(0.035-0.203)	0.296(0.123-0.552)
United Kingdom	0.057(0.032-0.093)	0.012(0.007-0.020)	0.025(0.015-0.039)	0.044(0.026-0.067)	0.155(0.078-0.266)
United Republic of Tanzania	1.732(0.595-3.748)	0.737(0.153-2.255)	0.927(0.266-2.191)	1.753(0.571-3.978)	4.293(1.191-9.959)
United States of America	0.021(0.010-0.034)	0.005(0.003-0.009)	0.006(0.003-0.010)	0.012(0.006-0.019)	0.056(0.026-0.095)
United States Virgin Islands	0.164(0.050-0.354)	0.023(0.005-0.069)	0.065(0.021-0.148)	0.136(0.041-0.279)	0.435(0.117-0.976)
Uruguay	0.117(0.044-0.225)	0.046(0.015-0.105)	0.055(0.021-0.106)	0.080(0.033-0.146)	0.273(0.095-0.559)
Uzbekistan	2.792(1.050-5.364)	0.194(0.067-0.411)	1.121(0.398-2.157)	2.594(0.968-5.102)	8.388(2.869-16.864)
Vanuatu	3.331(1.142-7.396)	0.111(0.031-0.298)	1.370(0.403-3.135)	3.247(1.097-7.235)	10.328(3.344-24.290)
Venezuela	0.108(0.028-0.246)	0.022(0.006-0.052)	0.039(0.012-0.087)	0.096(0.028-0.206)	0.280(0.067-0.694)
Viet Nam	1.236(0.479-2.555)	0.226(0.057-0.588)	0.259(0.096-0.621)	0.776(0.300-1.618)	3.813(1.378-8.383)
Yemen	1.235(0.423-2.549)	0.607(0.149-1.512)	0.960(0.313-2.076)	1.559(0.530-3.390)	2.030(0.554-4.639)
Zambia	3.339(1.173-6.812)	0.653(0.114-2.293)	1.141(0.358-2.967)	2.746(0.858-5.903)	10.466(3.304-22.493)
Zimbabwe	1.671(0.519-3.896)	0.240(0.038-0.714)	0.258(0.094-0.5521)	1.080(0.361-2.378)	5.870(1.574-14.628)

Figure S1 Composition ratio of incidence of liver cirrhosis in different age groups at global and regional levels

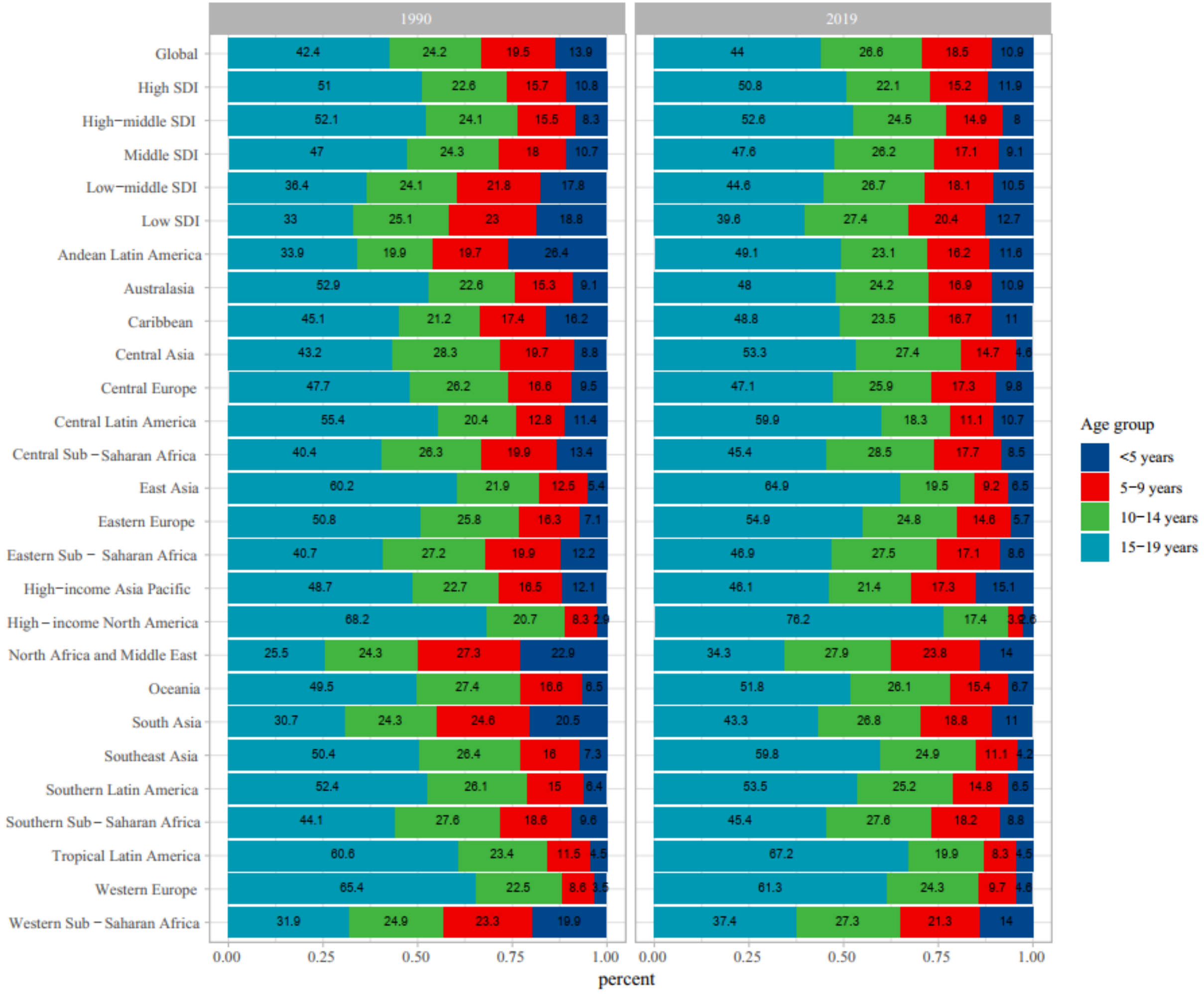

Figure S2 Trends of incidence rate of liver cirrhosis in different age groups from 1990 to 2019

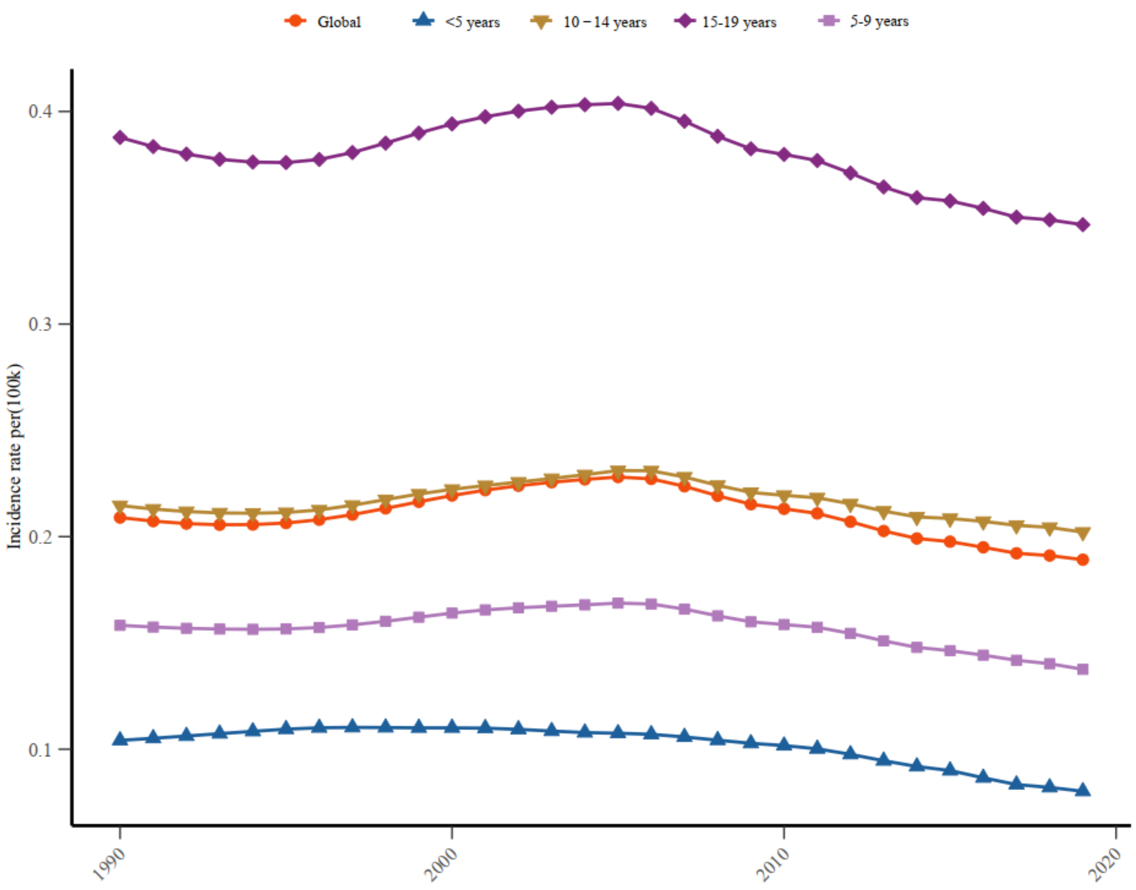

Figure S3 Trends of incidence rate of liver cirrhosis in males of different age groups from 1990 to 2019

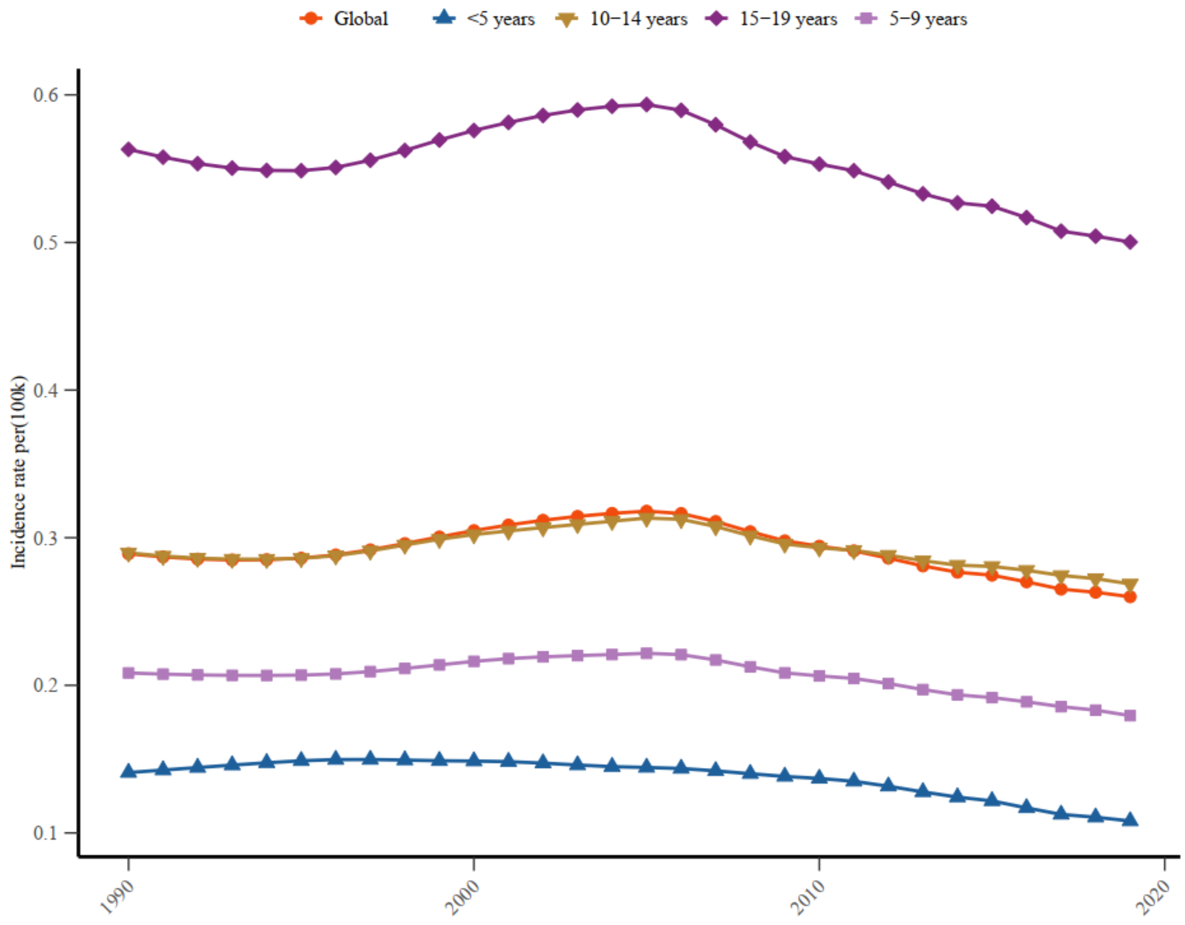

Figure S4 Trends of incidence rate of liver cirrhosis in females of different age groups from 1990 to 2019

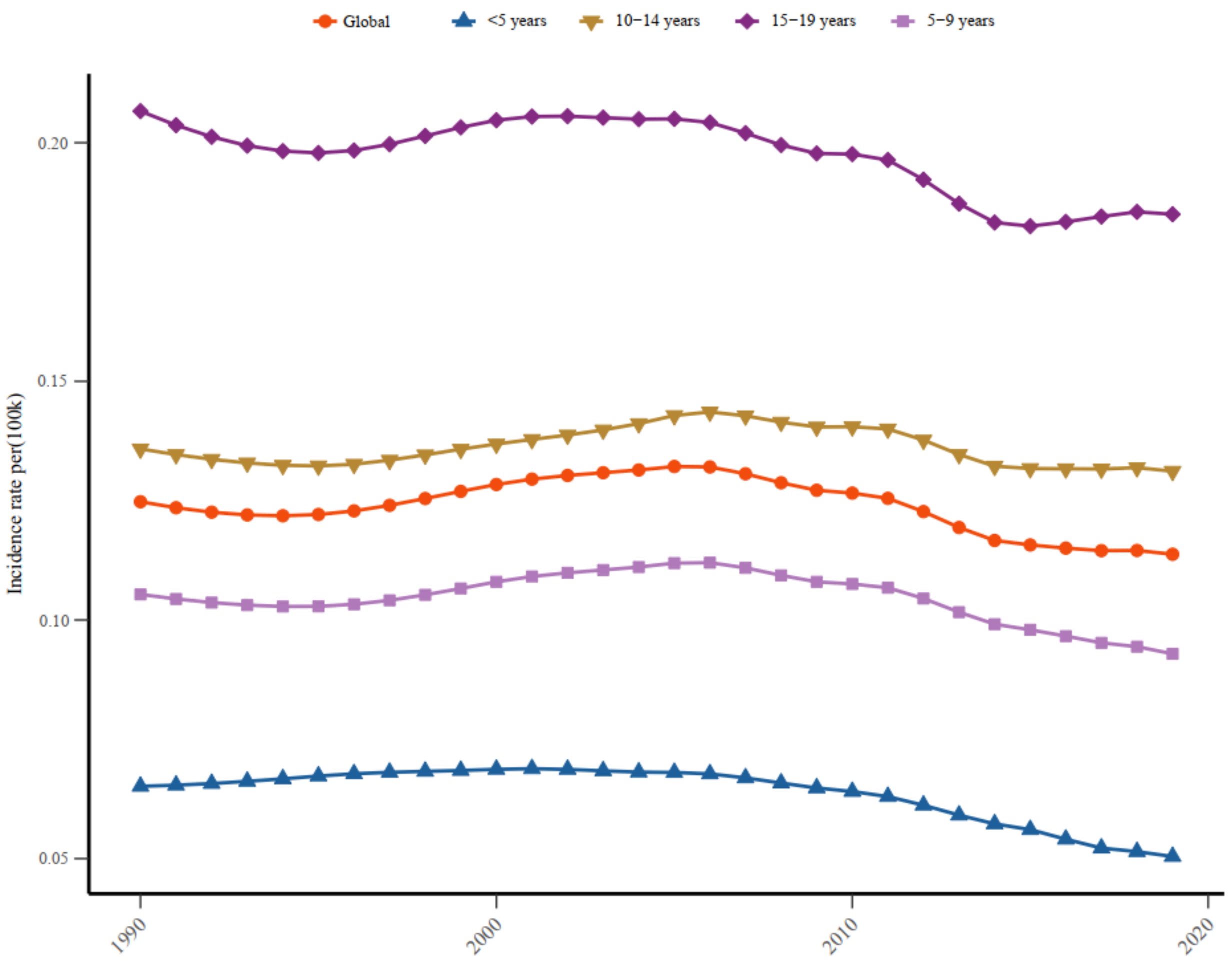

Figure S5 Composition ratio of prevalence of liver cirrhosis in different age groups at global and regional levels

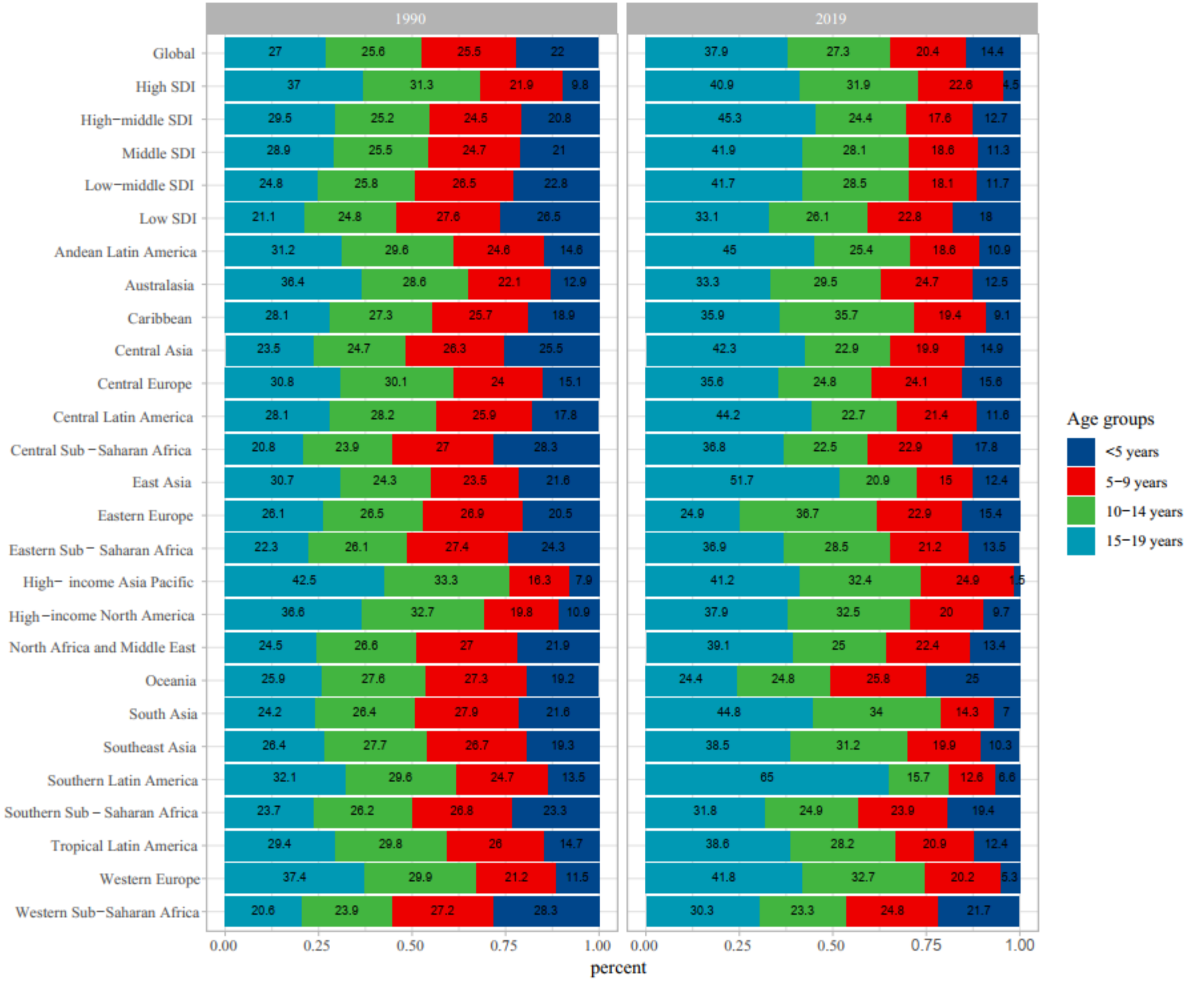

Figure S6 Trends of prevalence rate of liver cirrhosis in different age groups from 1990 to 2019

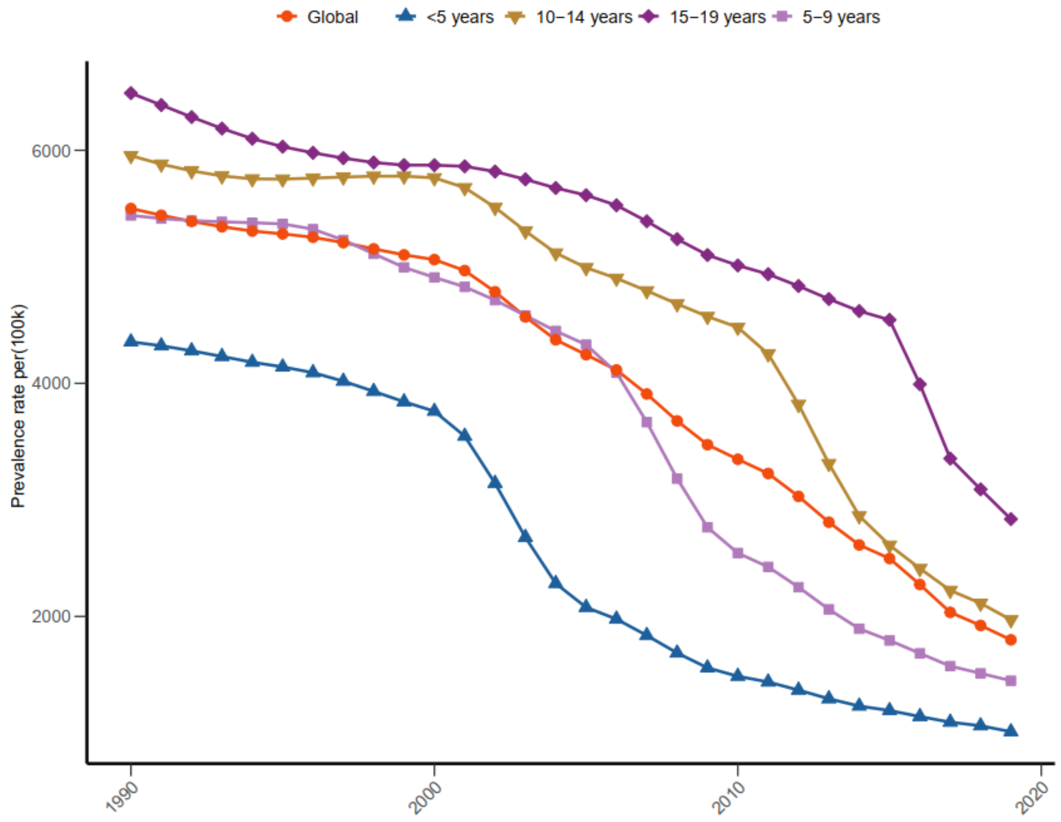

Figure S7 Trends of prevalence rate of liver cirrhosis in males of different age groups from 1990 to 2019

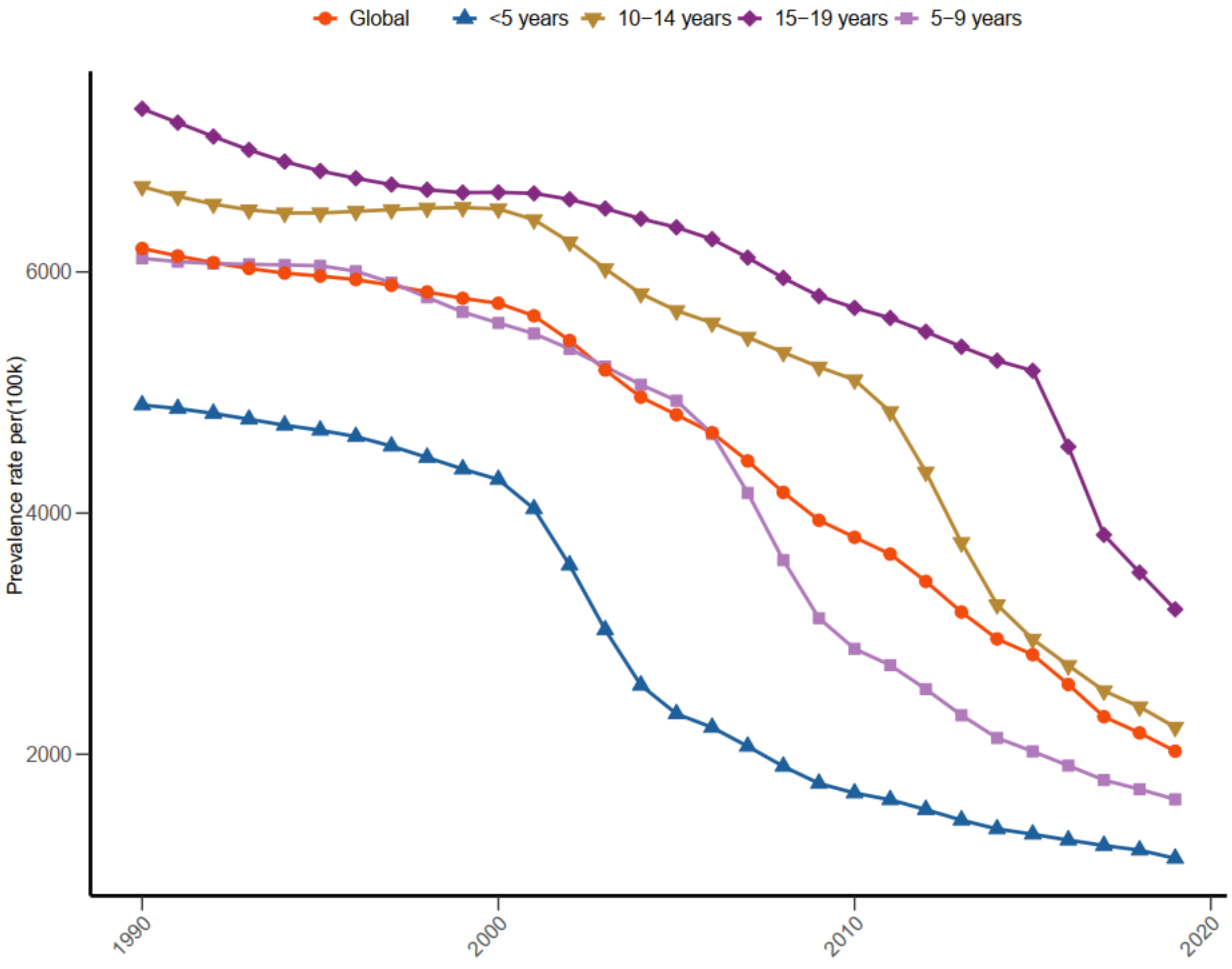

Figure S8 Trends of prevalence rate of liver cirrhosis in females of different age groups from 1990 to 2019

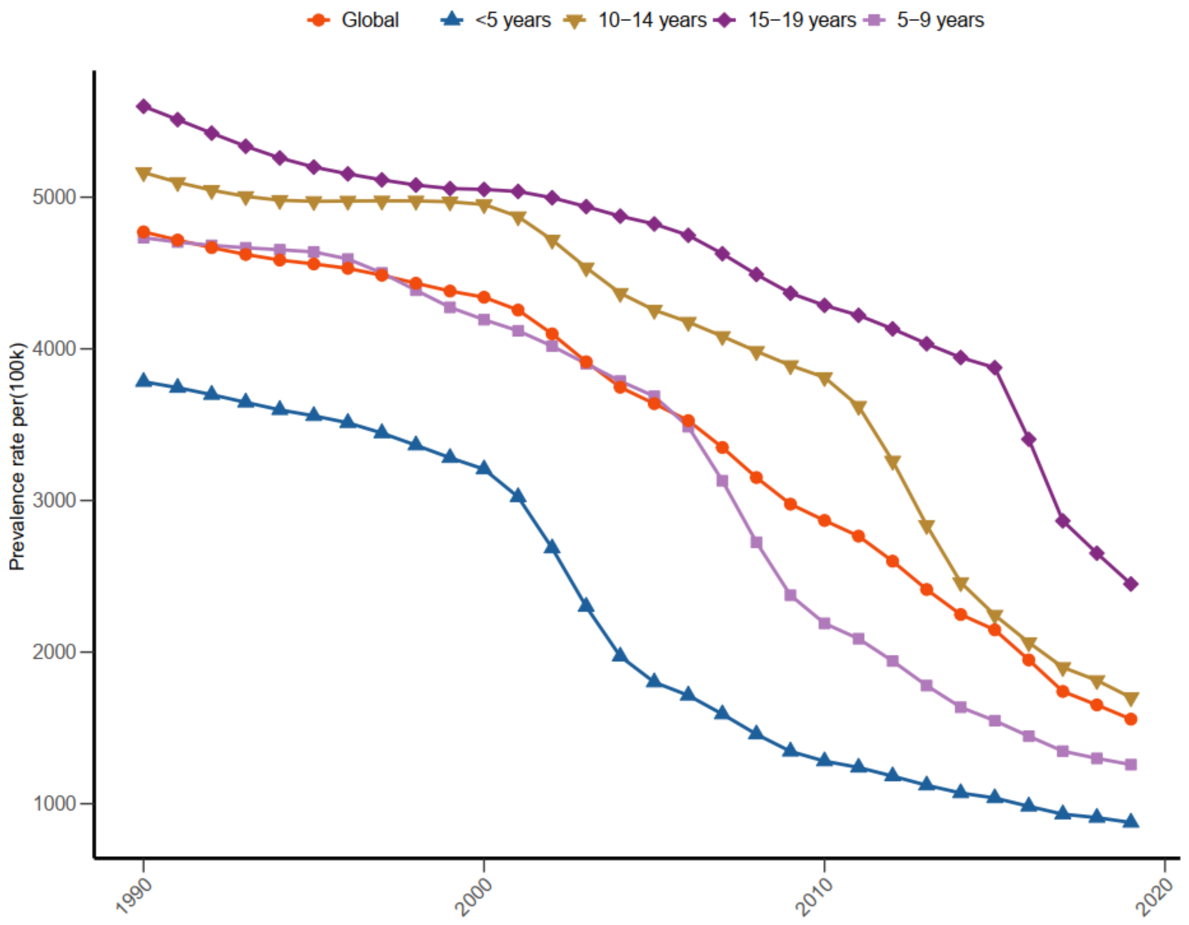

Supplement: Supplementary file 1 [file Data_Sheet_1.PDF]
